# Supplementary material for: TLR3 activation enhances abscopal effect of radiotherapy in HCC by promoting tumor ferroptosis
Source: EMBO Mol Med. 2024 Apr 26;16(5):8. doi: 10.1038/s44321-024-00068-4 (PMC11098818; doi:10.1038/s44321-024-00068-4)
Supplement: Supplementary file 1 — Appendix [file 44321_2024_68_MOESM1_ESM.pdf]

Appendix Data

Table of contents:

APPENDIX FIGURES	PAGE
Appendix Figure S1	1
Appendix Figure S2	1
Appendix Figure S3	2
Appendix Figure S4	2
Appendix Figure S5	3

Appendix Figure S1

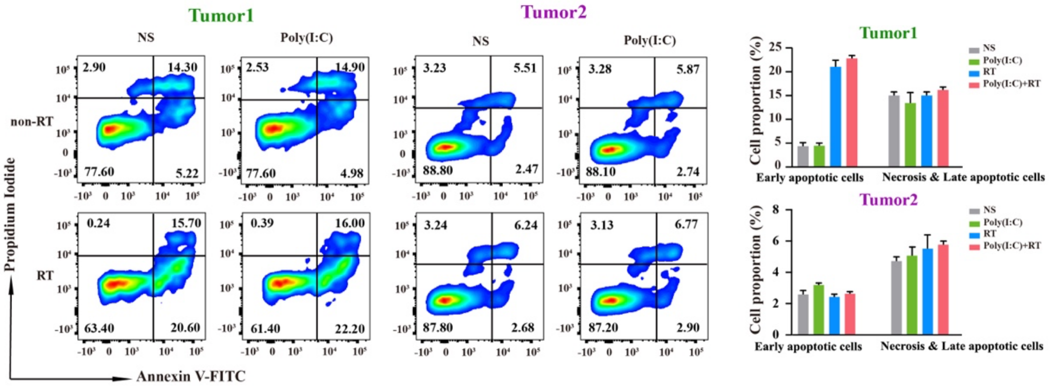

Appendix Figure S1 Effect of Poly(I:C) on tumor cell apoptosis of irradiated HCC mice by Annexin V/PI staining.

Representative dot plots (smooth) of Annexin V/PI staining of Tumor1 and Tumor2 in different treated groups. Annexin V^-/PI^- cells were survival cells, Annexin V^+/PI^- cells were defined as early apoptotic cells, Annexin V^+/PI^+ cells represented necrosis cells and late apoptotic cells. The results of three replicates were shown in statistical bar charts (means±SEM), and the differences among the groups were analyzed by ANOVA.

Appendix Figure S2

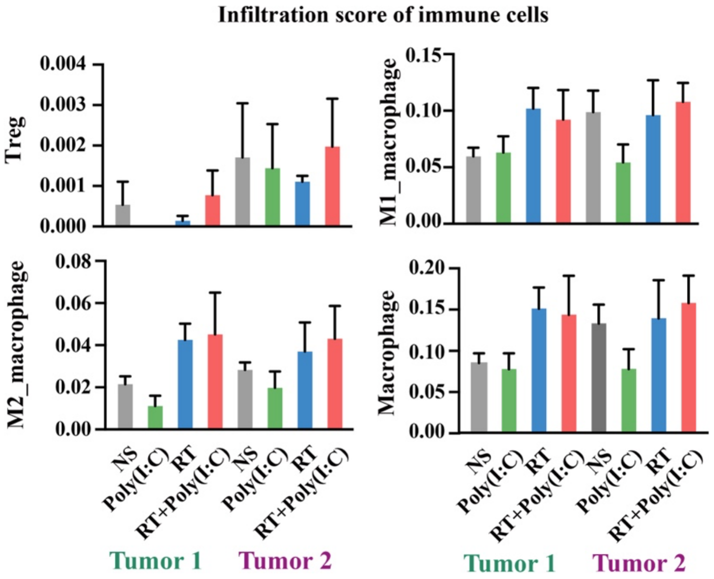

Appendix Figure S2 Infiltration score of Treg and macrophage of both Tumor1 and Tumor2 in different treated groups.

Infiltration score of Treg, macrophage (total, M1, M2) in tumor tissues according to RNA sequencing data analyzed by ImmuCellAI. The dada are shown as

mean±SEM(error bar), and ANOVA was performed to analyze the differences among groups.

Appendix Figure S3

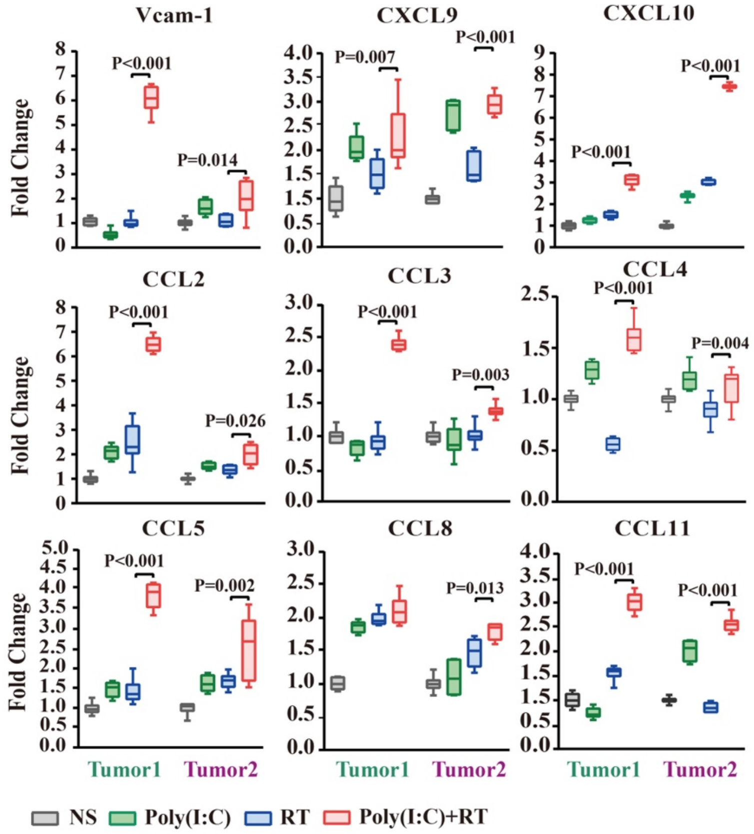

Appendix Figure S3 The expression of VCAM-1 and multiple chemokines in intratumoral vascular endothelium analyzed by qRT-PCR.

The relative expression level of mice in NS group was normalized to 1. The data represent the means±SEM(error bar) of at least three independent experiments, and ANOVA was performed to analyze the differences among groups.

Appendix Figure S4

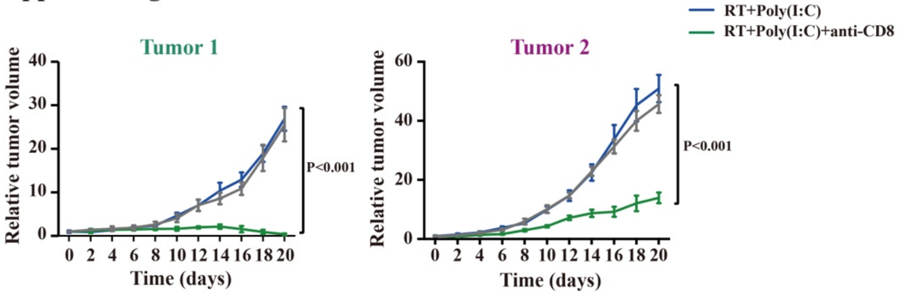

Appendix Figure S4 Poly(I:C) enhanced abscopal effect of radiotherapy depended on CD8$^+$ T cells in BALB/c mouse model with bilateral subcutaneous HCC.

Tumor growth curves of directly irradiated tumor (Tumor1) and distant tumor (Tumor2) in different treated groups (n=5 mice for each group) as indicated. Results are shown as mean±SEM(error bar), and ANOVA was performed to analyze the differences among groups.

Appendix Figure S5

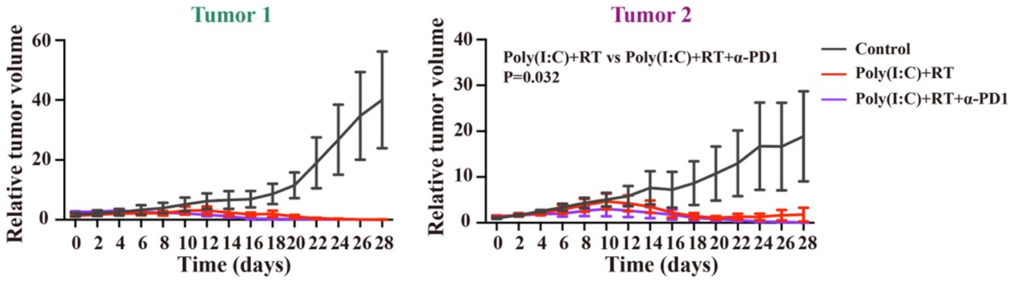

Appendix Figure S5 Effects of α-PD1 on tumor ferroptosis and tumor control induced by poly(I:C) plus RT in HCC mouse model.

Tumor growth curves of directly irradiated tumor (Tumor1) and distant tumor (Tumor2) in different treated groups (n=5 mice for each group) as indicated. Results are shown as mean±SEM(error bar), and ANOVA was performed to analyze the differences among groups.
