# Supplementary figures and images for: TLR3 activation enhances abscopal effect of radiotherapy in HCC by promoting tumor ferroptosis
Source: EMBO Mol Med. 2024 Apr 26;16(5):8. doi: 10.1038/s44321-024-00068-4 (PMC11098818; doi:10.1038/s44321-024-00068-4)

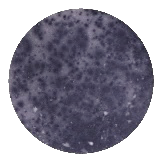

Supplement: Supplementary file 12 — Source data Fig. 4 [file 44321_2024_68_MOESM12_ESM.zip › Figure 4/Fig4A/ELISpot_images/Positive control_polyIC_1.tif]

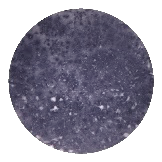

Supplement: Supplementary file 12 — Source data Fig. 4 [file 44321_2024_68_MOESM12_ESM.zip › Figure 4/Fig4A/ELISpot_images/Positive control_polyIC_2.tif]

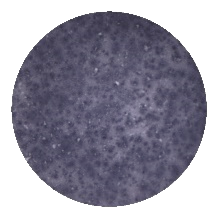

Supplement: Supplementary file 12 — Source data Fig. 4 [file 44321_2024_68_MOESM12_ESM.zip › Figure 4/Fig4A/ELISpot_images/Positive control_polyIC_3.tif]

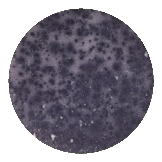

Supplement: Supplementary file 12 — Source data Fig. 4 [file 44321_2024_68_MOESM12_ESM.zip › Figure 4/Fig4A/ELISpot_images/Positive control_RT_1.tif]

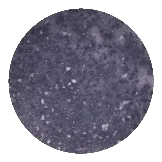

Supplement: Supplementary file 12 — Source data Fig. 4 [file 44321_2024_68_MOESM12_ESM.zip › Figure 4/Fig4A/ELISpot_images/Positive control_NS_2.tif]

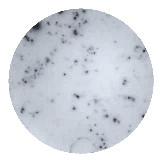

Supplement: Supplementary file 12 — Source data Fig. 4 [file 44321_2024_68_MOESM12_ESM.zip › Figure 4/Fig4A/ELISpot_images/Negtive control_polyIC+RT_1.tif]

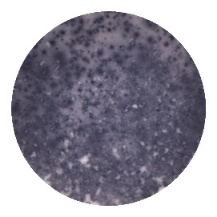

Supplement: Supplementary file 12 — Source data Fig. 4 [file 44321_2024_68_MOESM12_ESM.zip › Figure 4/Fig4A/ELISpot_images/Positive control_NS_3.tif]

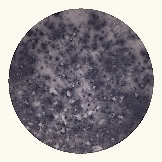

Supplement: Supplementary file 12 — Source data Fig. 4 [file 44321_2024_68_MOESM12_ESM.zip › Figure 4/Fig4A/ELISpot_images/Positive control_NS_1.tif]

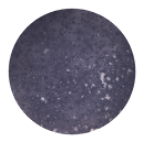

Supplement: Supplementary file 12 — Source data Fig. 4 [file 44321_2024_68_MOESM12_ESM.zip › Figure 4/Fig4A/ELISpot_images/Positive control_RT_2.tif]

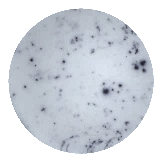

Supplement: Supplementary file 12 — Source data Fig. 4 [file 44321_2024_68_MOESM12_ESM.zip › Figure 4/Fig4A/ELISpot_images/Negtive control_polyIC+RT_2.tif]

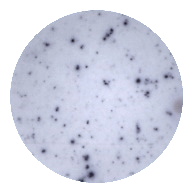

Supplement: Supplementary file 12 — Source data Fig. 4 [file 44321_2024_68_MOESM12_ESM.zip › Figure 4/Fig4A/ELISpot_images/Negtive control_polyIC+RT_3.tif]

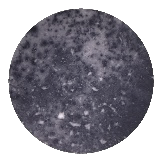

Supplement: Supplementary file 12 — Source data Fig. 4 [file 44321_2024_68_MOESM12_ESM.zip › Figure 4/Fig4A/ELISpot_images/Positive control_RT_3.tif]

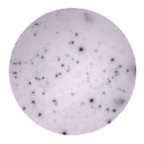

Supplement: Supplementary file 12 — Source data Fig. 4 [file 44321_2024_68_MOESM12_ESM.zip › Figure 4/Fig4A/ELISpot_images/Negtive control_polyIC_3.tiff]

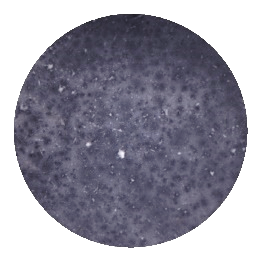

Supplement: Supplementary file 12 — Source data Fig. 4 [file 44321_2024_68_MOESM12_ESM.zip › Figure 4/Fig4A/ELISpot_images/Positive control_polyIC+RT_1.tif]

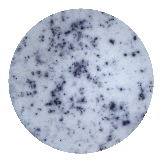

Supplement: Supplementary file 12 — Source data Fig. 4 [file 44321_2024_68_MOESM12_ESM.zip › Figure 4/Fig4A/ELISpot_images/neoantigen-peptide pool_polyIC+RT_1.tif]

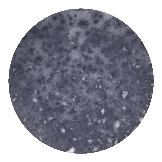

Supplement: Supplementary file 12 — Source data Fig. 4 [file 44321_2024_68_MOESM12_ESM.zip › Figure 4/Fig4A/ELISpot_images/Positive control_polyIC+RT_2.tif]

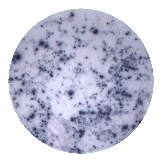

Supplement: Supplementary file 12 — Source data Fig. 4 [file 44321_2024_68_MOESM12_ESM.zip › Figure 4/Fig4A/ELISpot_images/neoantigen-peptide pool_polyIC+RT_2.tif]

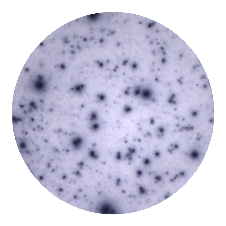

Supplement: Supplementary file 12 — Source data Fig. 4 [file 44321_2024_68_MOESM12_ESM.zip › Figure 4/Fig4A/ELISpot_images/neoantigen-peptide pool_polyIC+RT_3.tif]

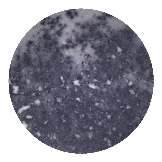

Supplement: Supplementary file 12 — Source data Fig. 4 [file 44321_2024_68_MOESM12_ESM.zip › Figure 4/Fig4A/ELISpot_images/Positive control_polyIC+RT_3.tif]

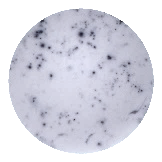

Supplement: Supplementary file 12 — Source data Fig. 4 [file 44321_2024_68_MOESM12_ESM.zip › Figure 4/Fig4A/ELISpot_images/neoantigen-peptide pool_RT_3.tif]

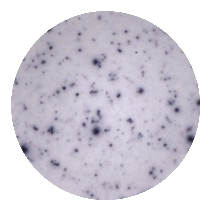

Supplement: Supplementary file 12 — Source data Fig. 4 [file 44321_2024_68_MOESM12_ESM.zip › Figure 4/Fig4A/ELISpot_images/neoantigen-peptide pool_RT_2.tif]

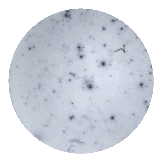

Supplement: Supplementary file 12 — Source data Fig. 4 [file 44321_2024_68_MOESM12_ESM.zip › Figure 4/Fig4A/ELISpot_images/neoantigen-peptide pool_NS_1.tif]

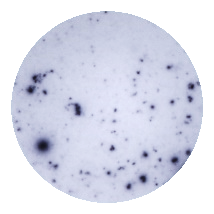

Supplement: Supplementary file 12 — Source data Fig. 4 [file 44321_2024_68_MOESM12_ESM.zip › Figure 4/Fig4A/ELISpot_images/neoantigen-peptide pool_NS_3.tif]

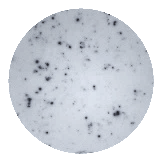

Supplement: Supplementary file 12 — Source data Fig. 4 [file 44321_2024_68_MOESM12_ESM.zip › Figure 4/Fig4A/ELISpot_images/neoantigen-peptide pool_NS_2.tif]

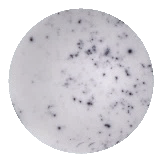

Supplement: Supplementary file 12 — Source data Fig. 4 [file 44321_2024_68_MOESM12_ESM.zip › Figure 4/Fig4A/ELISpot_images/neoantigen-peptide pool_RT_1.tif]

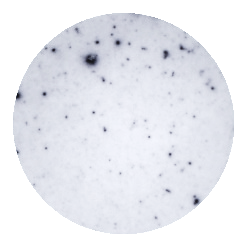

Supplement: Supplementary file 12 — Source data Fig. 4 [file 44321_2024_68_MOESM12_ESM.zip › Figure 4/Fig4A/ELISpot_images/Negtive control_polyIC_1.tif]

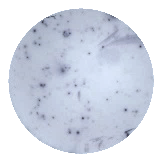

Supplement: Supplementary file 12 — Source data Fig. 4 [file 44321_2024_68_MOESM12_ESM.zip › Figure 4/Fig4A/ELISpot_images/Negtive control_polyIC_2.tif]

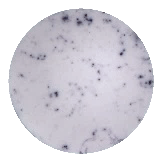

Supplement: Supplementary file 12 — Source data Fig. 4 [file 44321_2024_68_MOESM12_ESM.zip › Figure 4/Fig4A/ELISpot_images/neoantigen-peptide pool_polyIC_1.tif]

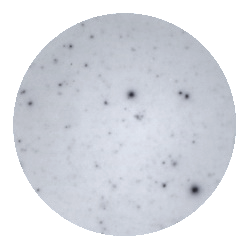

Supplement: Supplementary file 12 — Source data Fig. 4 [file 44321_2024_68_MOESM12_ESM.zip › Figure 4/Fig4A/ELISpot_images/Negtive control_NS_3.tif]

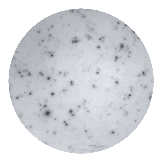

Supplement: Supplementary file 12 — Source data Fig. 4 [file 44321_2024_68_MOESM12_ESM.zip › Figure 4/Fig4A/ELISpot_images/Negtive control_NS_2.tif]

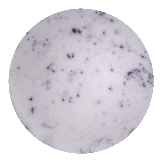

Supplement: Supplementary file 12 — Source data Fig. 4 [file 44321_2024_68_MOESM12_ESM.zip › Figure 4/Fig4A/ELISpot_images/Negtive control_RT_1.tif]

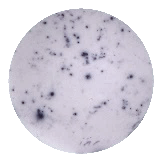

Supplement: Supplementary file 12 — Source data Fig. 4 [file 44321_2024_68_MOESM12_ESM.zip › Figure 4/Fig4A/ELISpot_images/neoantigen-peptide pool_polyIC_2.tif]

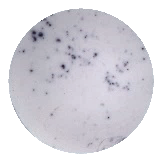

Supplement: Supplementary file 12 — Source data Fig. 4 [file 44321_2024_68_MOESM12_ESM.zip › Figure 4/Fig4A/ELISpot_images/Negtive control_RT_3.tif]

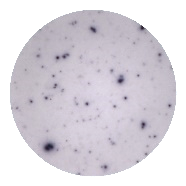

Supplement: Supplementary file 12 — Source data Fig. 4 [file 44321_2024_68_MOESM12_ESM.zip › Figure 4/Fig4A/ELISpot_images/Negtive control_RT_2.tif]

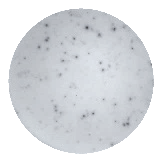

Supplement: Supplementary file 12 — Source data Fig. 4 [file 44321_2024_68_MOESM12_ESM.zip › Figure 4/Fig4A/ELISpot_images/Negtive control_NS_1.tif]

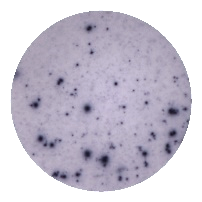

Supplement: Supplementary file 12 — Source data Fig. 4 [file 44321_2024_68_MOESM12_ESM.zip › Figure 4/Fig4A/ELISpot_images/neoantigen-peptide pool_polyIC_3.tif]

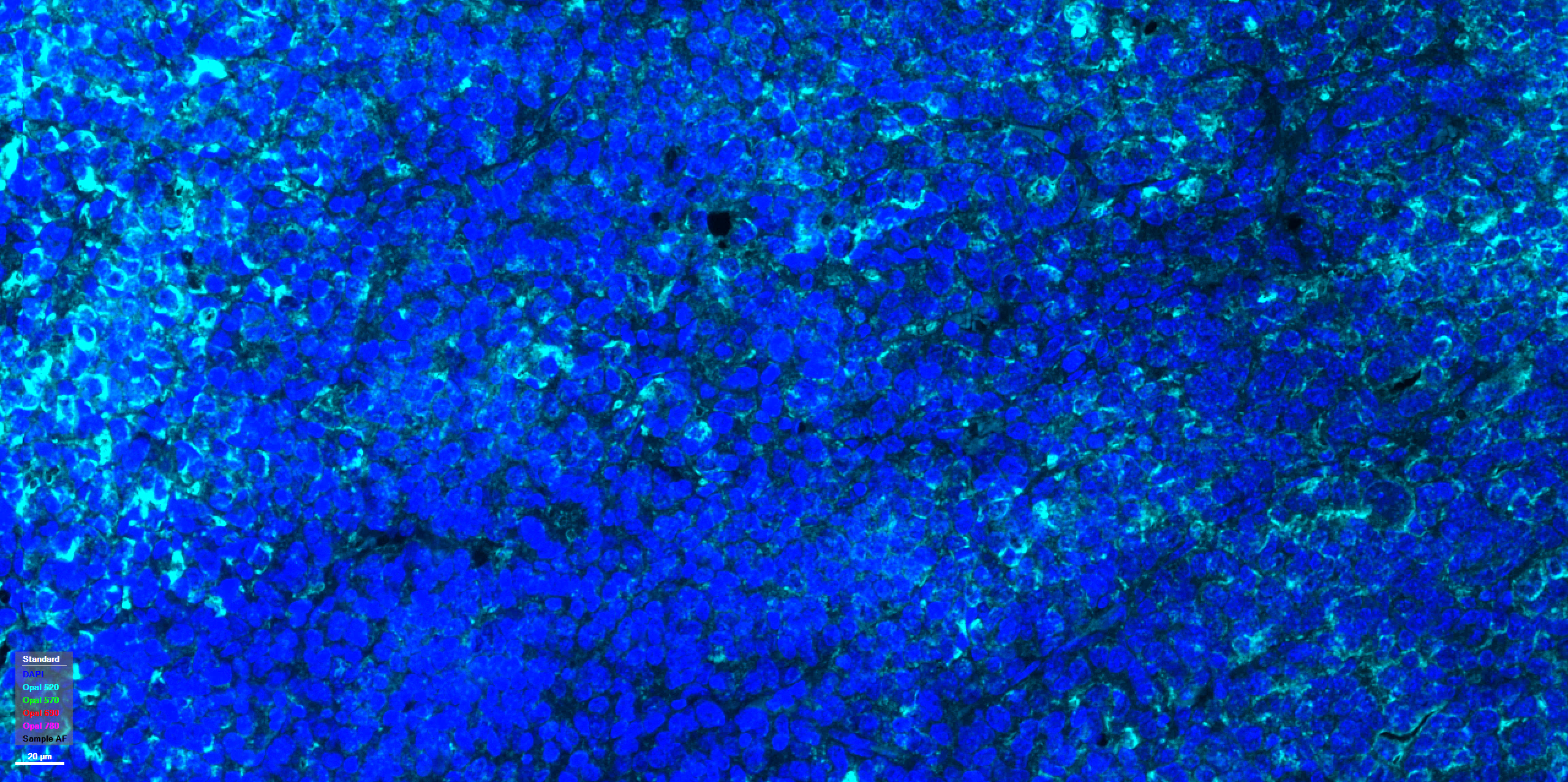

Supplement: Supplementary file 12 — Source data Fig. 4 [file 44321_2024_68_MOESM12_ESM.zip › Figure 4/Fig4C/mIHC_images_CD11c:CD45:CLEC9:IL12/RT_IL12.tiff]

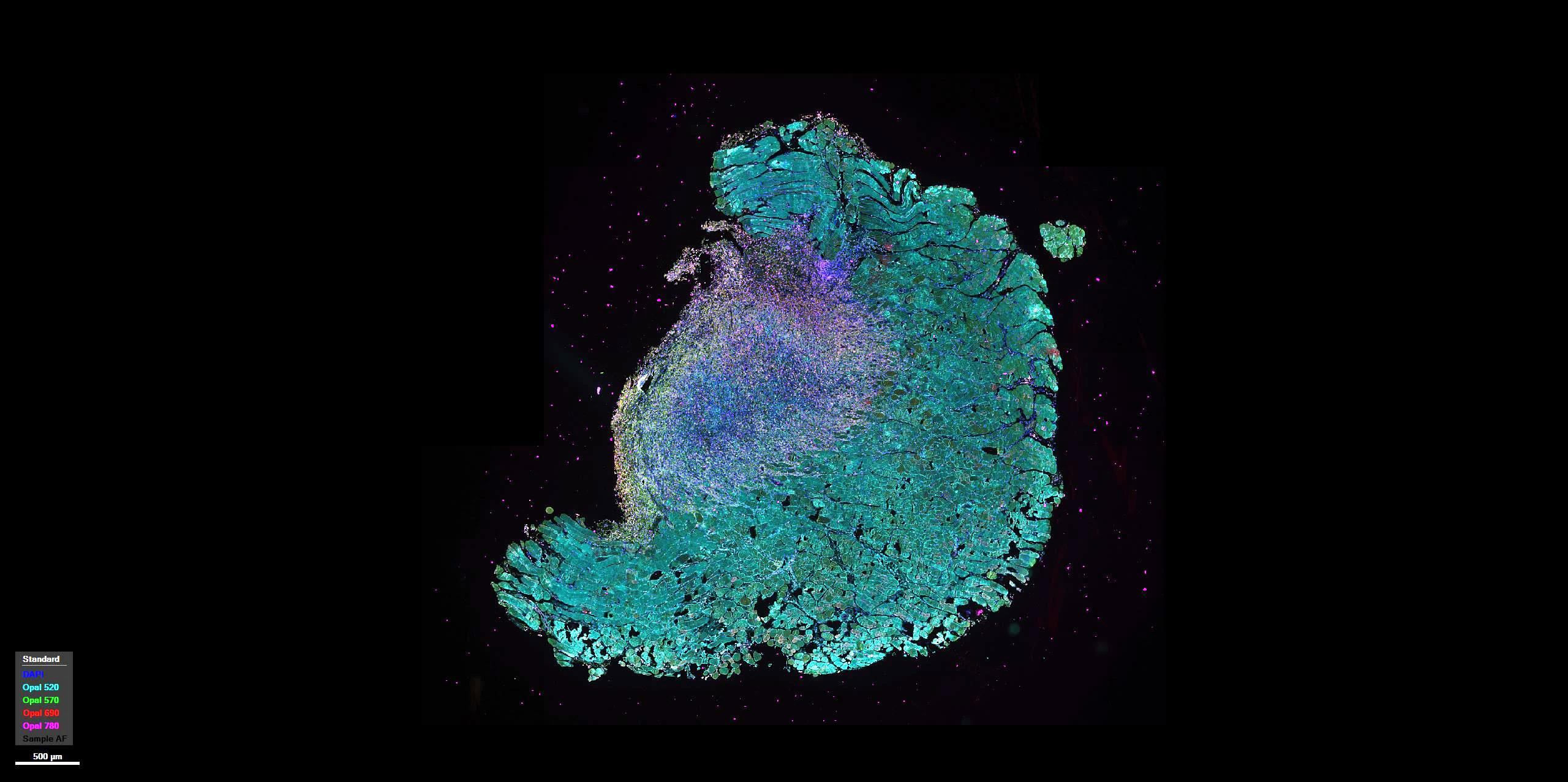

Supplement: Supplementary file 12 — Source data Fig. 4 [file 44321_2024_68_MOESM12_ESM.zip › Figure 4/Fig4C/mIHC_images_CD11c:CD45:CLEC9:IL12/polyIC+RT_overview.tiff]

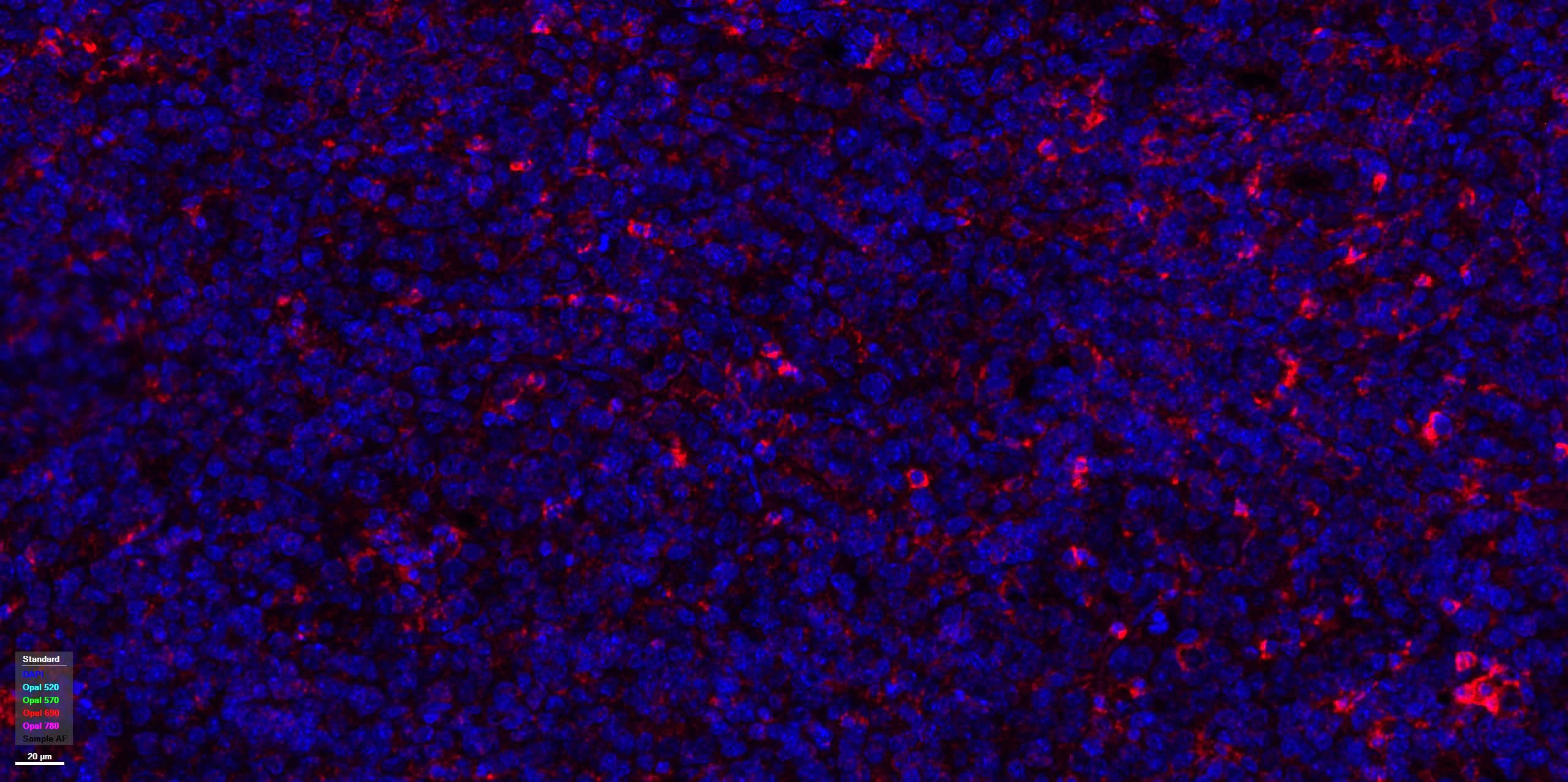

Supplement: Supplementary file 12 — Source data Fig. 4 [file 44321_2024_68_MOESM12_ESM.zip › Figure 4/Fig4C/mIHC_images_CD11c:CD45:CLEC9:IL12/RT_CD45.tiff]

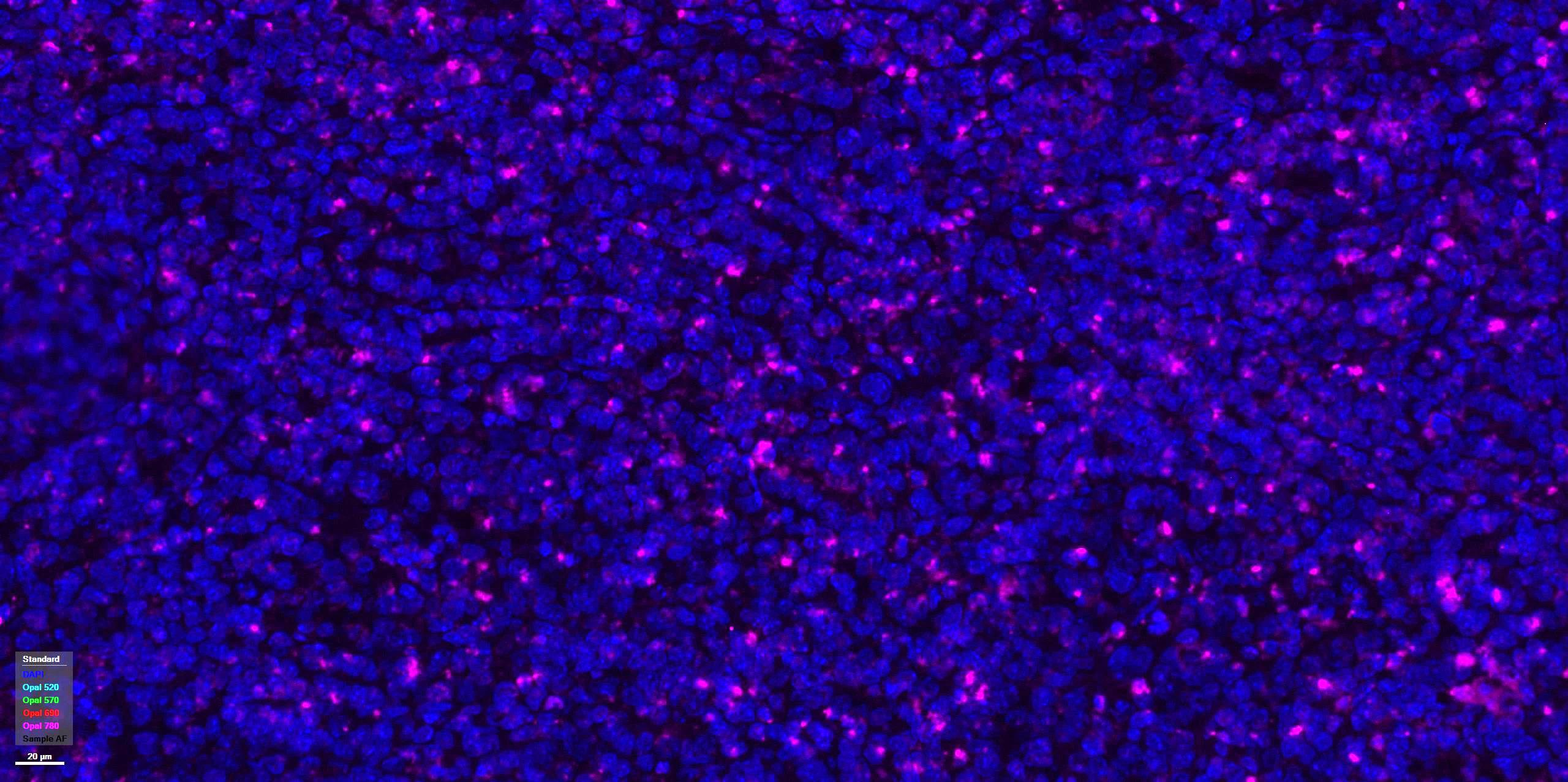

Supplement: Supplementary file 12 — Source data Fig. 4 [file 44321_2024_68_MOESM12_ESM.zip › Figure 4/Fig4C/mIHC_images_CD11c:CD45:CLEC9:IL12/RT_CLEC9.tiff]

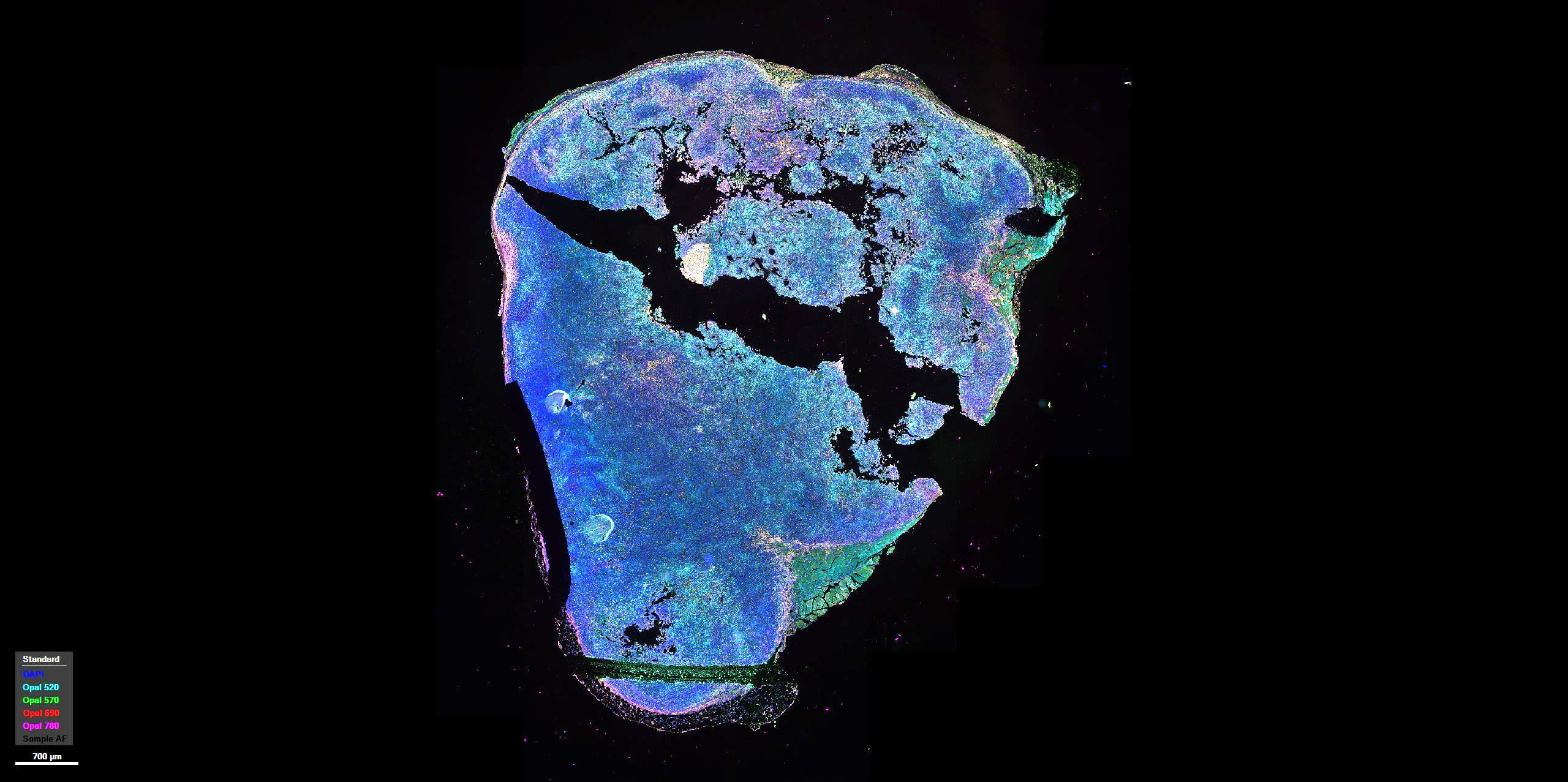

Supplement: Supplementary file 12 — Source data Fig. 4 [file 44321_2024_68_MOESM12_ESM.zip › Figure 4/Fig4C/mIHC_images_CD11c:CD45:CLEC9:IL12/RT_overview.tiff]

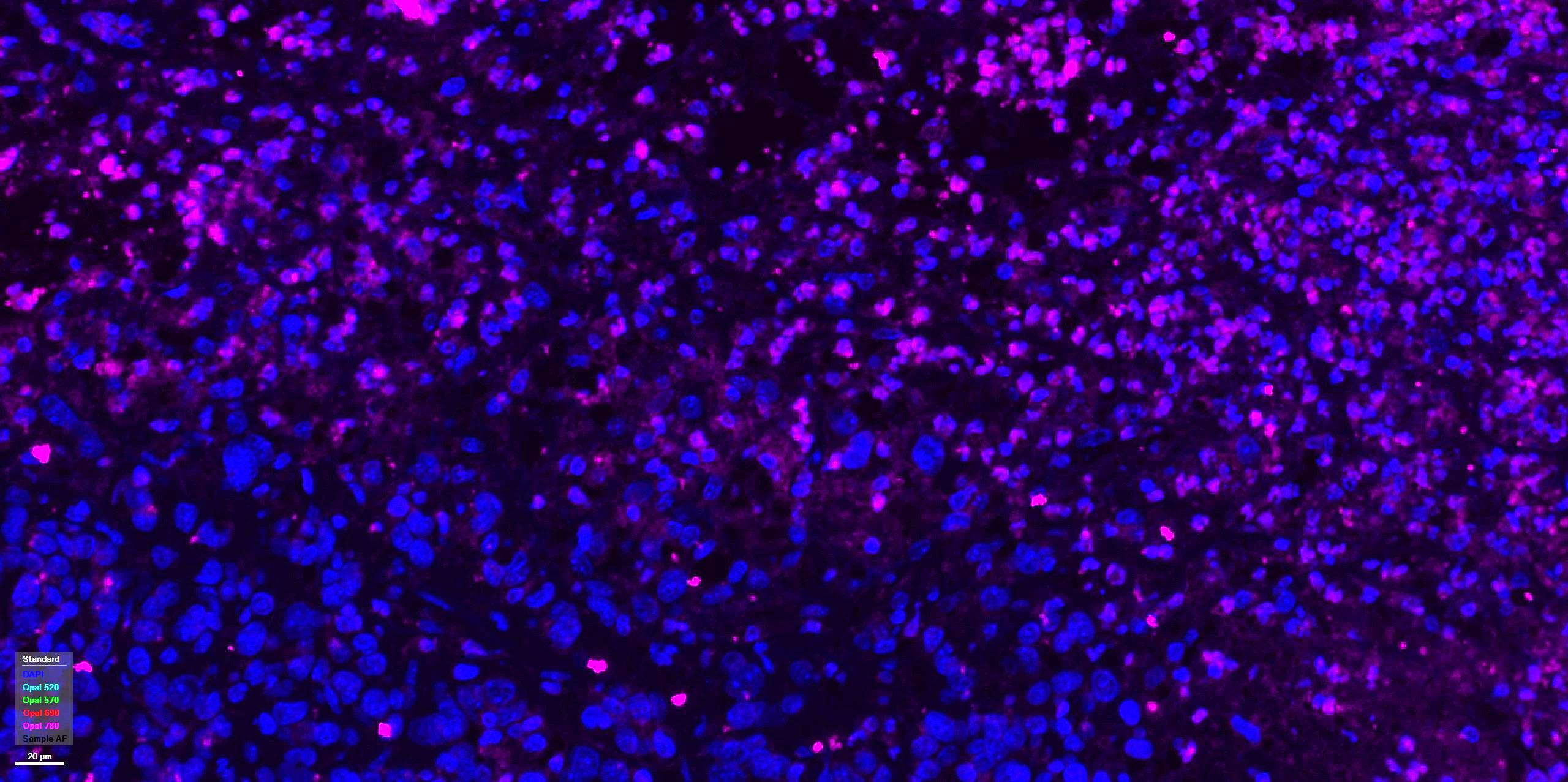

Supplement: Supplementary file 12 — Source data Fig. 4 [file 44321_2024_68_MOESM12_ESM.zip › Figure 4/Fig4C/mIHC_images_CD11c:CD45:CLEC9:IL12/polyIC+RT_CLEC9.tiff]

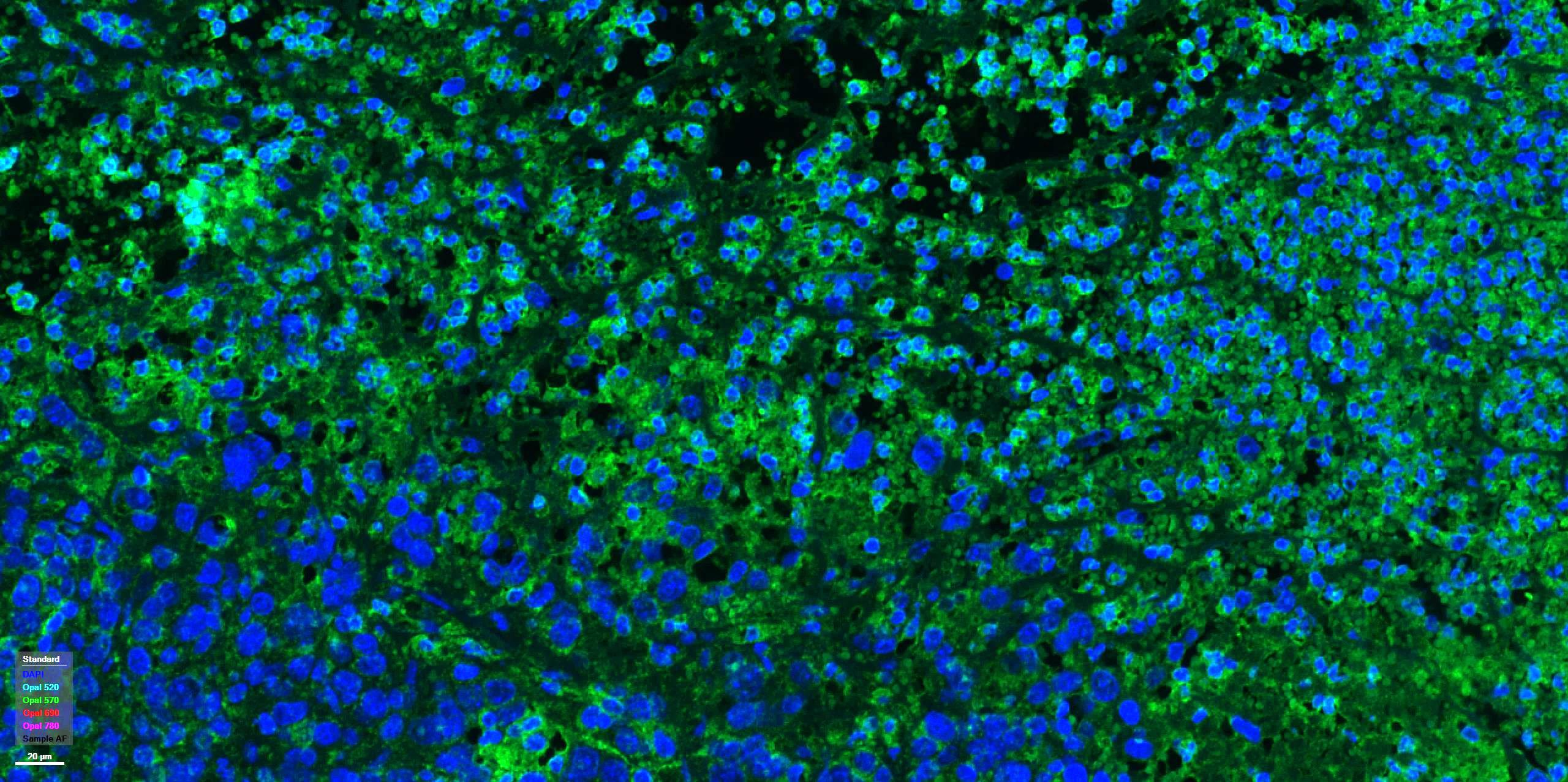

Supplement: Supplementary file 12 — Source data Fig. 4 [file 44321_2024_68_MOESM12_ESM.zip › Figure 4/Fig4C/mIHC_images_CD11c:CD45:CLEC9:IL12/polyIC+RT_CD11c.tiff]

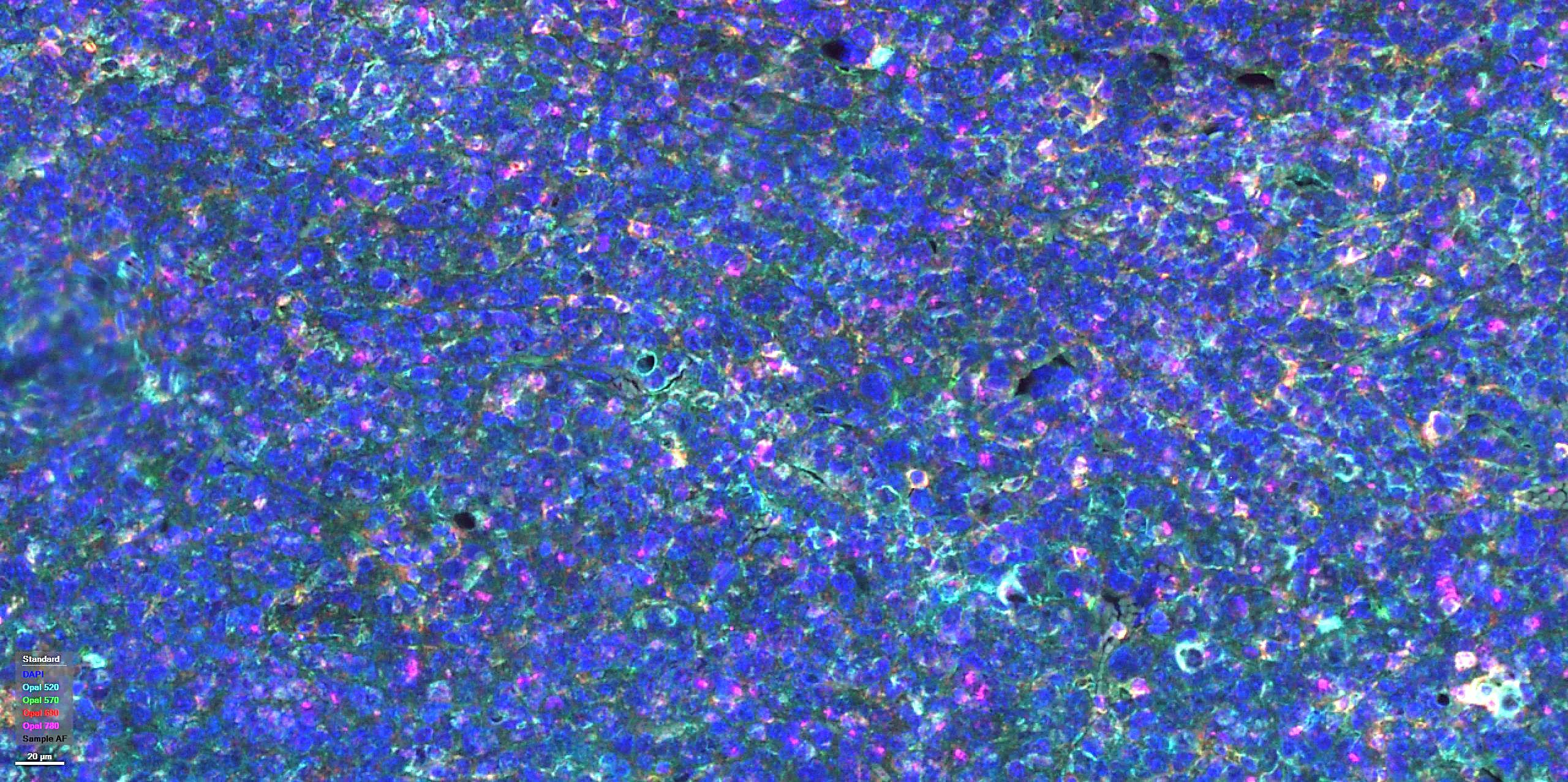

Supplement: Supplementary file 12 — Source data Fig. 4 [file 44321_2024_68_MOESM12_ESM.zip › Figure 4/Fig4C/mIHC_images_CD11c:CD45:CLEC9:IL12/RT_all.tiff]

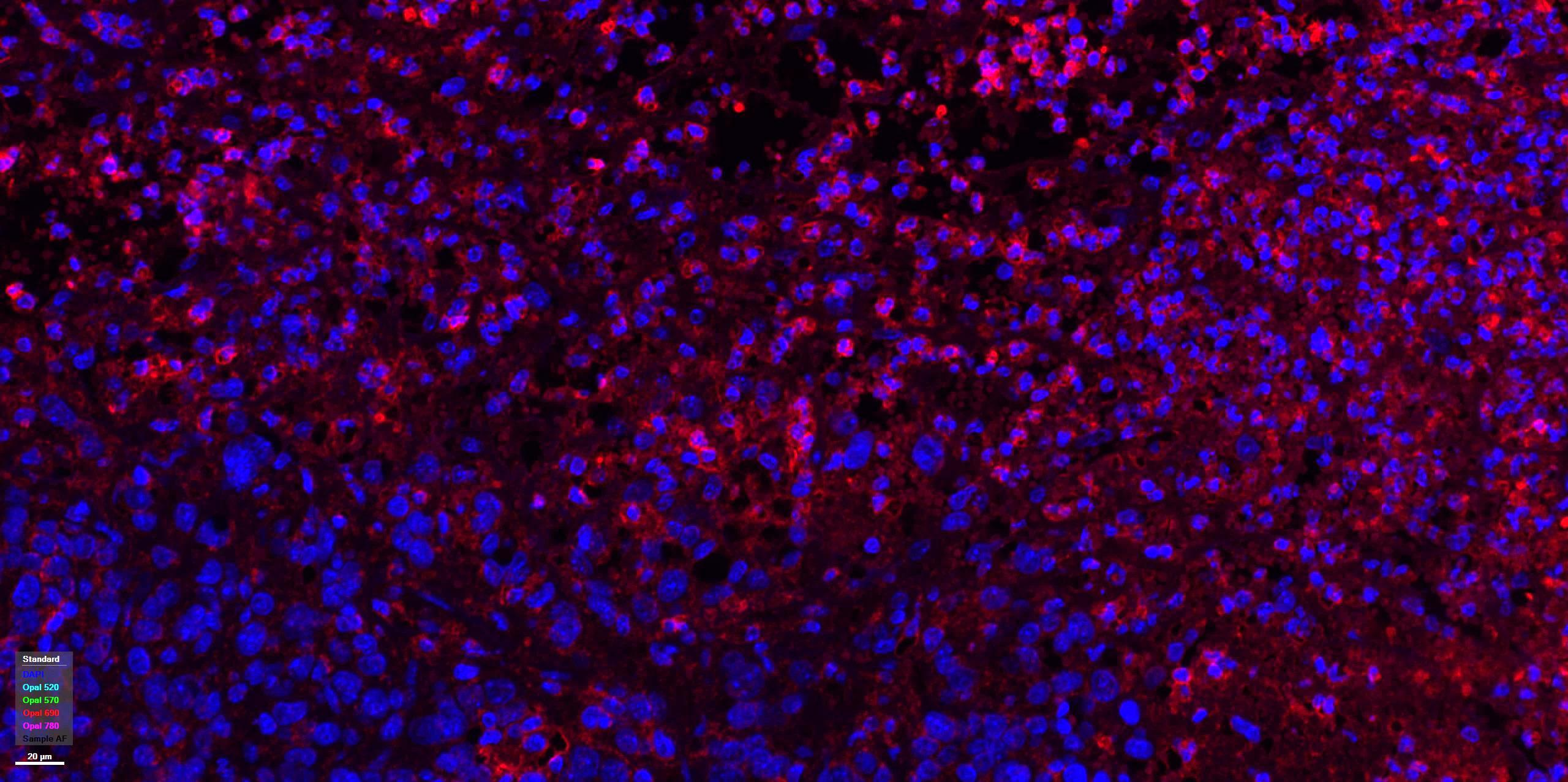

Supplement: Supplementary file 12 — Source data Fig. 4 [file 44321_2024_68_MOESM12_ESM.zip › Figure 4/Fig4C/mIHC_images_CD11c:CD45:CLEC9:IL12/polyIC+RT_CD45.tiff]

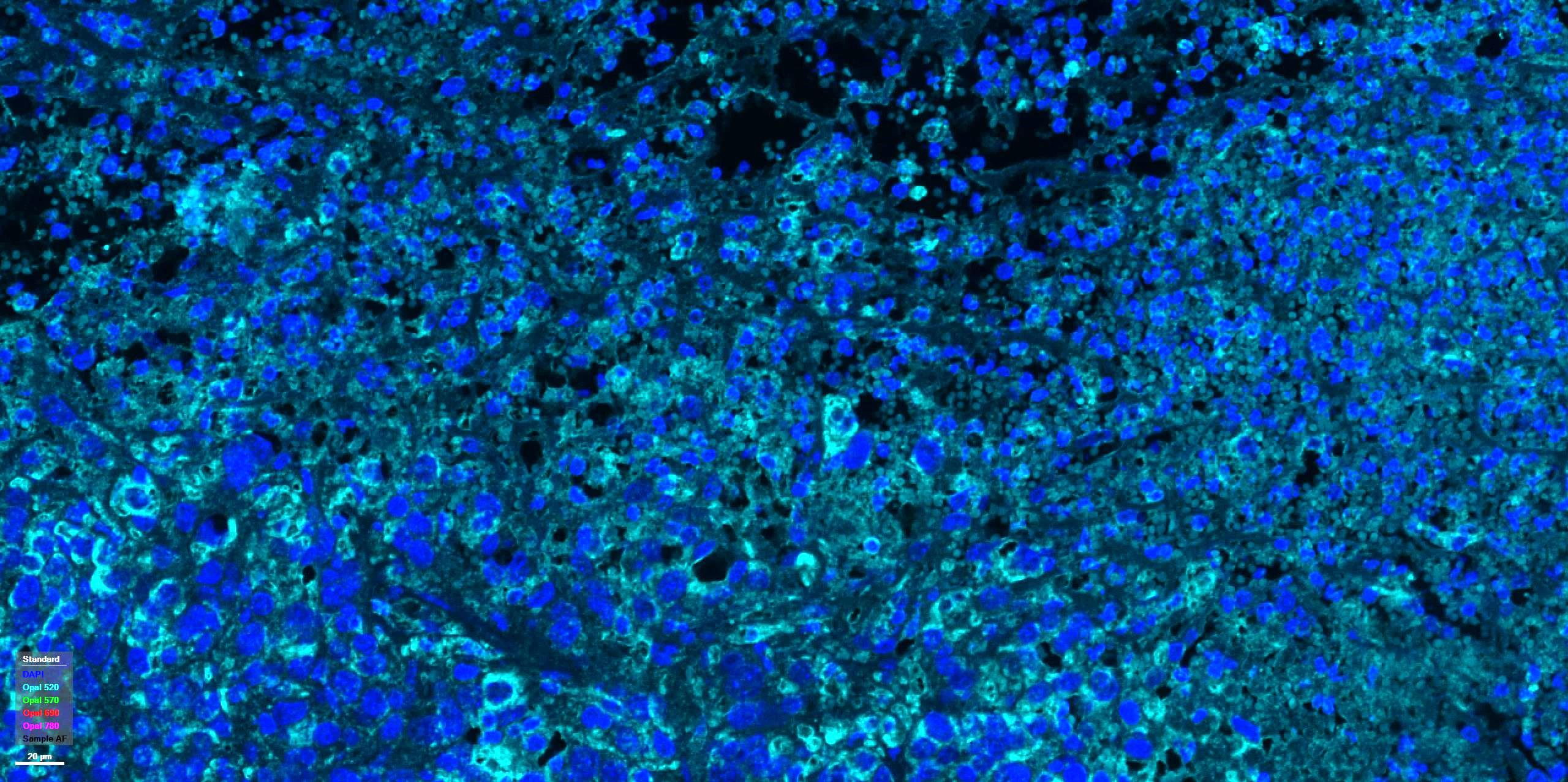

Supplement: Supplementary file 12 — Source data Fig. 4 [file 44321_2024_68_MOESM12_ESM.zip › Figure 4/Fig4C/mIHC_images_CD11c:CD45:CLEC9:IL12/polyIC+RT_IL12.tiff]

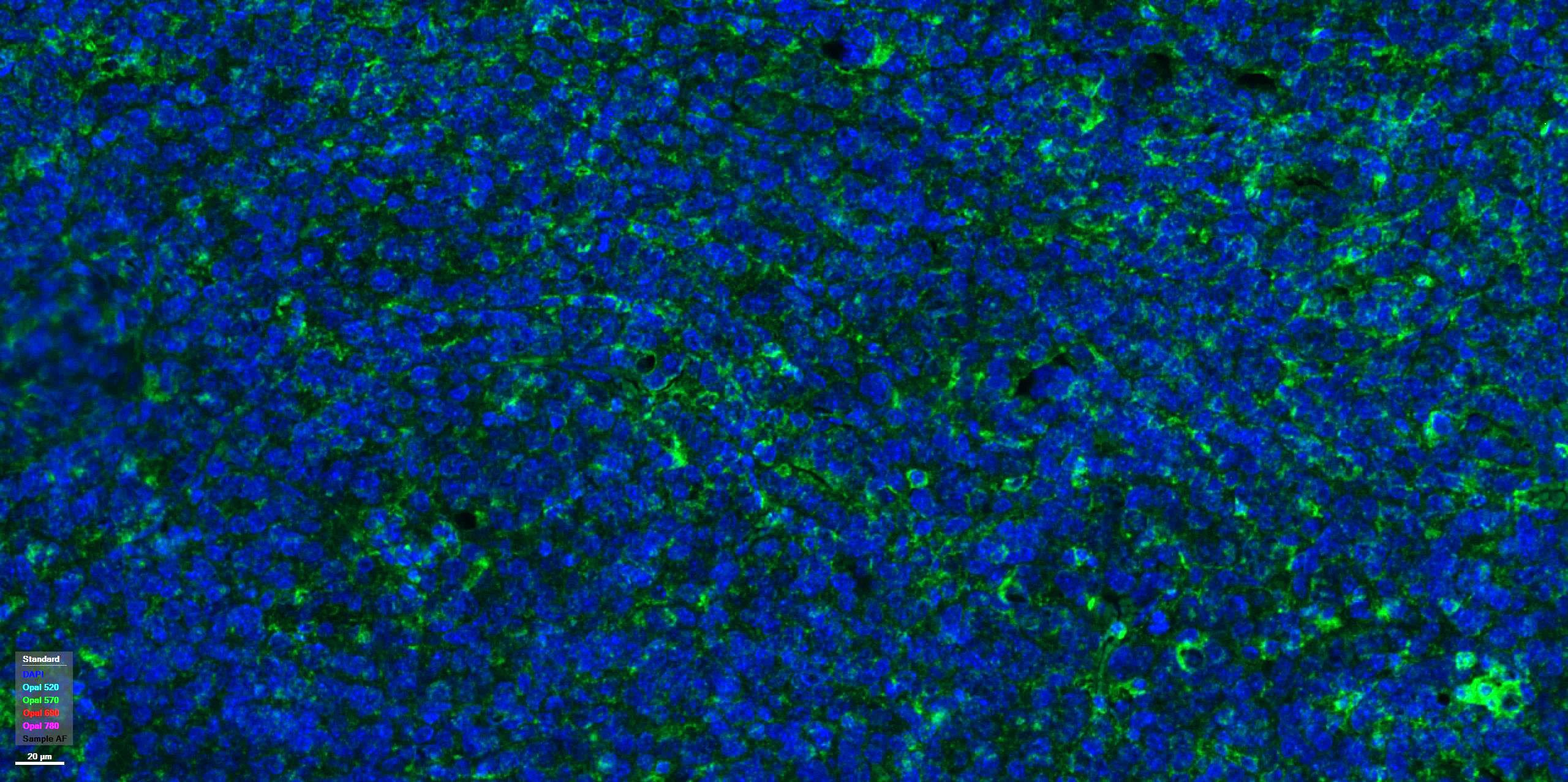

Supplement: Supplementary file 12 — Source data Fig. 4 [file 44321_2024_68_MOESM12_ESM.zip › Figure 4/Fig4C/mIHC_images_CD11c:CD45:CLEC9:IL12/RT_CD11c.tiff]

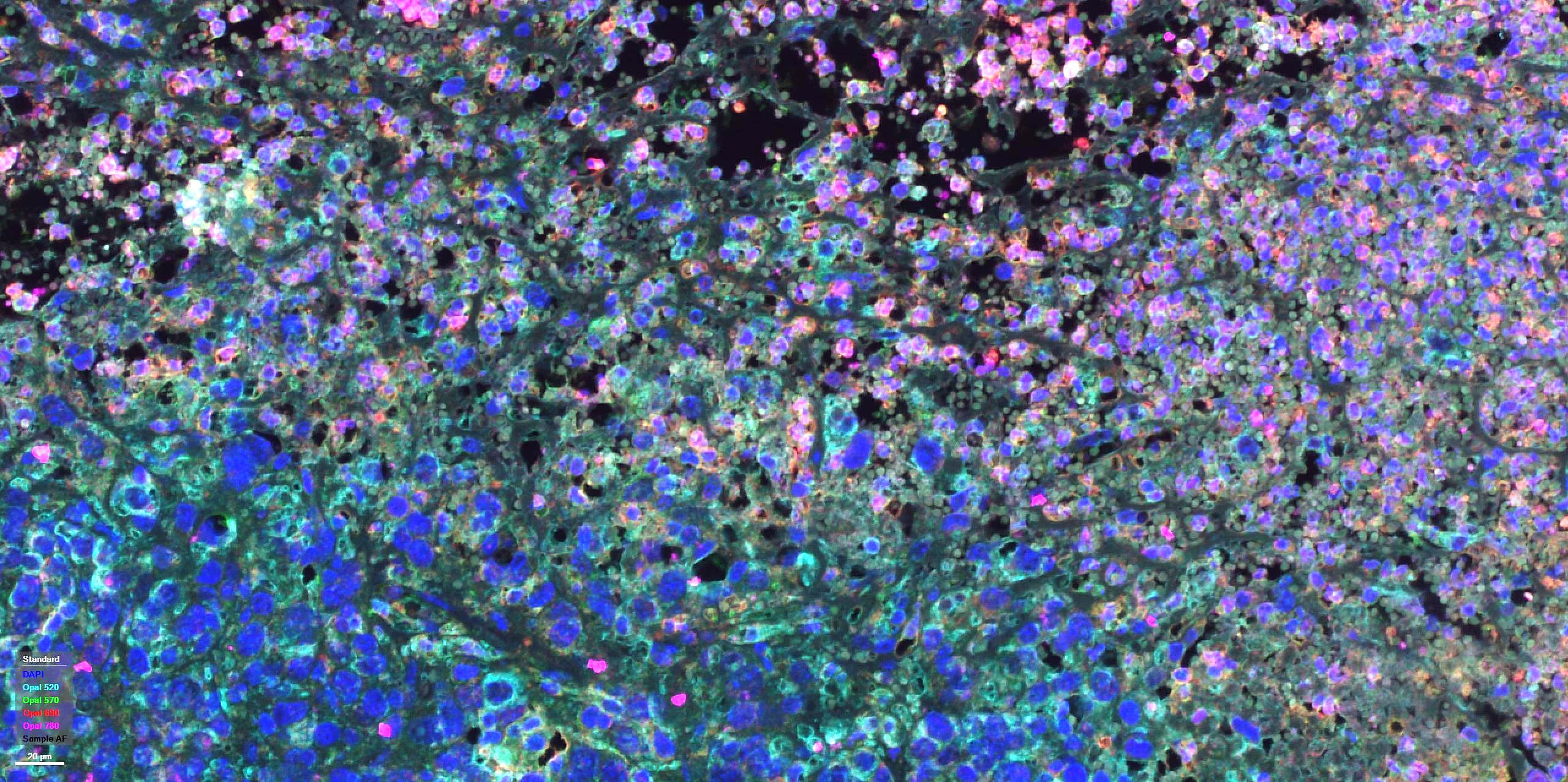

Supplement: Supplementary file 12 — Source data Fig. 4 [file 44321_2024_68_MOESM12_ESM.zip › Figure 4/Fig4C/mIHC_images_CD11c:CD45:CLEC9:IL12/polyIC+RT_all.tiff]

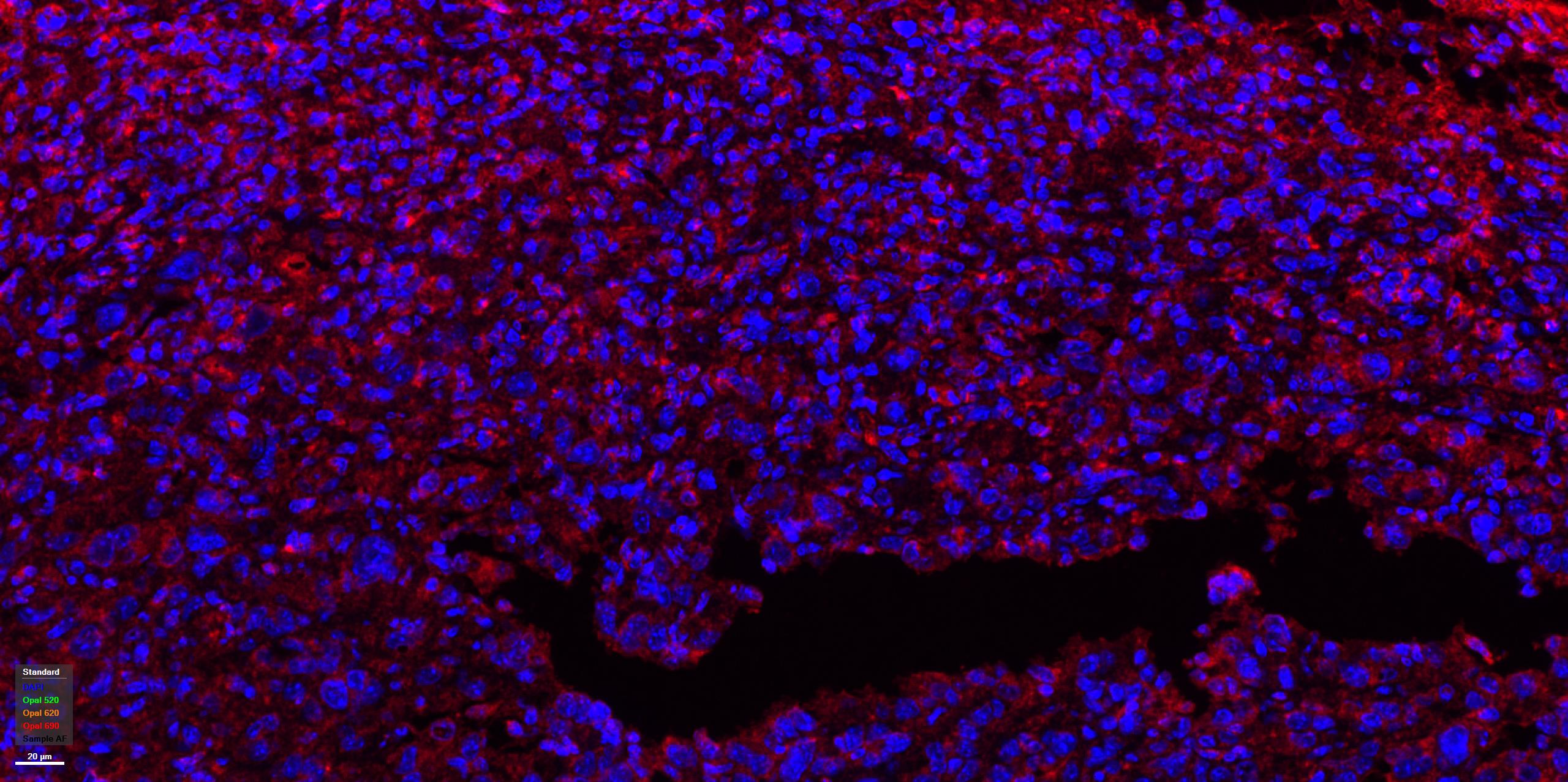

Supplement: Supplementary file 13 — Source data Fig. 5 [file 44321_2024_68_MOESM13_ESM.zip › Figure 5/Fig5B/mIHC_images_CD8:GzmB:TLR3/Tumor1_polyIC+RT_TLR3.tiff]

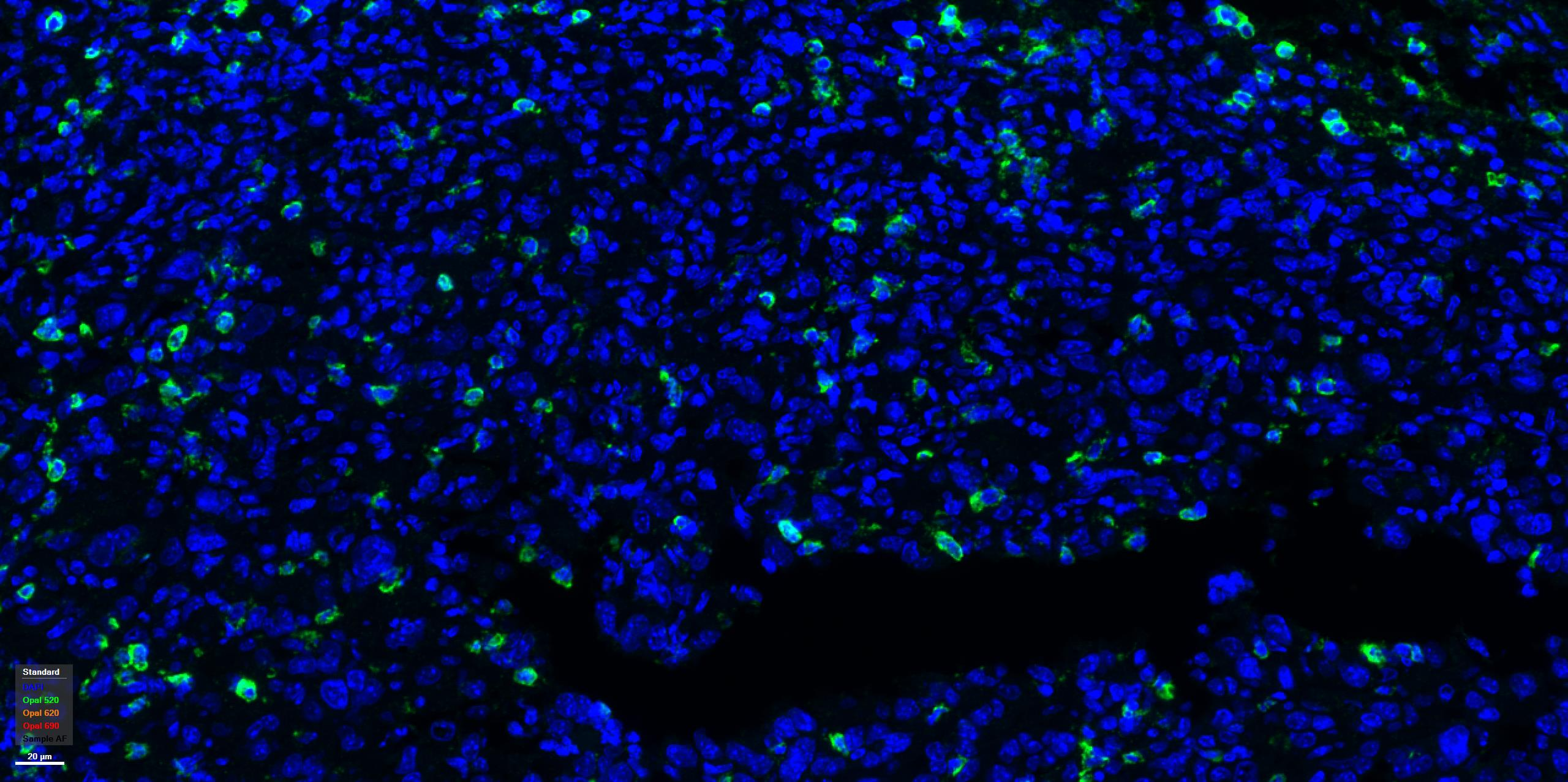

Supplement: Supplementary file 13 — Source data Fig. 5 [file 44321_2024_68_MOESM13_ESM.zip › Figure 5/Fig5B/mIHC_images_CD8:GzmB:TLR3/Tumor1_polyIC+RT_CD8.tiff]

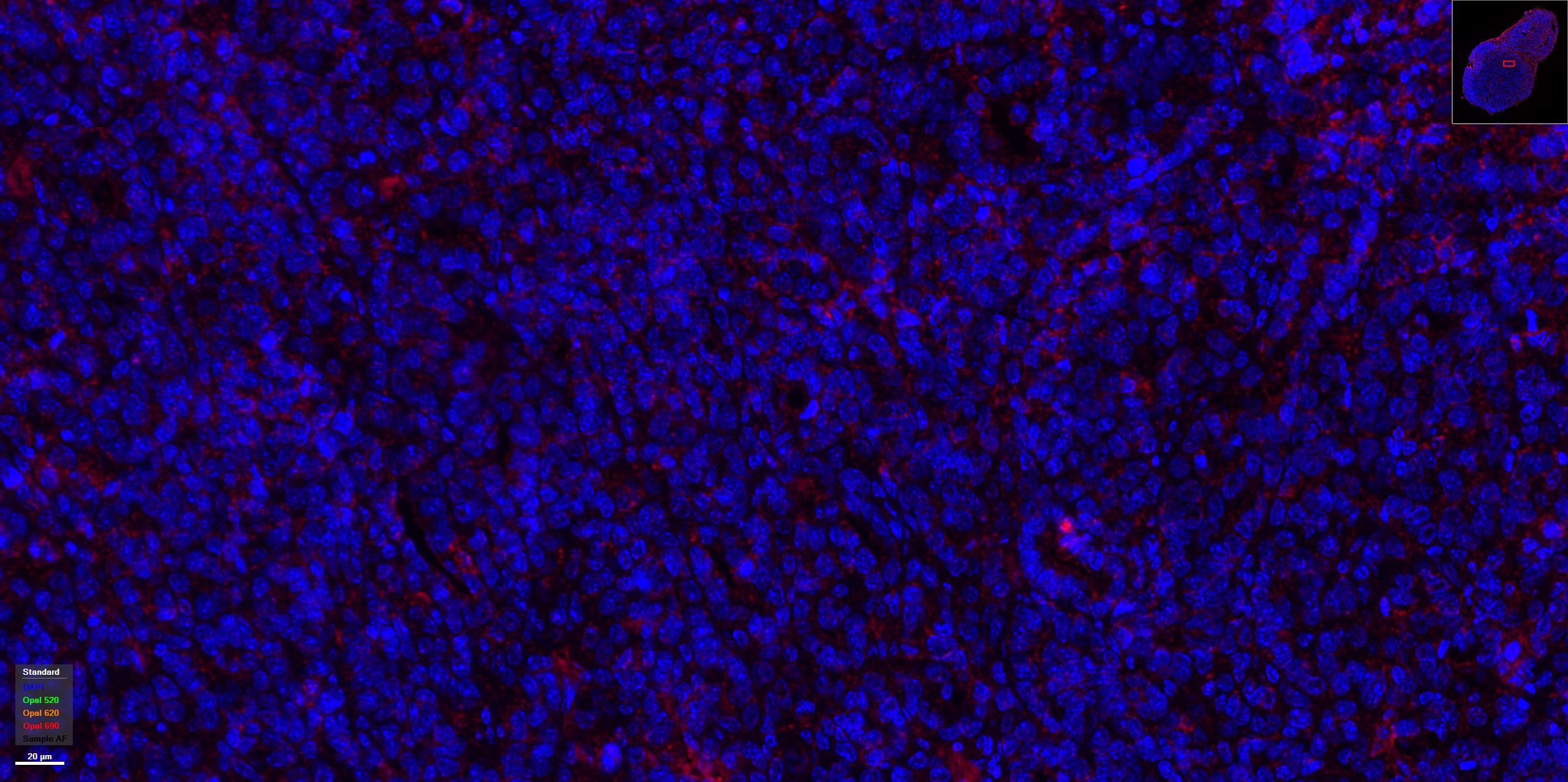

Supplement: Supplementary file 13 — Source data Fig. 5 [file 44321_2024_68_MOESM13_ESM.zip › Figure 5/Fig5B/mIHC_images_CD8:GzmB:TLR3/Tumor1_RT_TLR3.tiff]

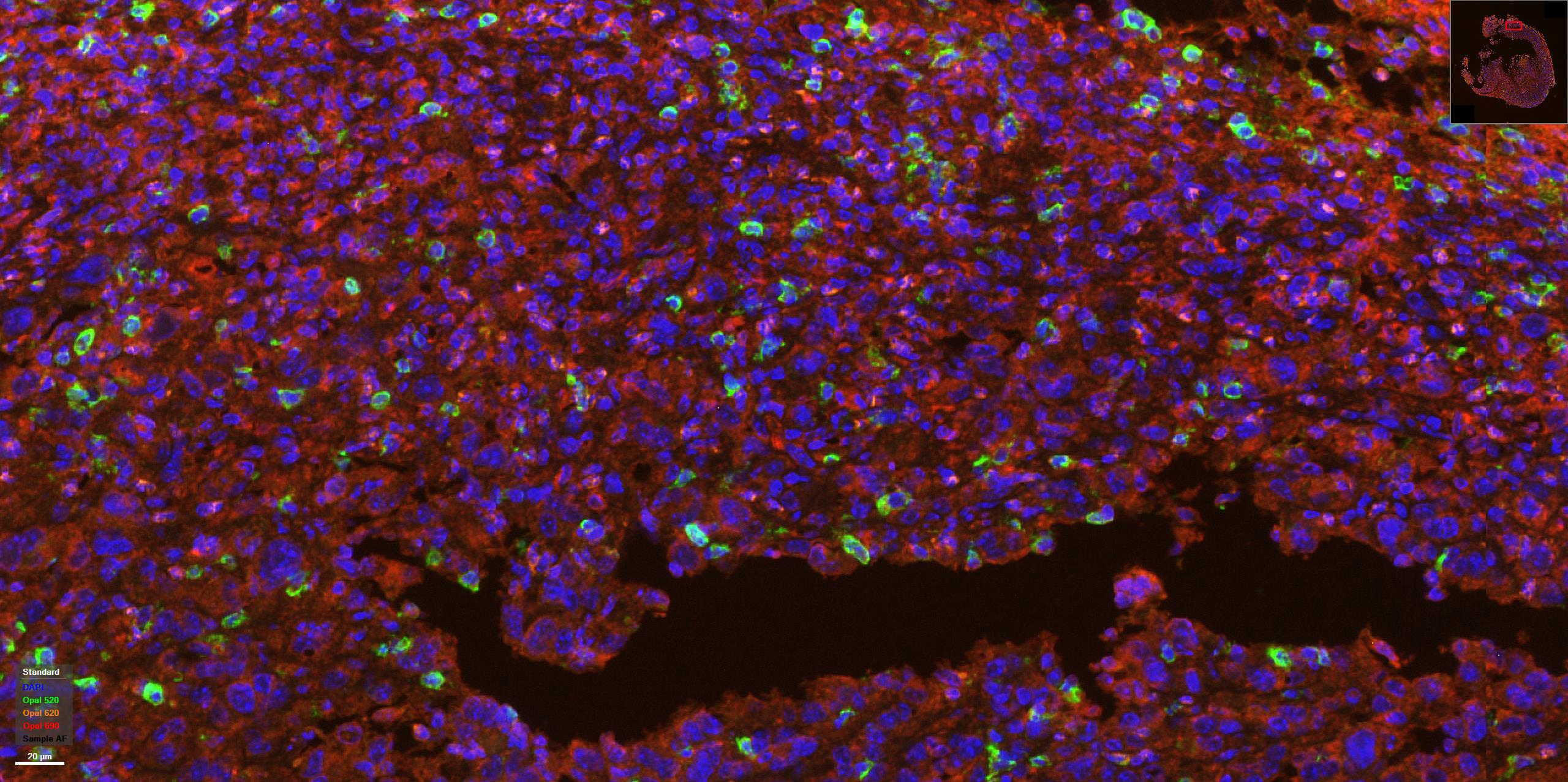

Supplement: Supplementary file 13 — Source data Fig. 5 [file 44321_2024_68_MOESM13_ESM.zip › Figure 5/Fig5B/mIHC_images_CD8:GzmB:TLR3/Tumor1_polyIC+RT_all.tiff]

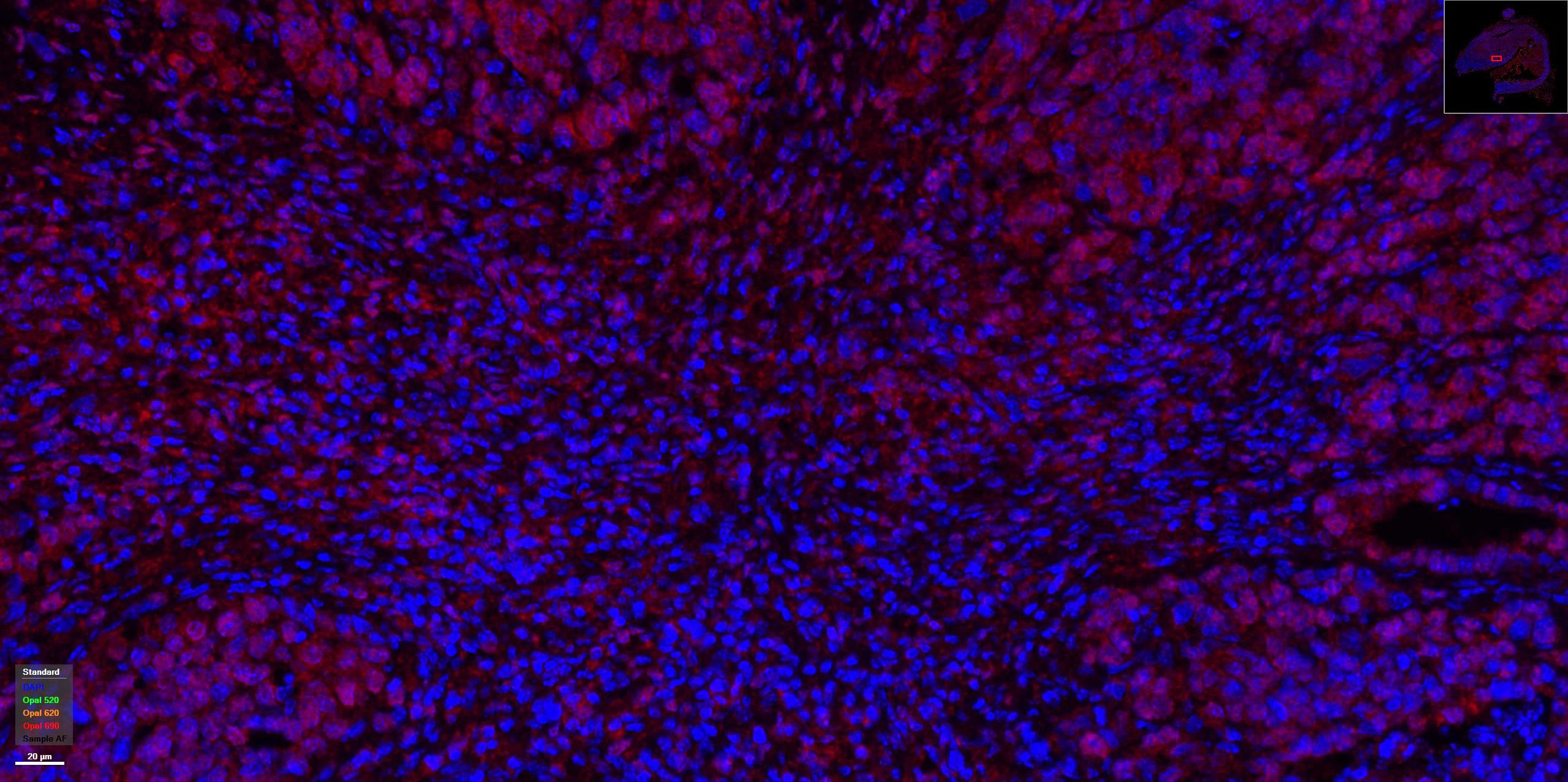

Supplement: Supplementary file 13 — Source data Fig. 5 [file 44321_2024_68_MOESM13_ESM.zip › Figure 5/Fig5B/mIHC_images_CD8:GzmB:TLR3/Tumor2_polyIC+RT_TLR3.tiff]

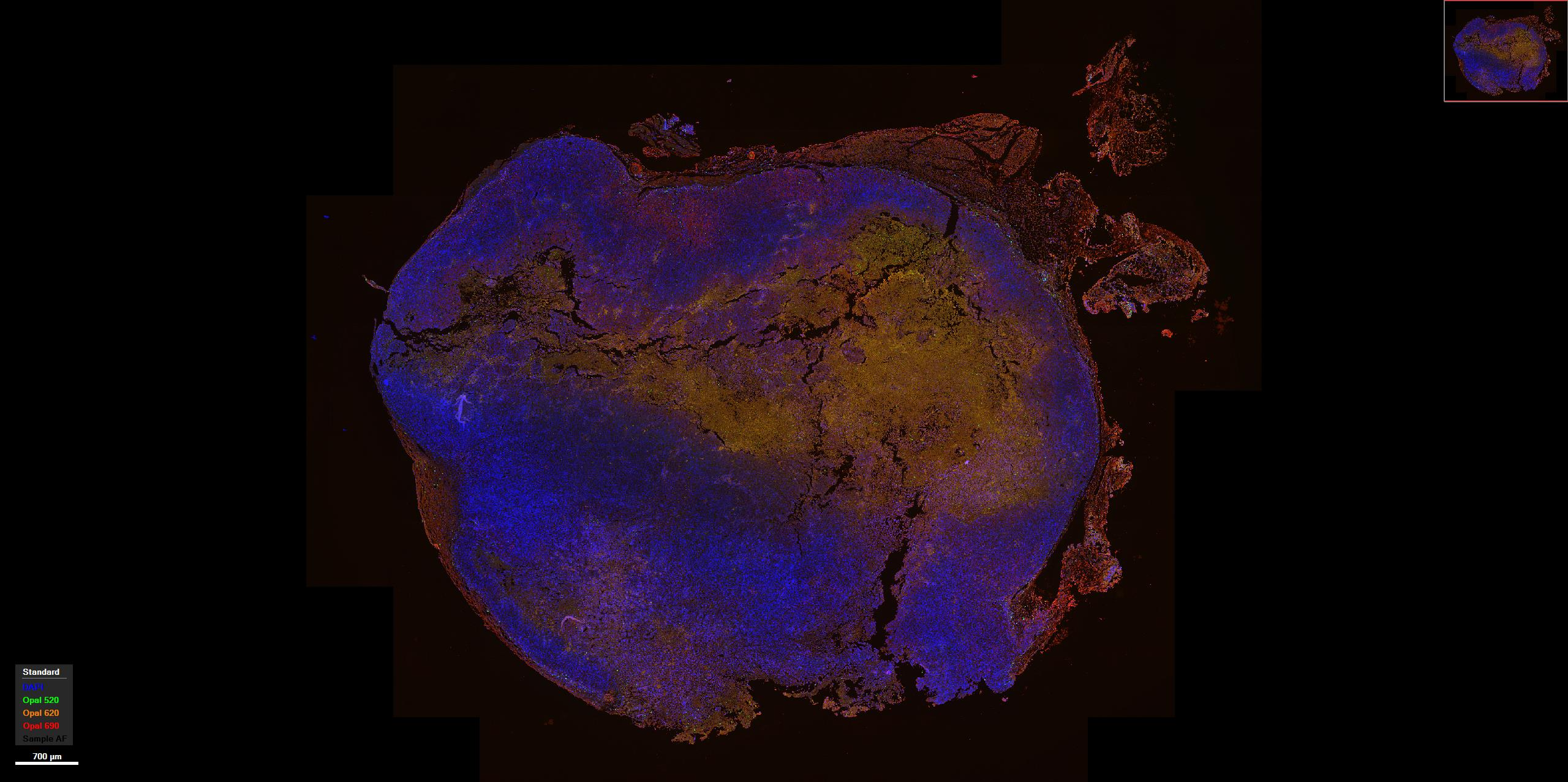

Supplement: Supplementary file 13 — Source data Fig. 5 [file 44321_2024_68_MOESM13_ESM.zip › Figure 5/Fig5B/mIHC_images_CD8:GzmB:TLR3/Tumor2_RT_overview.tiff]

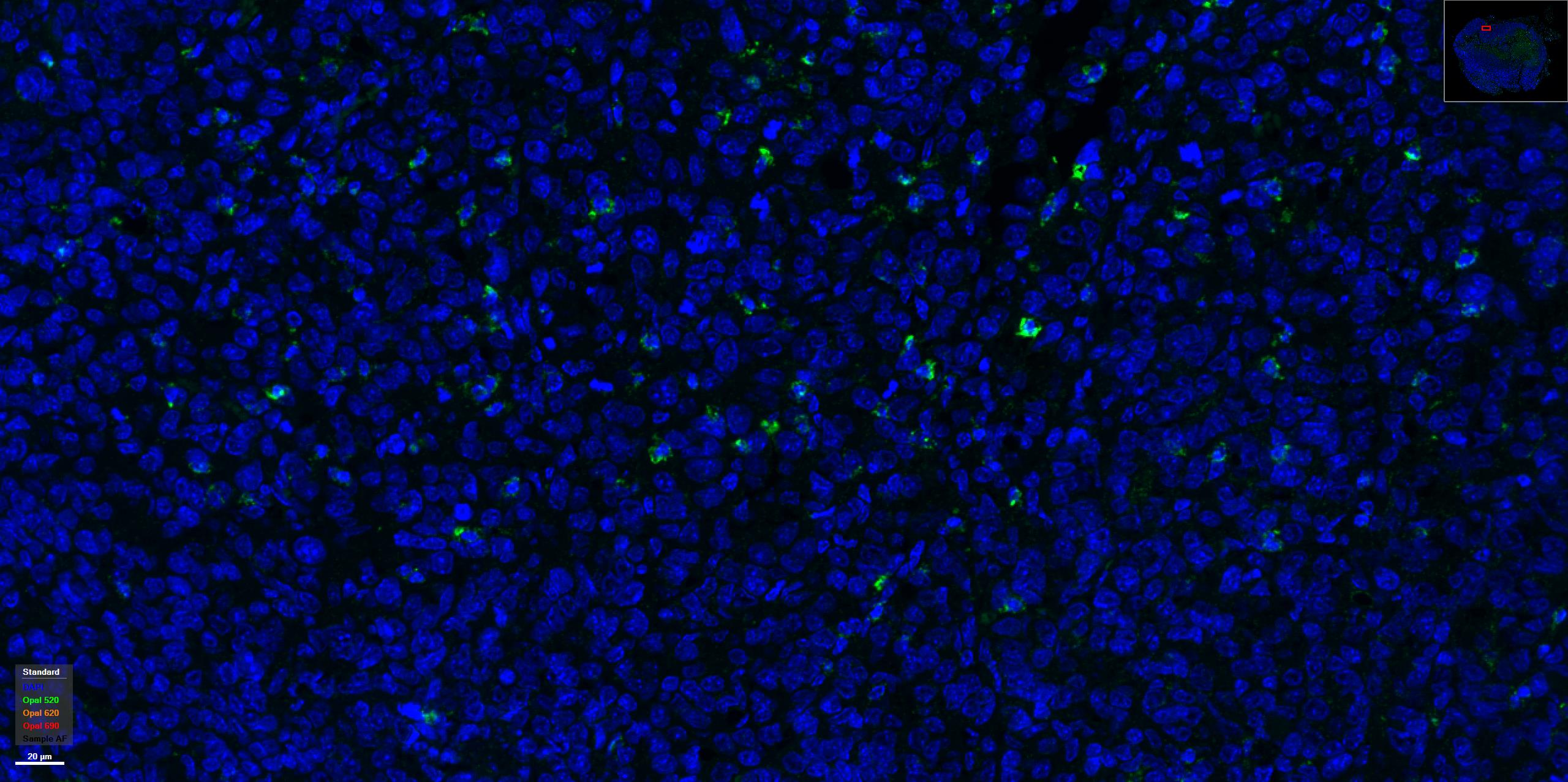

Supplement: Supplementary file 13 — Source data Fig. 5 [file 44321_2024_68_MOESM13_ESM.zip › Figure 5/Fig5B/mIHC_images_CD8:GzmB:TLR3/Tumor2_RT_CD8.tiff]

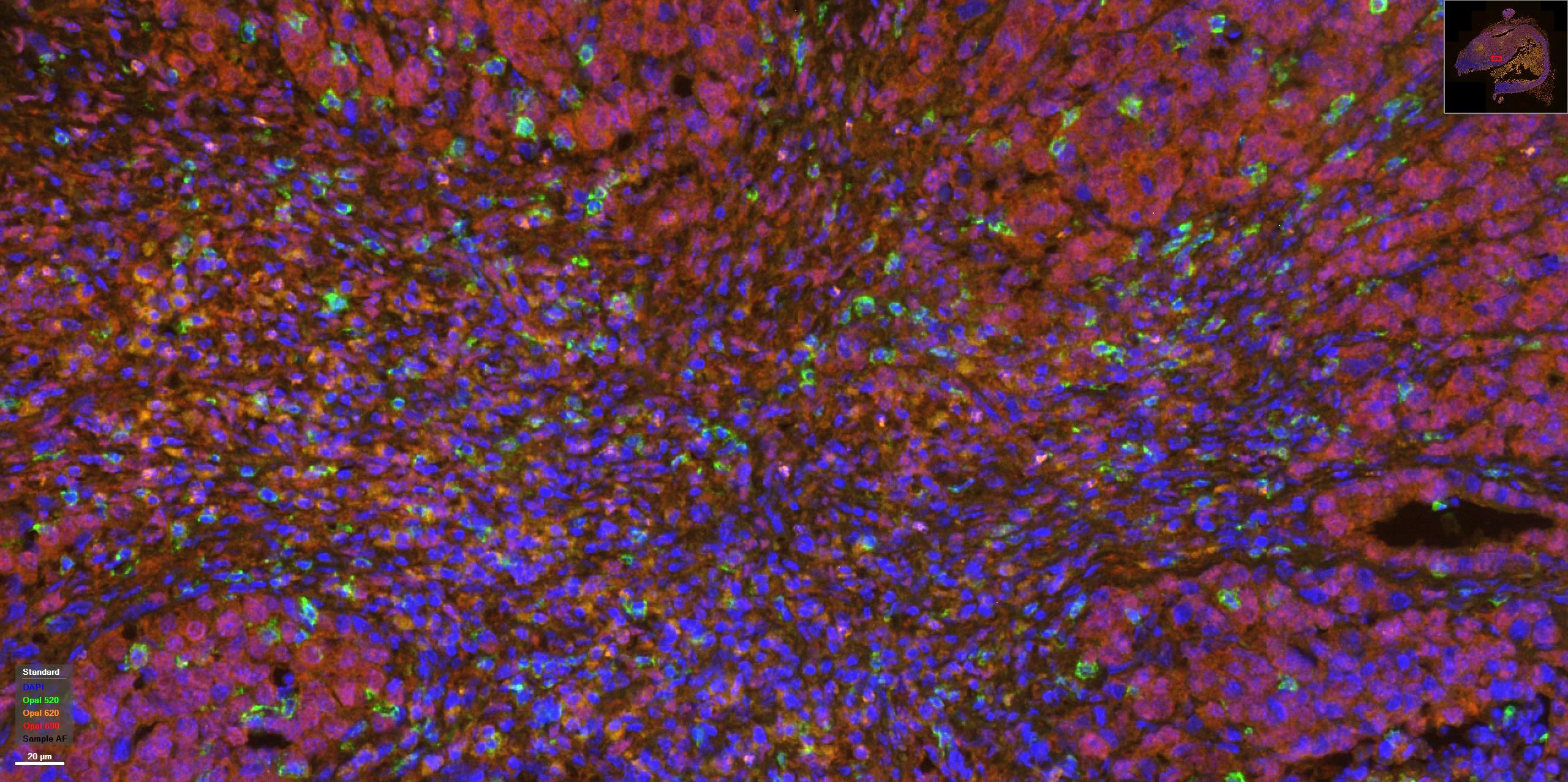

Supplement: Supplementary file 13 — Source data Fig. 5 [file 44321_2024_68_MOESM13_ESM.zip › Figure 5/Fig5B/mIHC_images_CD8:GzmB:TLR3/Tumor2_polyIC+RT_all.tiff]

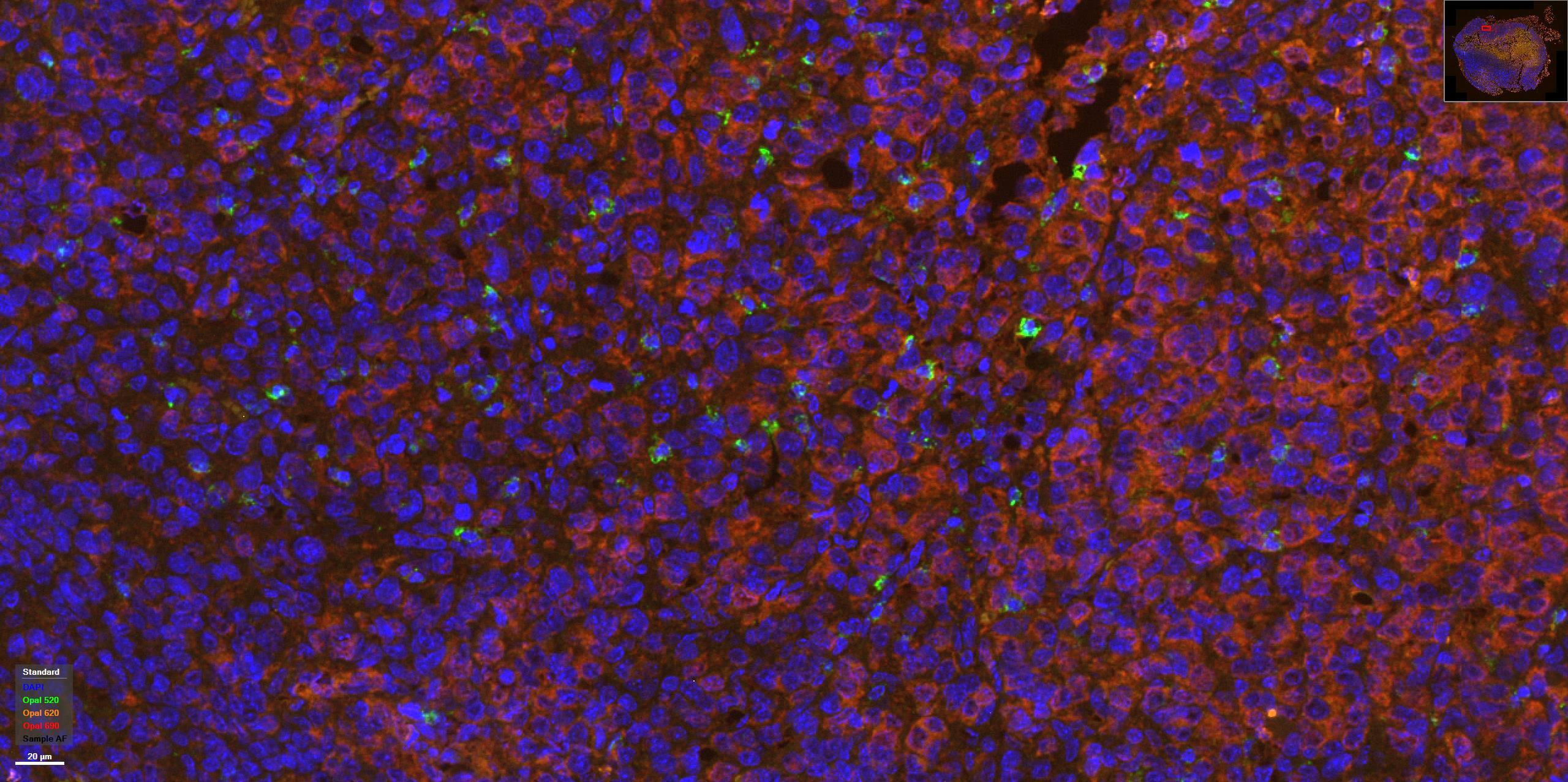

Supplement: Supplementary file 13 — Source data Fig. 5 [file 44321_2024_68_MOESM13_ESM.zip › Figure 5/Fig5B/mIHC_images_CD8:GzmB:TLR3/Tumor2_RT_all.tiff]

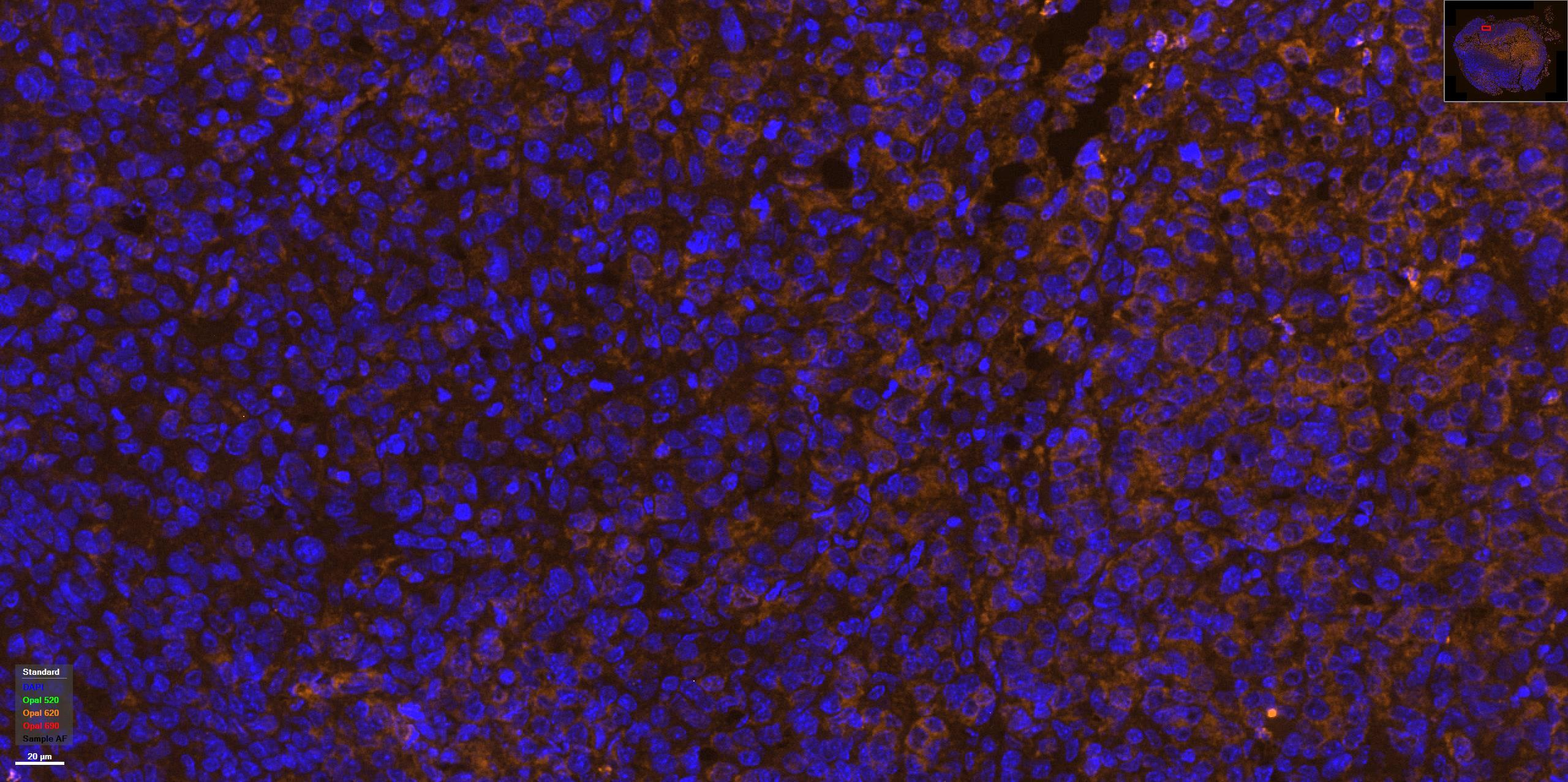

Supplement: Supplementary file 13 — Source data Fig. 5 [file 44321_2024_68_MOESM13_ESM.zip › Figure 5/Fig5B/mIHC_images_CD8:GzmB:TLR3/Tumor2_RT_GzmB.tiff]

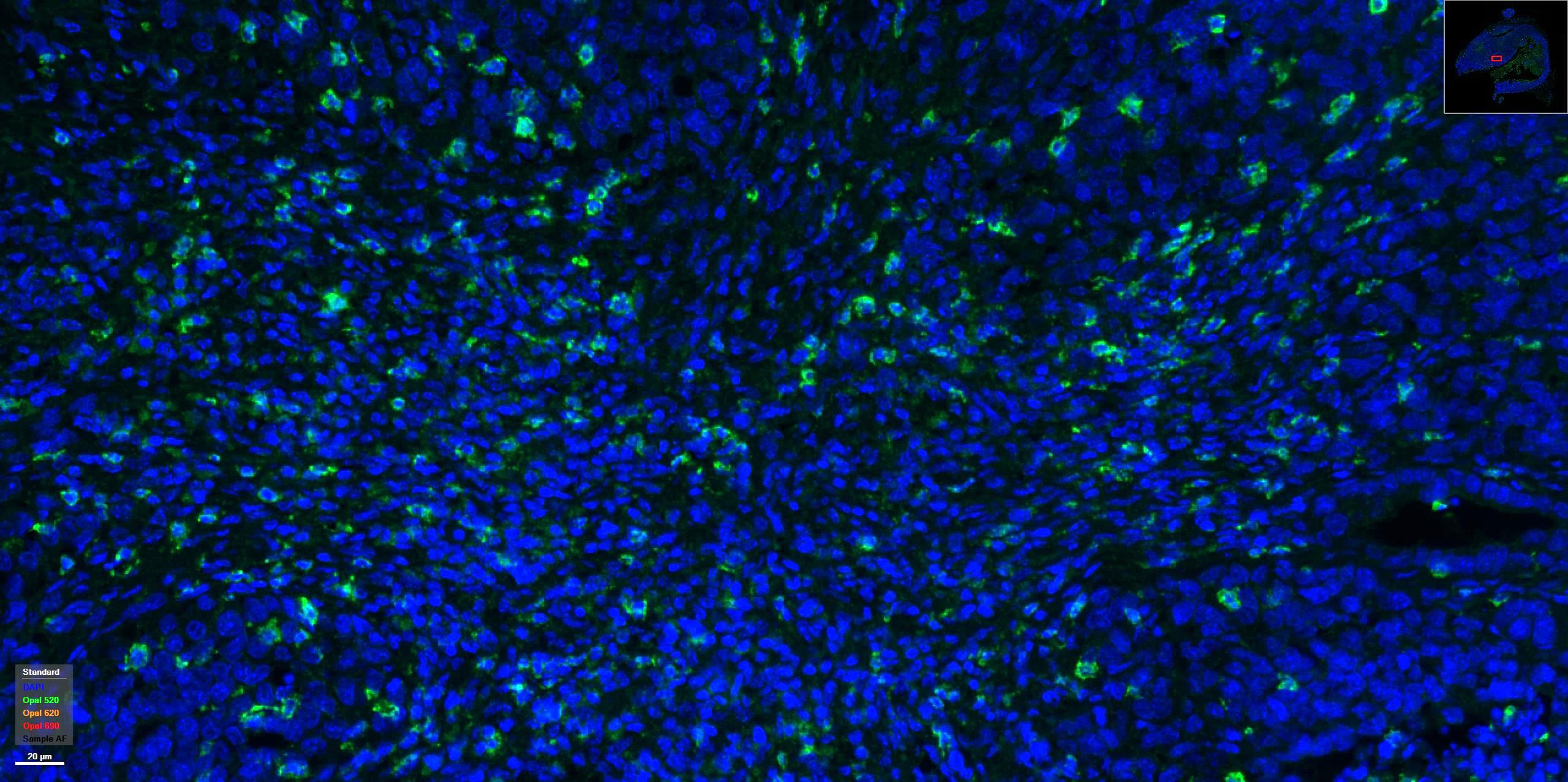

Supplement: Supplementary file 13 — Source data Fig. 5 [file 44321_2024_68_MOESM13_ESM.zip › Figure 5/Fig5B/mIHC_images_CD8:GzmB:TLR3/Tumor2_polyIC+RT_CD8.tiff]

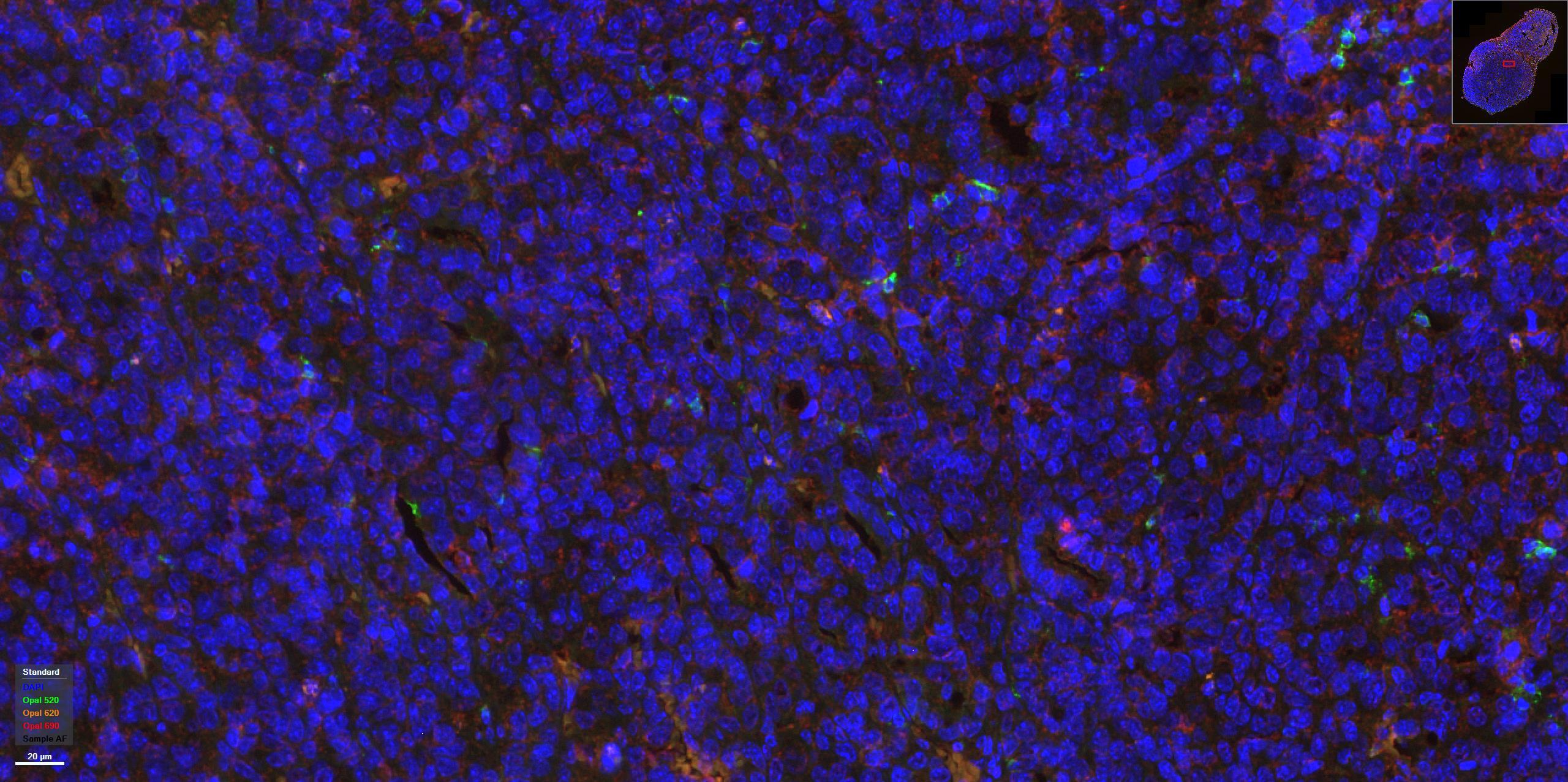

Supplement: Supplementary file 13 — Source data Fig. 5 [file 44321_2024_68_MOESM13_ESM.zip › Figure 5/Fig5B/mIHC_images_CD8:GzmB:TLR3/Tumor1_RT_all.tiff]

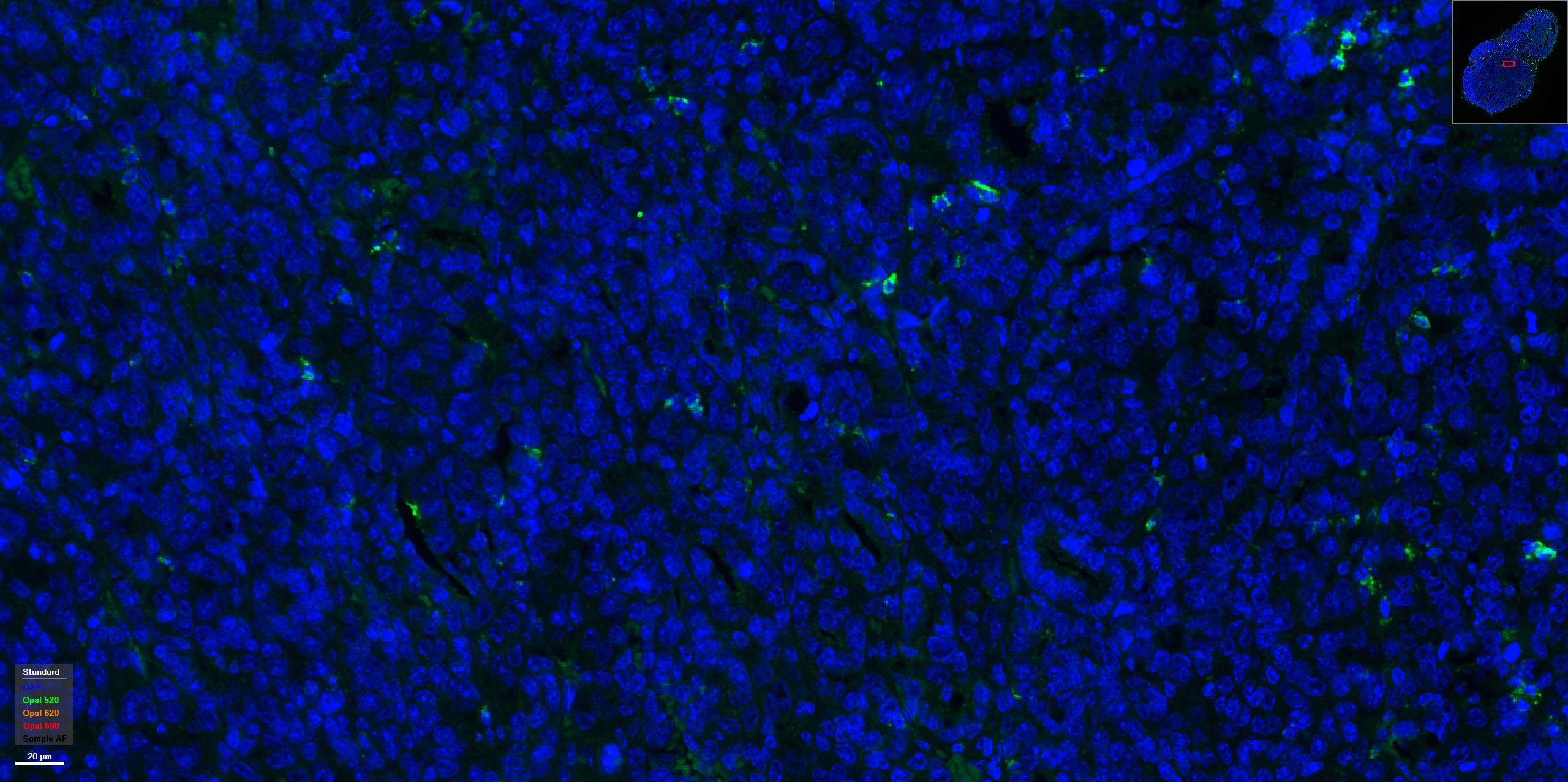

Supplement: Supplementary file 13 — Source data Fig. 5 [file 44321_2024_68_MOESM13_ESM.zip › Figure 5/Fig5B/mIHC_images_CD8:GzmB:TLR3/Tumor1_RT_CD8.tiff]

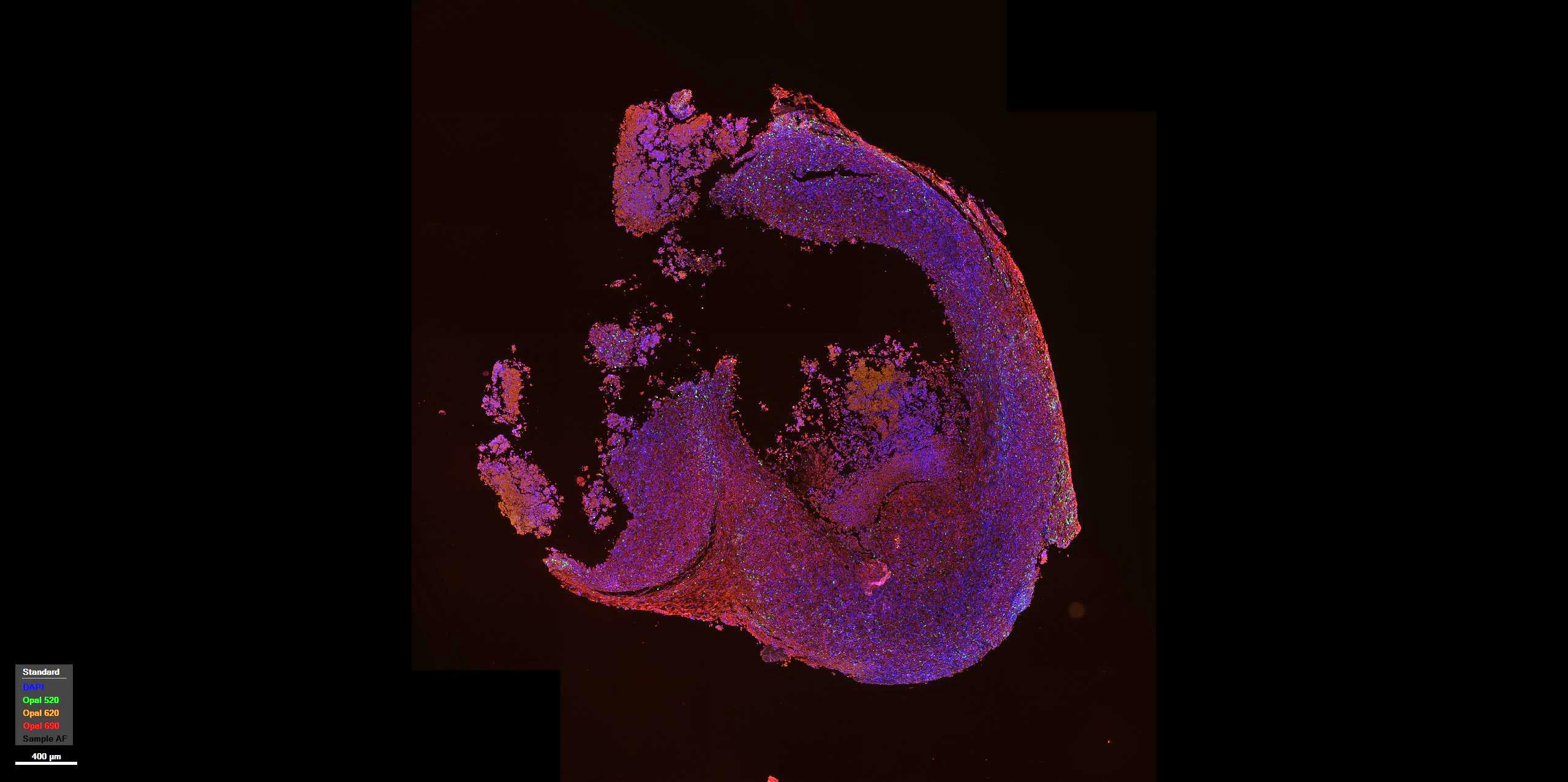

Supplement: Supplementary file 13 — Source data Fig. 5 [file 44321_2024_68_MOESM13_ESM.zip › Figure 5/Fig5B/mIHC_images_CD8:GzmB:TLR3/Tumor1_polyIC+RT_overview.tiff]

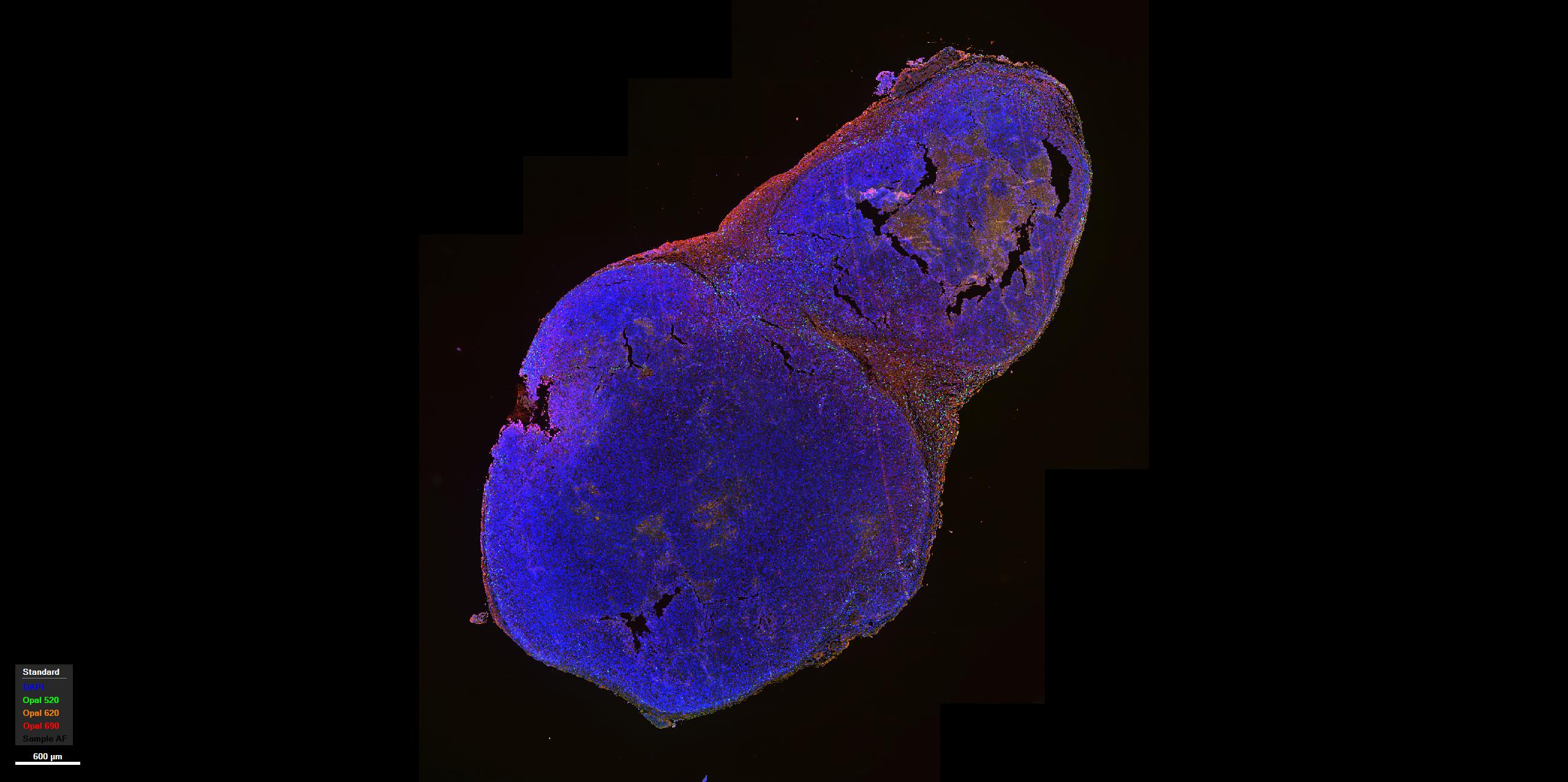

Supplement: Supplementary file 13 — Source data Fig. 5 [file 44321_2024_68_MOESM13_ESM.zip › Figure 5/Fig5B/mIHC_images_CD8:GzmB:TLR3/Tumor1_RT_overview.tiff]

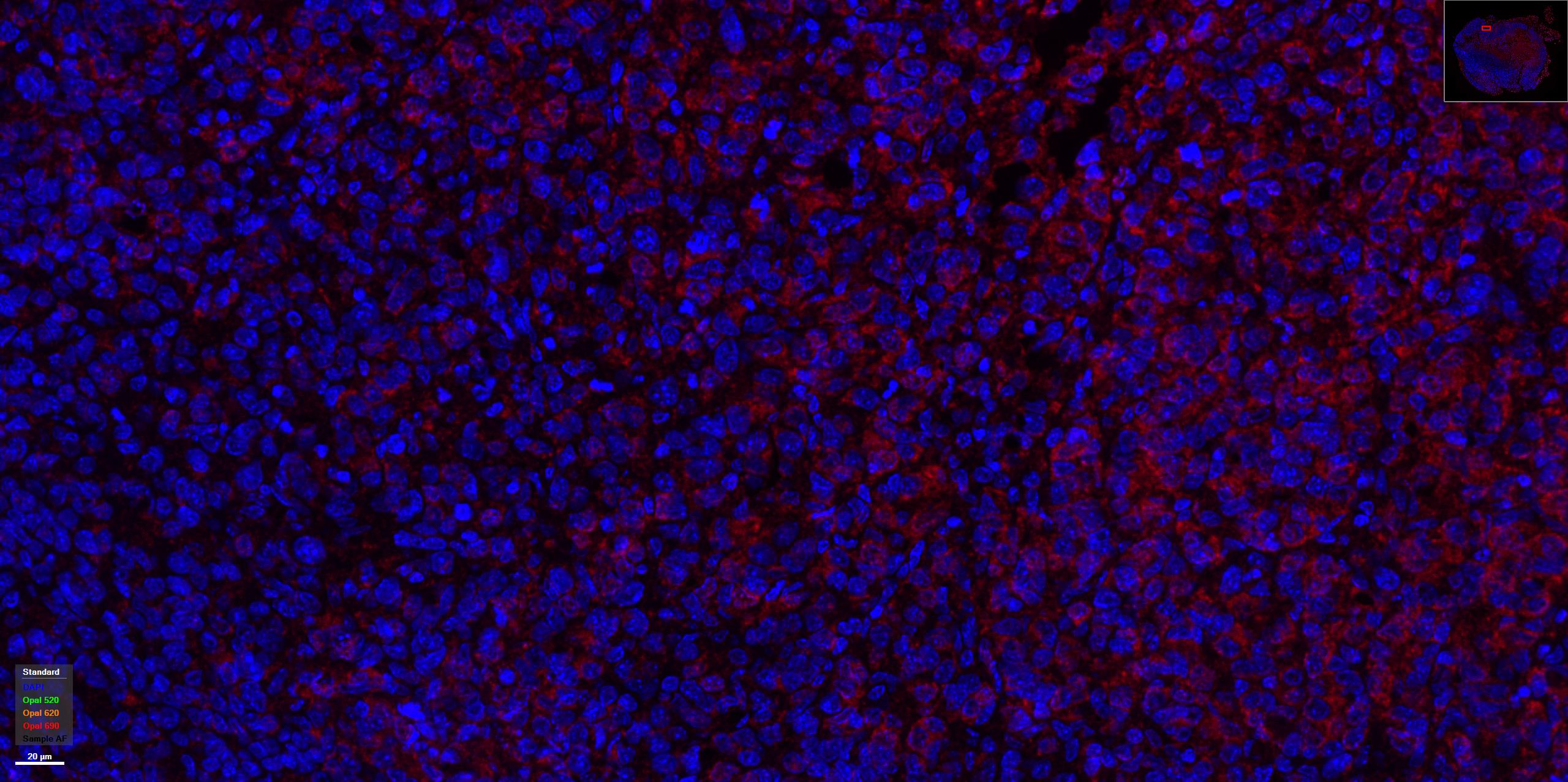

Supplement: Supplementary file 13 — Source data Fig. 5 [file 44321_2024_68_MOESM13_ESM.zip › Figure 5/Fig5B/mIHC_images_CD8:GzmB:TLR3/Tumor2_RT_TLR3.tiff]

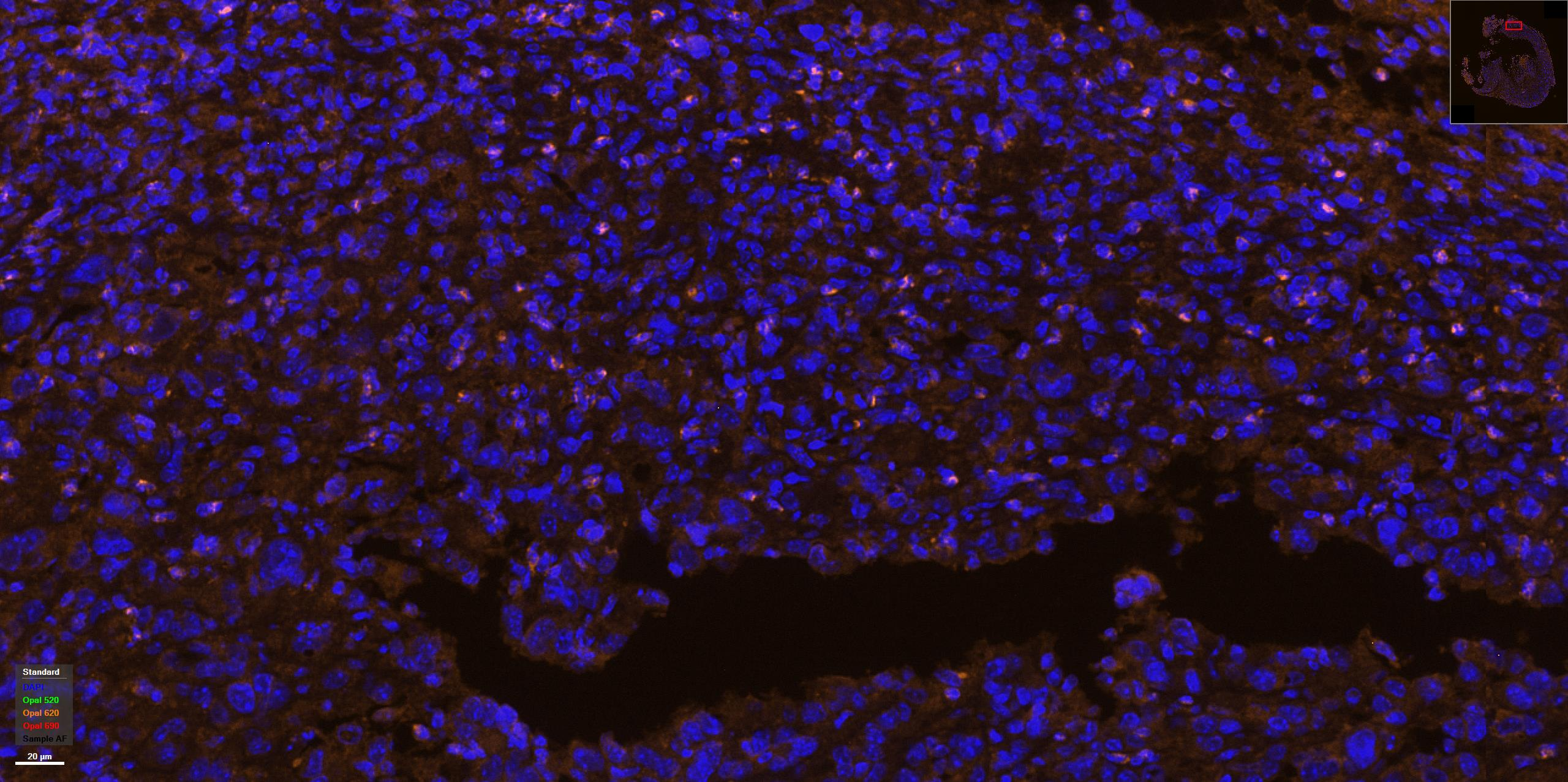

Supplement: Supplementary file 13 — Source data Fig. 5 [file 44321_2024_68_MOESM13_ESM.zip › Figure 5/Fig5B/mIHC_images_CD8:GzmB:TLR3/Tumor1_polyIC+RT_GzmB.tiff]

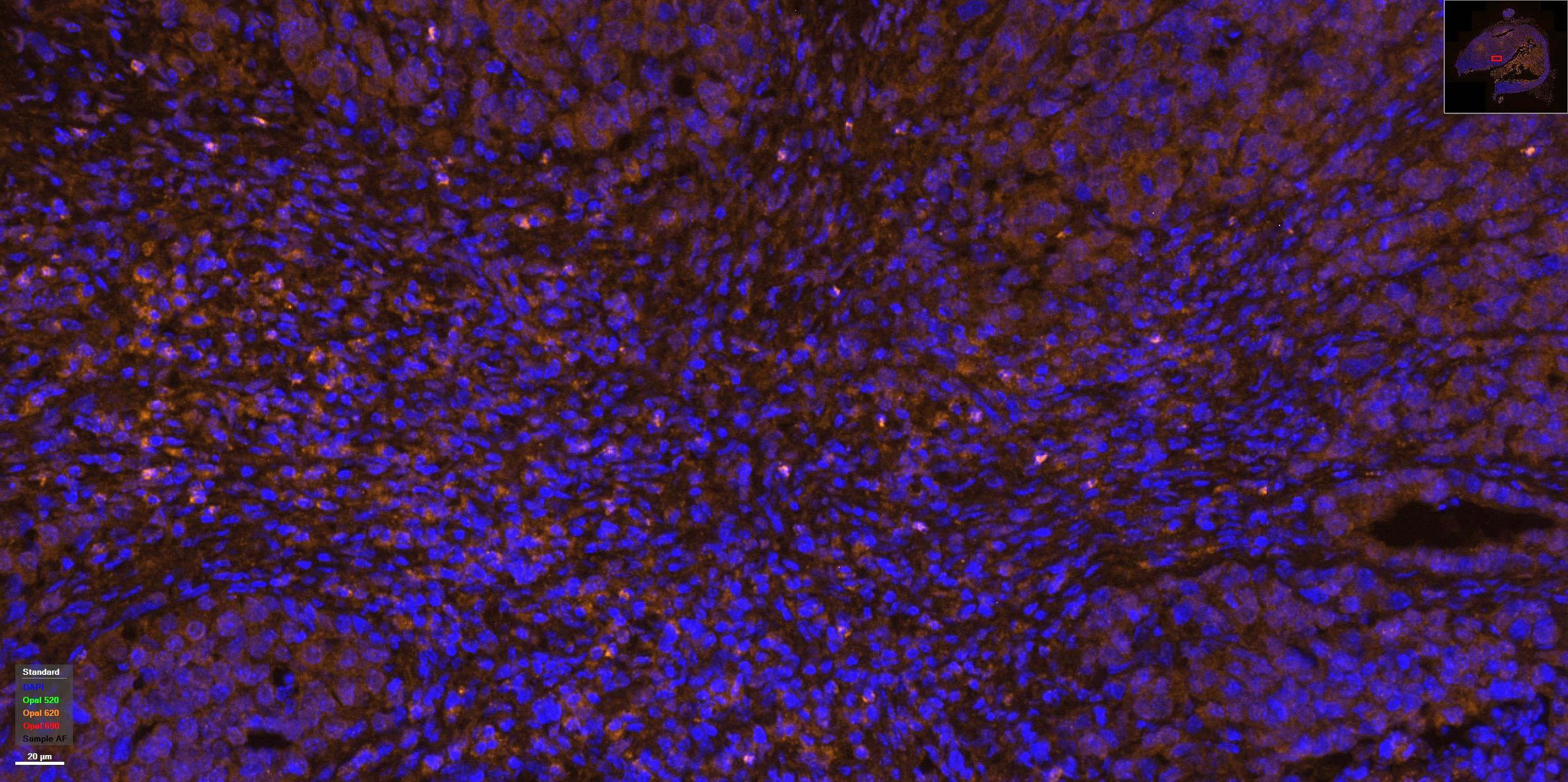

Supplement: Supplementary file 13 — Source data Fig. 5 [file 44321_2024_68_MOESM13_ESM.zip › Figure 5/Fig5B/mIHC_images_CD8:GzmB:TLR3/Tumor2_polyIC+RT_GzmB.tiff]

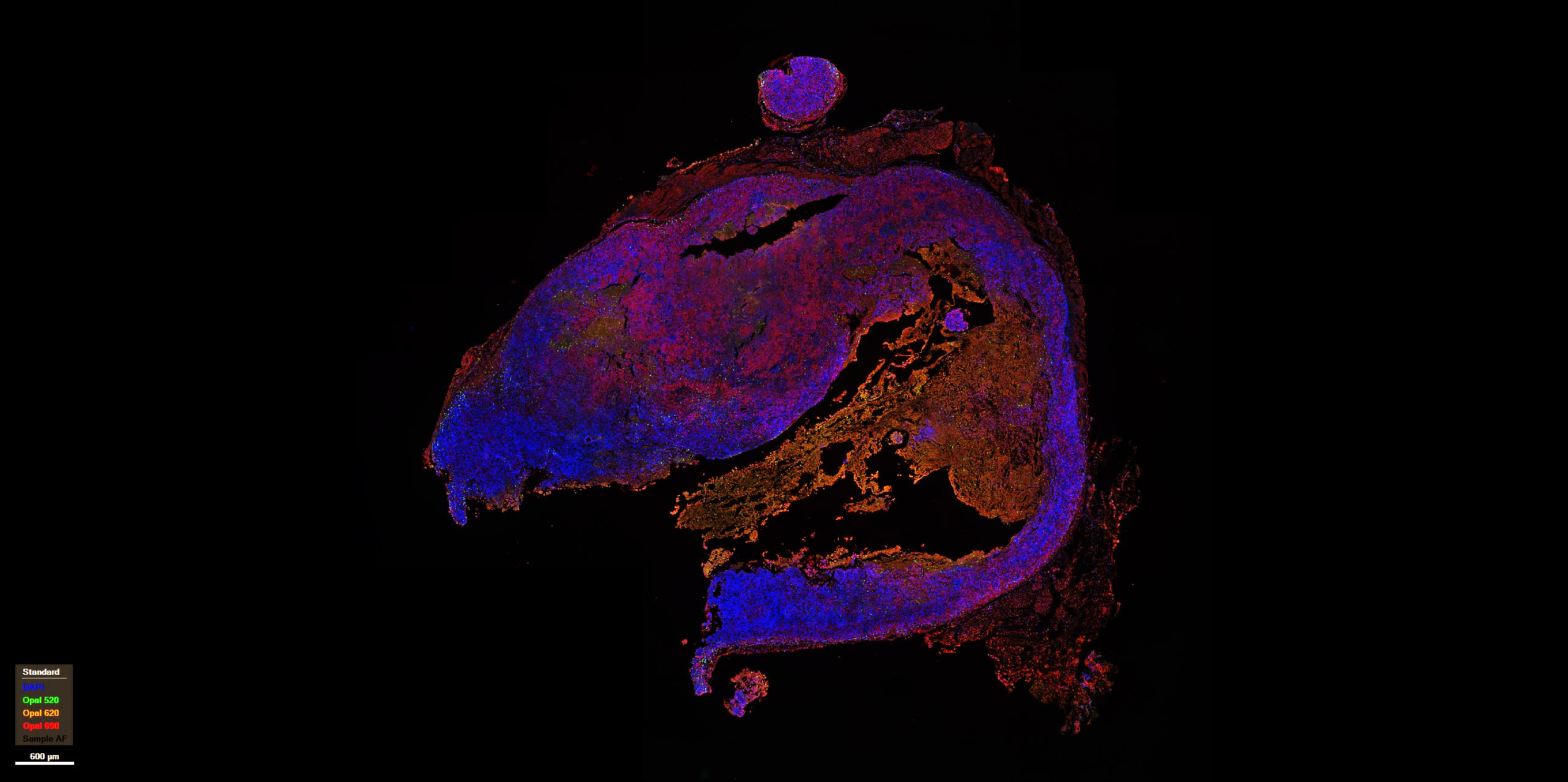

Supplement: Supplementary file 13 — Source data Fig. 5 [file 44321_2024_68_MOESM13_ESM.zip › Figure 5/Fig5B/mIHC_images_CD8:GzmB:TLR3/Tumor2_polyIC+RT_overview.tiff]

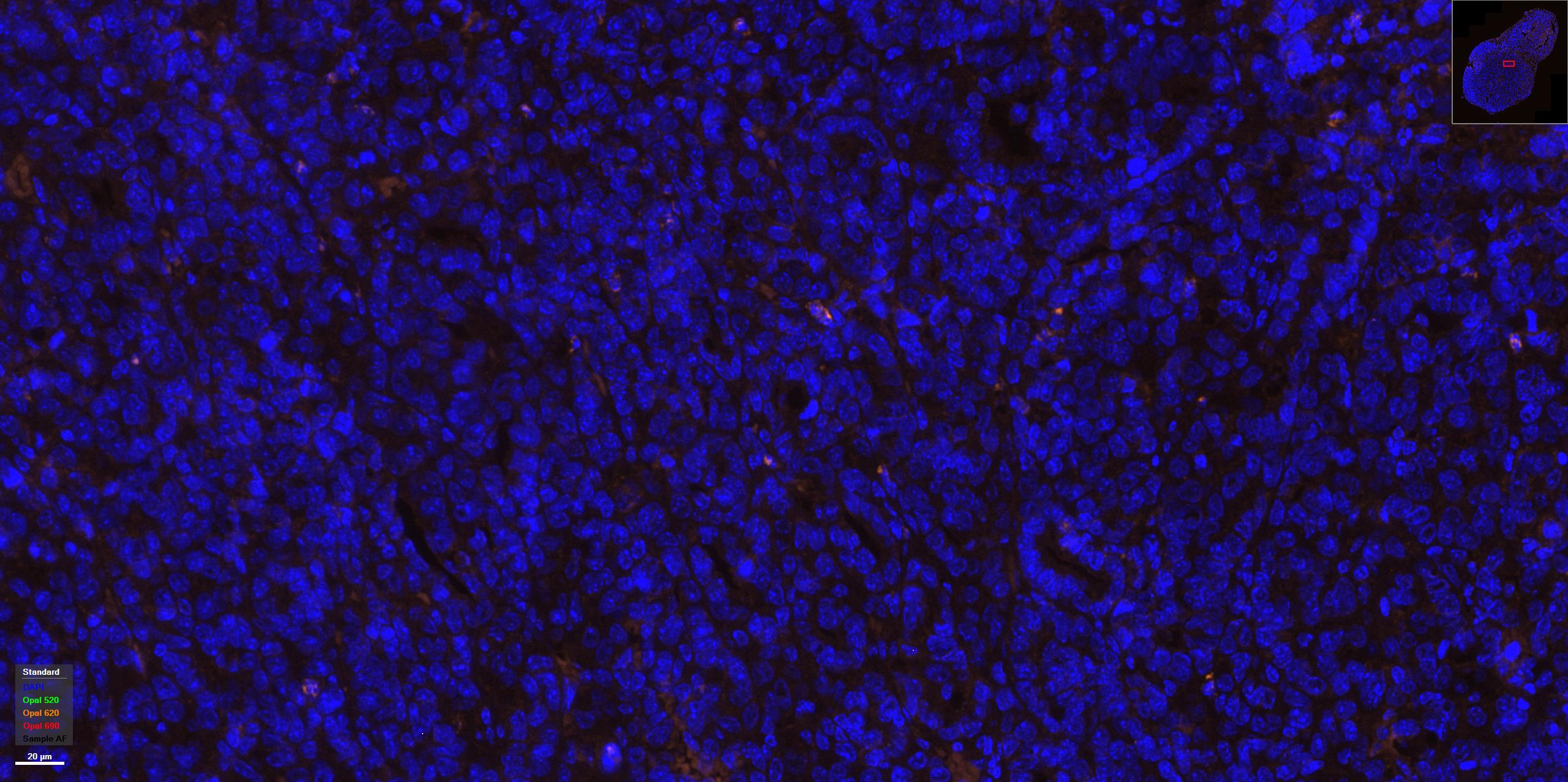

Supplement: Supplementary file 13 — Source data Fig. 5 [file 44321_2024_68_MOESM13_ESM.zip › Figure 5/Fig5B/mIHC_images_CD8:GzmB:TLR3/Tumor1_RT_GzmB.tiff]

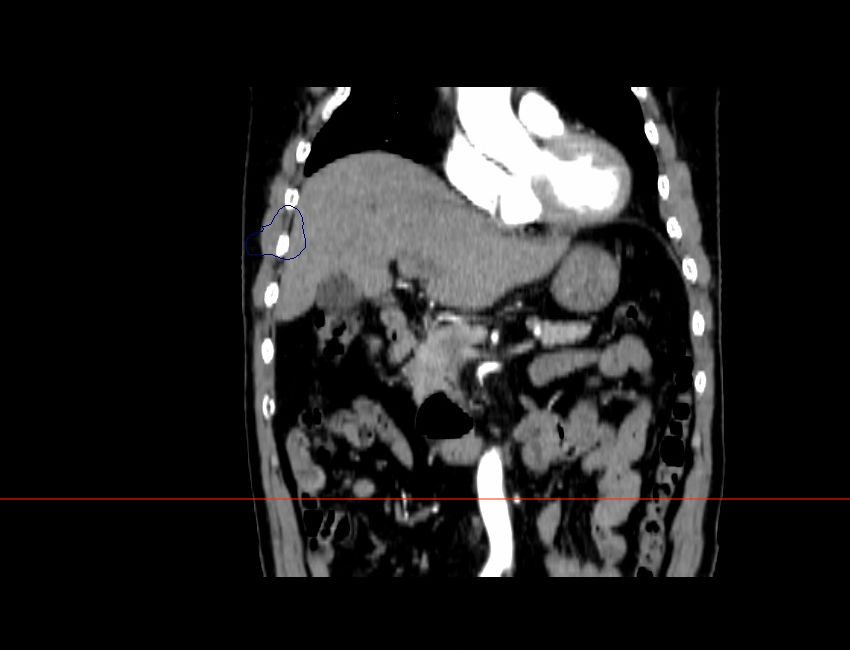

Supplement: Supplementary file 15 — Source data Fig. 7 [file 44321_2024_68_MOESM15_ESM.zip › Figure 7/Fig7B/Radiotherapy schedule for P04&P05_images/P05_Distant lesion_Coronal.tiff]

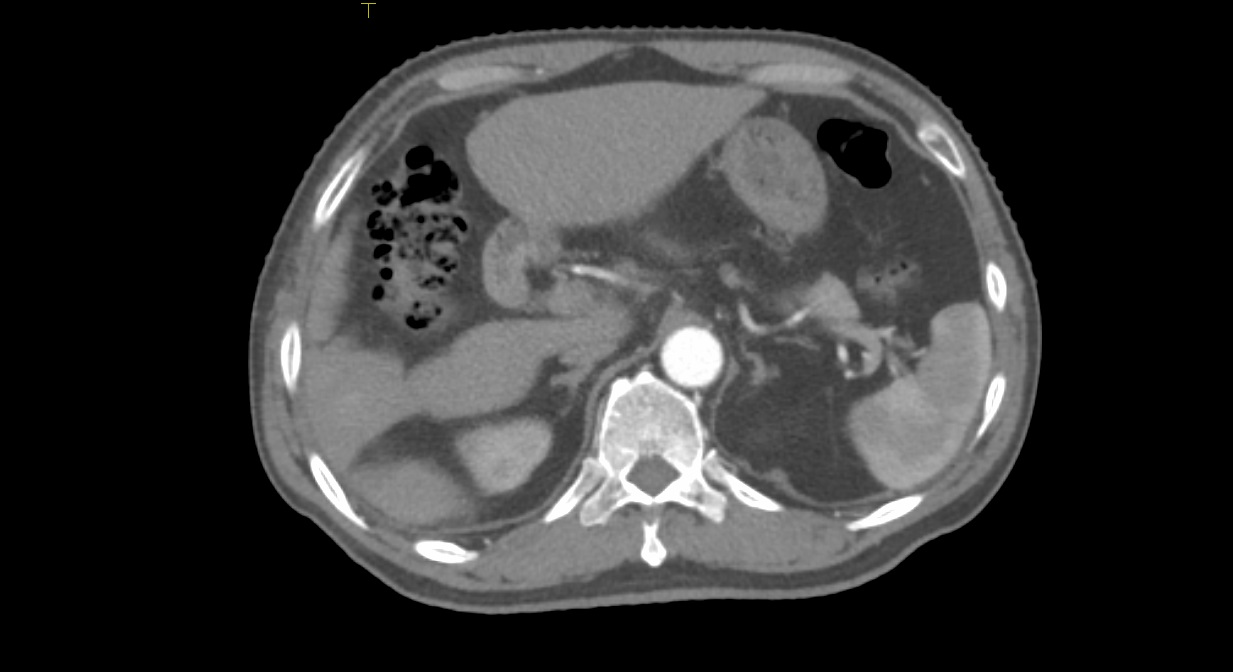

Supplement: Supplementary file 15 — Source data Fig. 7 [file 44321_2024_68_MOESM15_ESM.zip › Figure 7/Fig7B/Radiotherapy schedule for P04&P05_images/P05_Distant lesion_transverse.tiff]

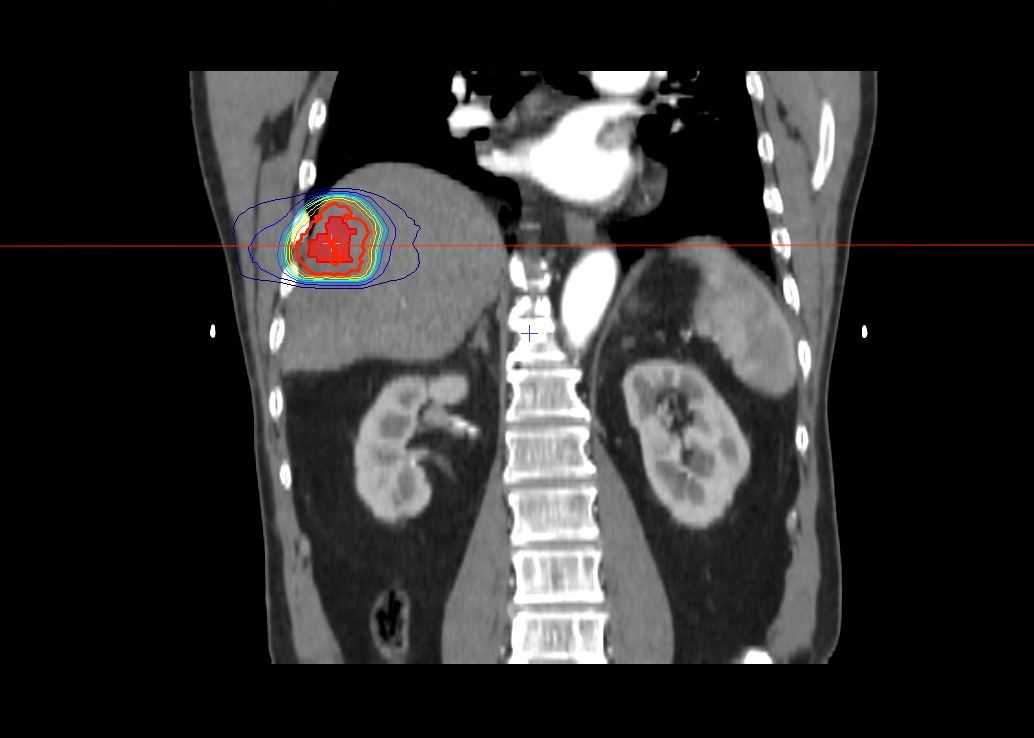

Supplement: Supplementary file 15 — Source data Fig. 7 [file 44321_2024_68_MOESM15_ESM.zip › Figure 7/Fig7B/Radiotherapy schedule for P04&P05_images/P05_Irradiated lesion_Coronal.tiff]

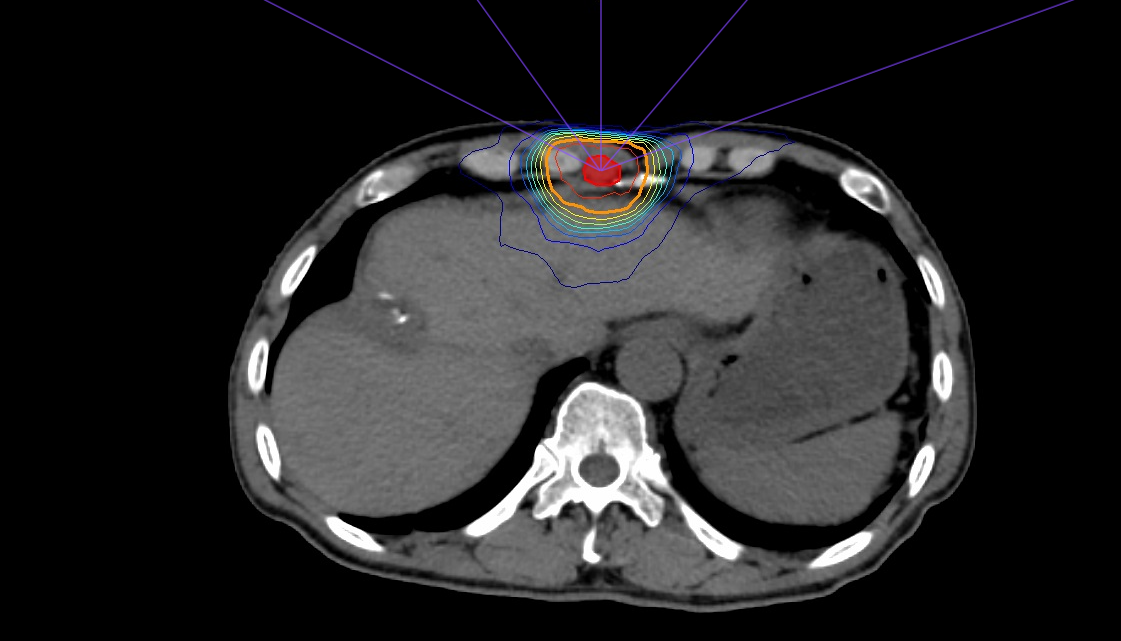

Supplement: Supplementary file 15 — Source data Fig. 7 [file 44321_2024_68_MOESM15_ESM.zip › Figure 7/Fig7B/Radiotherapy schedule for P04&P05_images/P04_Irradiated lesion_transverse.tiff]

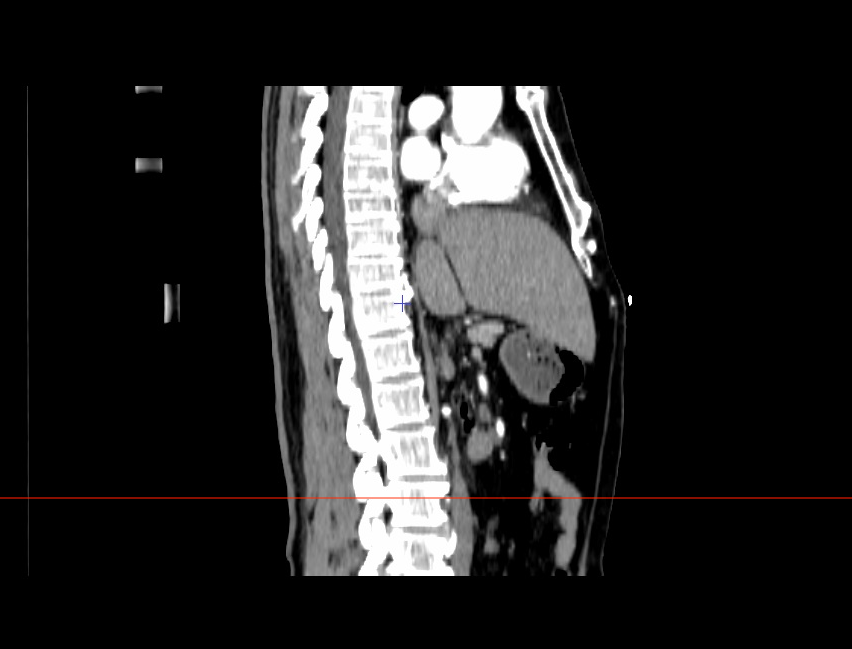

Supplement: Supplementary file 15 — Source data Fig. 7 [file 44321_2024_68_MOESM15_ESM.zip › Figure 7/Fig7B/Radiotherapy schedule for P04&P05_images/P05_Distant lesion_sagittal.tiff]

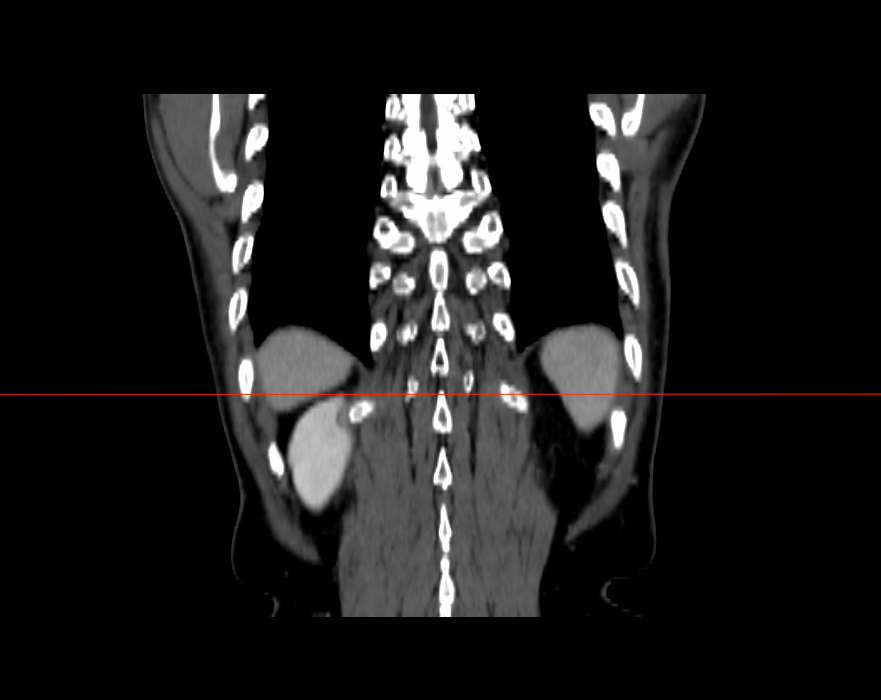

Supplement: Supplementary file 15 — Source data Fig. 7 [file 44321_2024_68_MOESM15_ESM.zip › Figure 7/Fig7B/Radiotherapy schedule for P04&P05_images/P04_Distant lesion_Coronal.tiff]

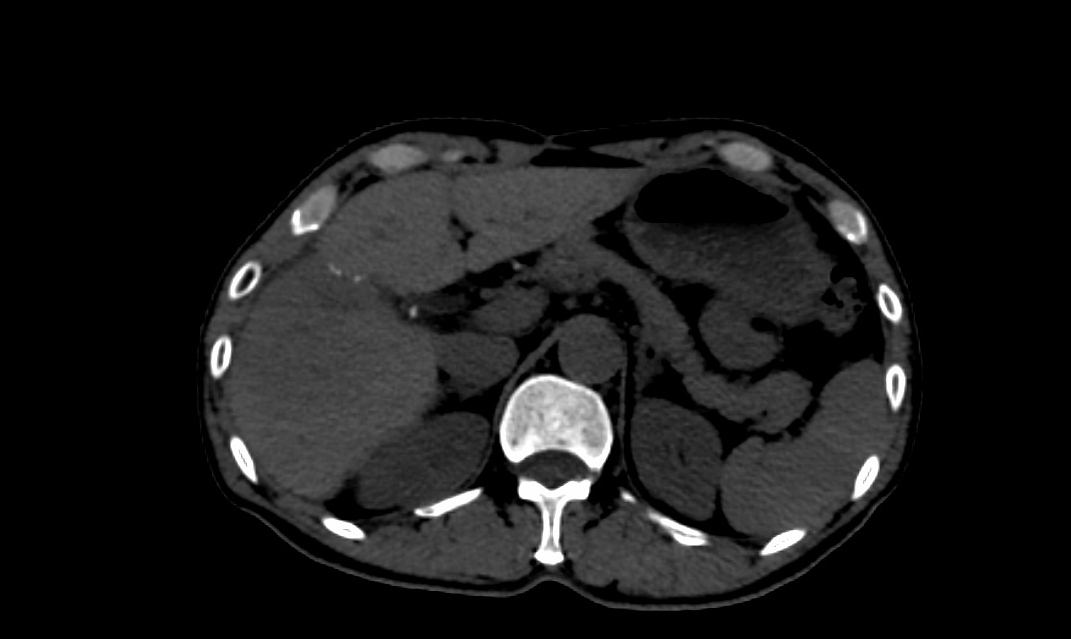

Supplement: Supplementary file 15 — Source data Fig. 7 [file 44321_2024_68_MOESM15_ESM.zip › Figure 7/Fig7B/Radiotherapy schedule for P04&P05_images/P04_Distant lesion_transverse.tiff]

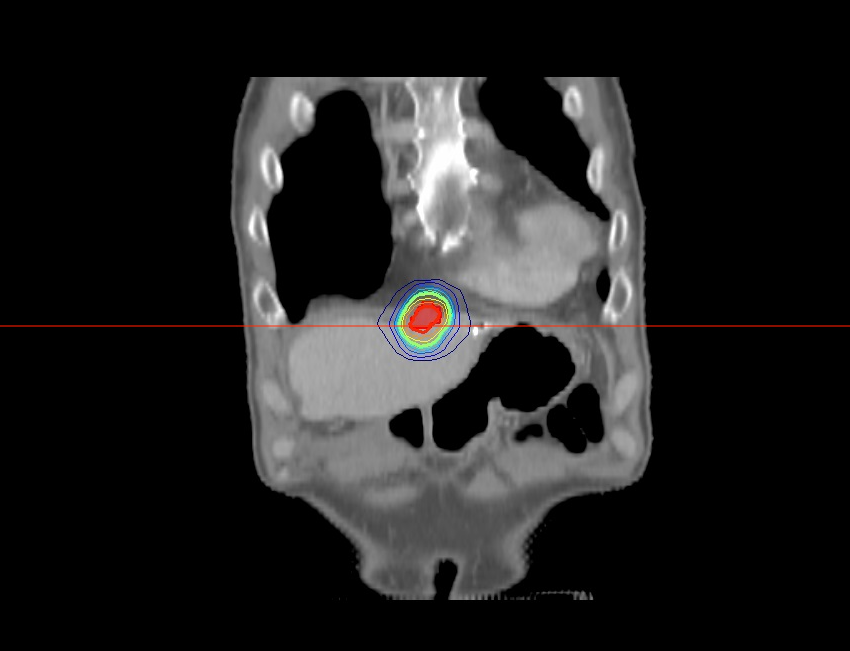

Supplement: Supplementary file 15 — Source data Fig. 7 [file 44321_2024_68_MOESM15_ESM.zip › Figure 7/Fig7B/Radiotherapy schedule for P04&P05_images/P04_Irradiated lesion_Coronal.tiff]

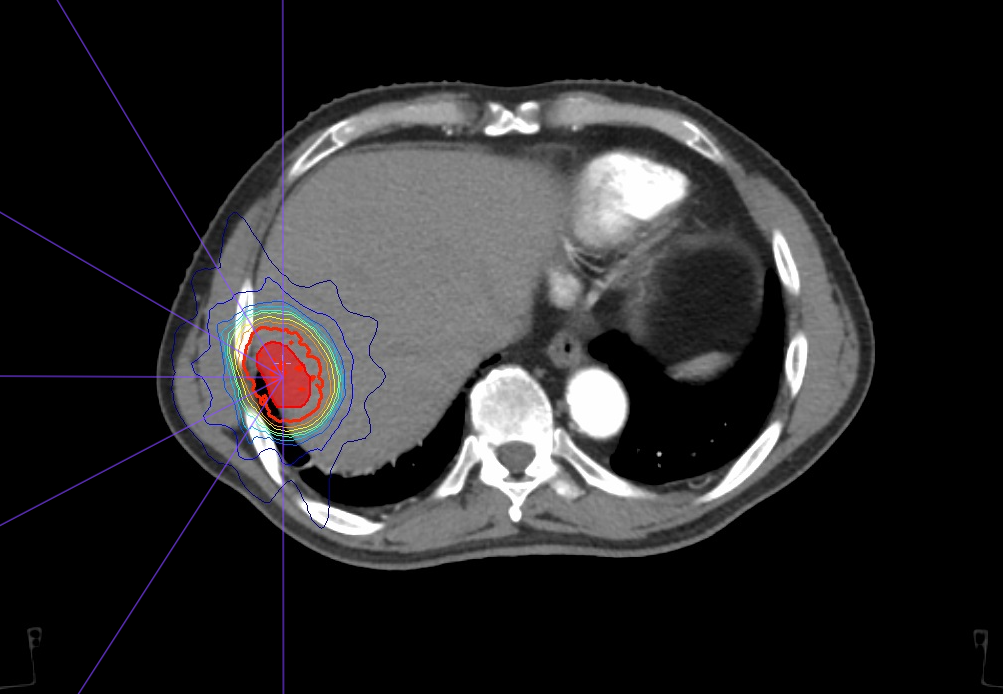

Supplement: Supplementary file 15 — Source data Fig. 7 [file 44321_2024_68_MOESM15_ESM.zip › Figure 7/Fig7B/Radiotherapy schedule for P04&P05_images/P05_Irradiated lesion_transverse.tiff]

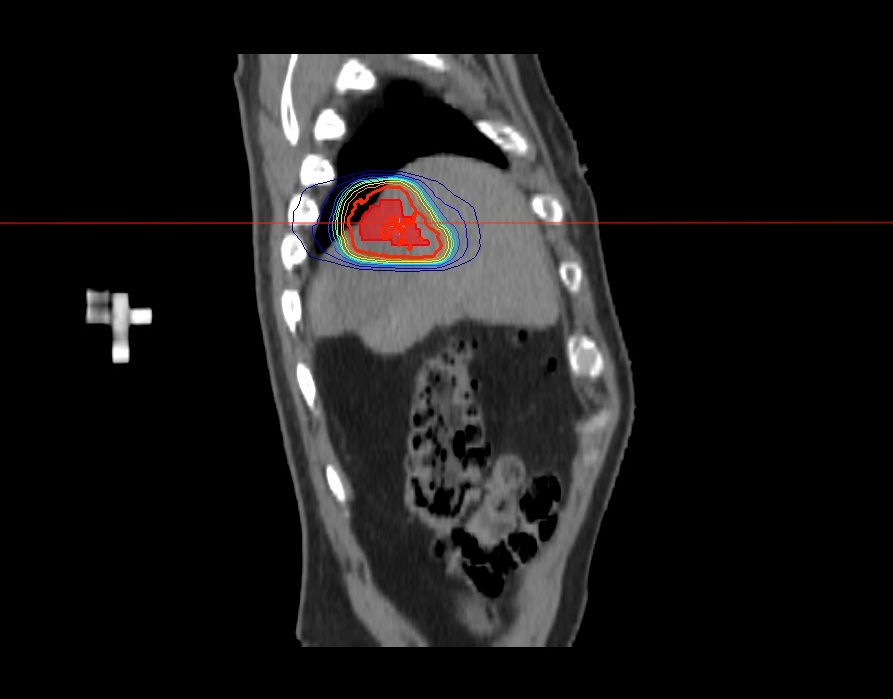

Supplement: Supplementary file 15 — Source data Fig. 7 [file 44321_2024_68_MOESM15_ESM.zip › Figure 7/Fig7B/Radiotherapy schedule for P04&P05_images/P05_Irradiated lesion_sagittal.tiff]

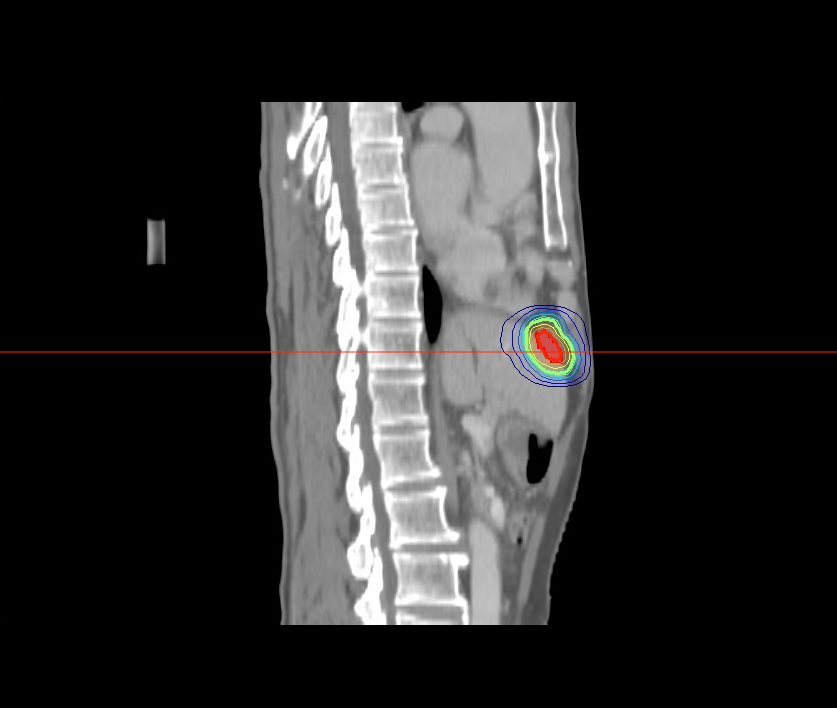

Supplement: Supplementary file 15 — Source data Fig. 7 [file 44321_2024_68_MOESM15_ESM.zip › Figure 7/Fig7B/Radiotherapy schedule for P04&P05_images/P04_Irradiated lesion_sagittal.tiff]

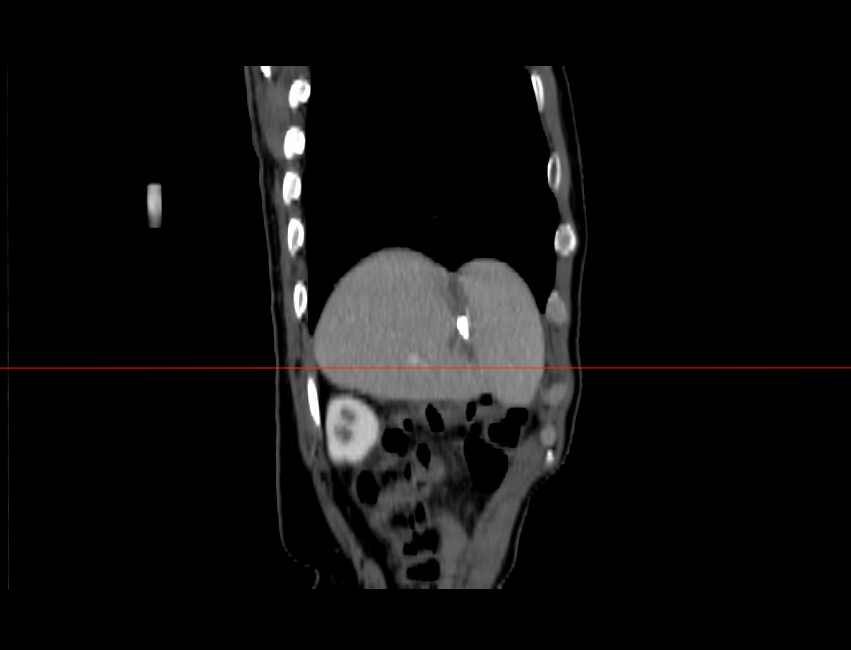

Supplement: Supplementary file 15 — Source data Fig. 7 [file 44321_2024_68_MOESM15_ESM.zip › Figure 7/Fig7B/Radiotherapy schedule for P04&P05_images/P04_Distant lesion_sagittal.tiff]

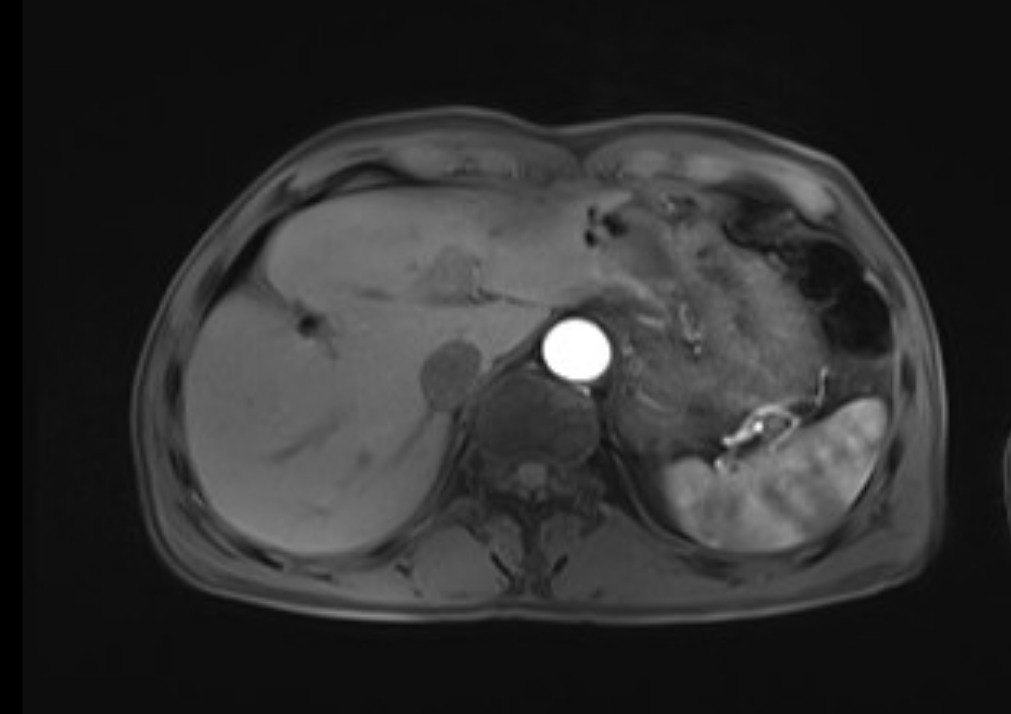

Supplement: Supplementary file 15 — Source data Fig. 7 [file 44321_2024_68_MOESM15_ESM.zip › Figure 7/Fig7C/P04_images/P04_Irradiated lesion_post.tiff]

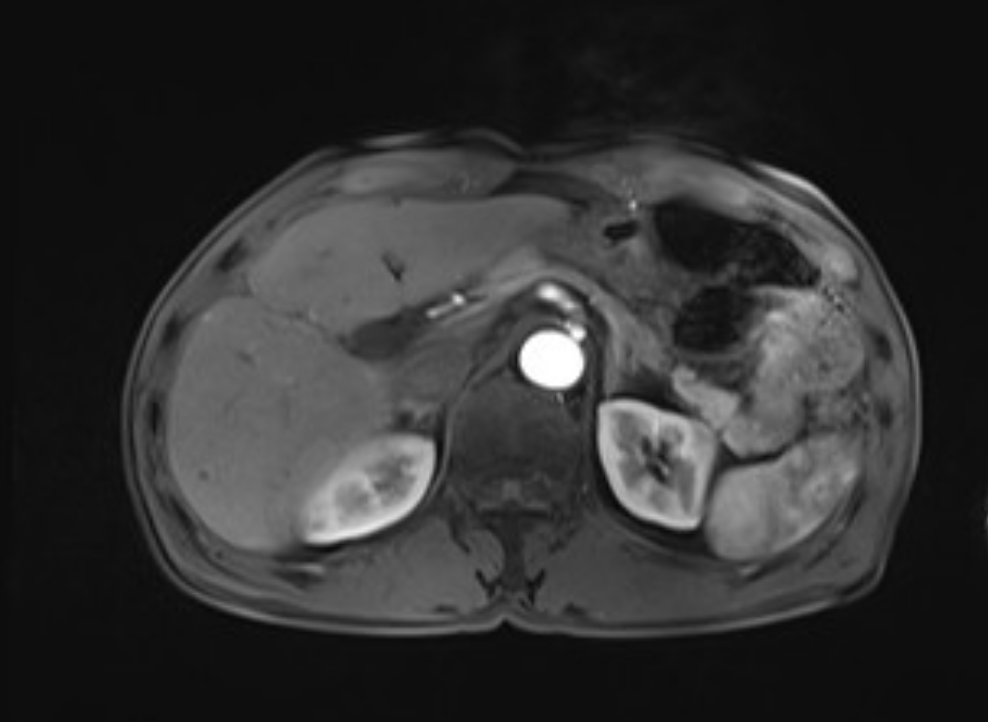

Supplement: Supplementary file 15 — Source data Fig. 7 [file 44321_2024_68_MOESM15_ESM.zip › Figure 7/Fig7C/P04_images/P04_Distant lesion_post.tiff]

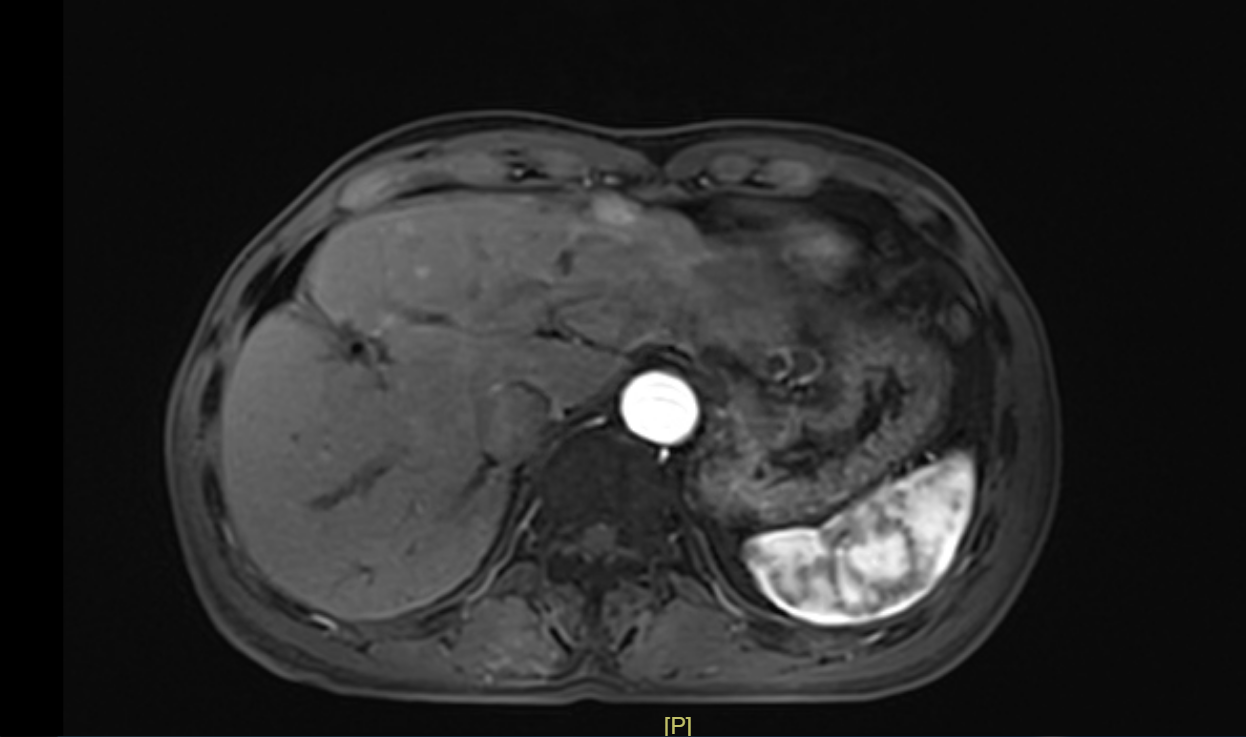

Supplement: Supplementary file 15 — Source data Fig. 7 [file 44321_2024_68_MOESM15_ESM.zip › Figure 7/Fig7C/P04_images/P04_Irradiated lesion_pre.tiff]

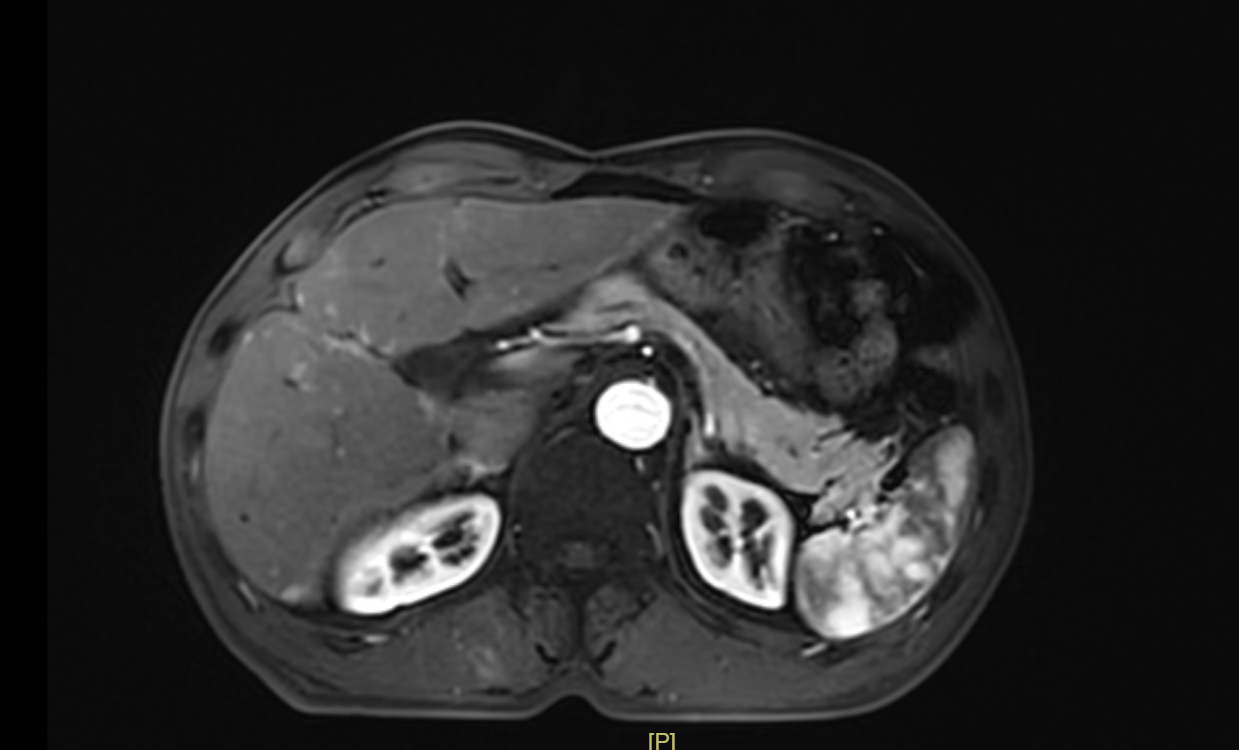

Supplement: Supplementary file 15 — Source data Fig. 7 [file 44321_2024_68_MOESM15_ESM.zip › Figure 7/Fig7C/P04_images/P04_Distant lesion_pre.tiff]

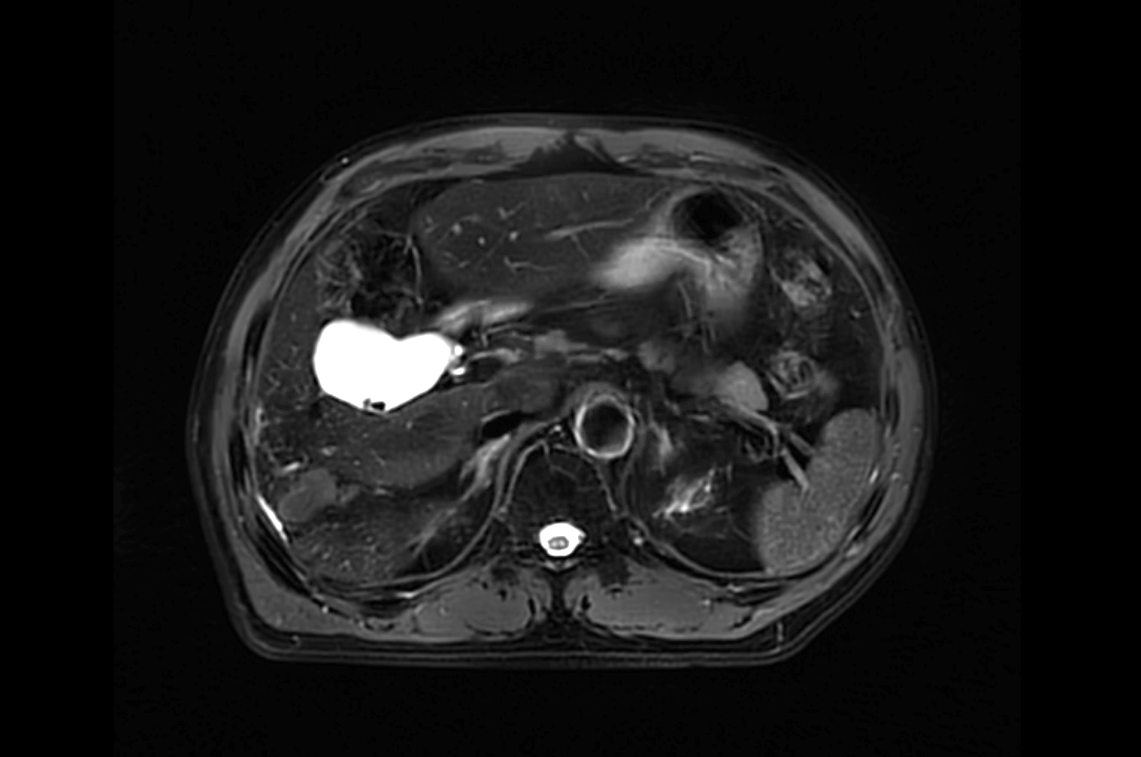

Supplement: Supplementary file 15 — Source data Fig. 7 [file 44321_2024_68_MOESM15_ESM.zip › Figure 7/Fig7D/P05_images/P05_Distant lesion_Pre.tiff]

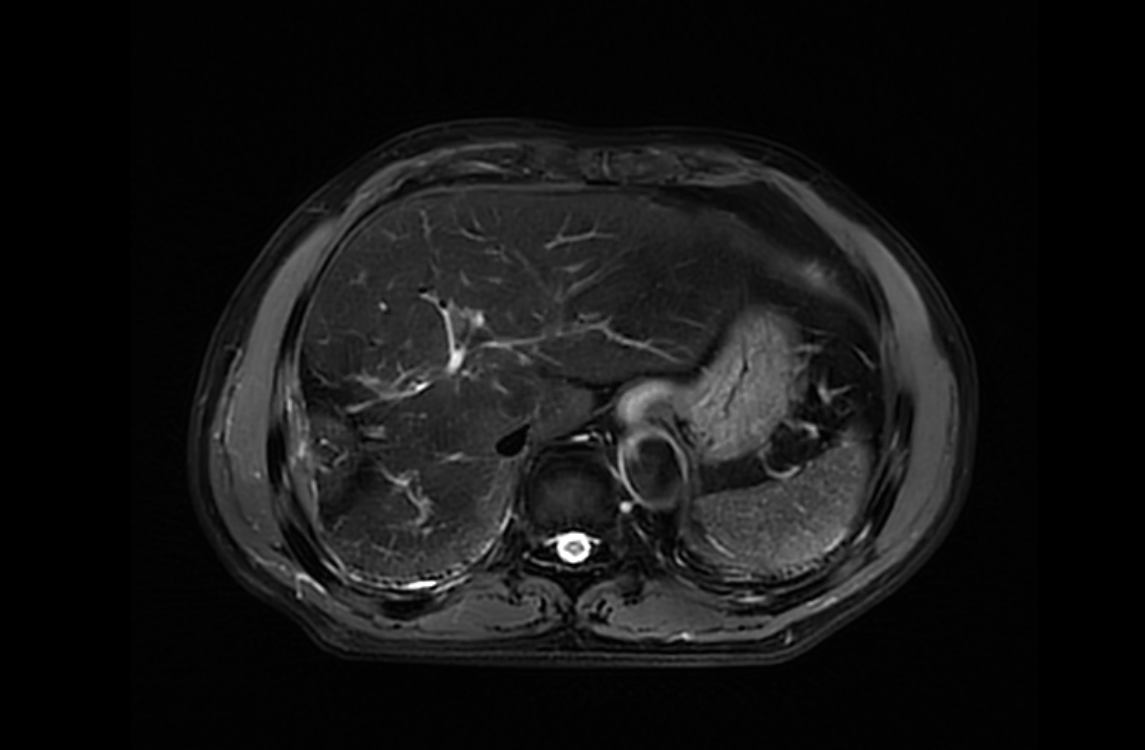

Supplement: Supplementary file 15 — Source data Fig. 7 [file 44321_2024_68_MOESM15_ESM.zip › Figure 7/Fig7D/P05_images/P05_Irradiated lesion_post.tiff]

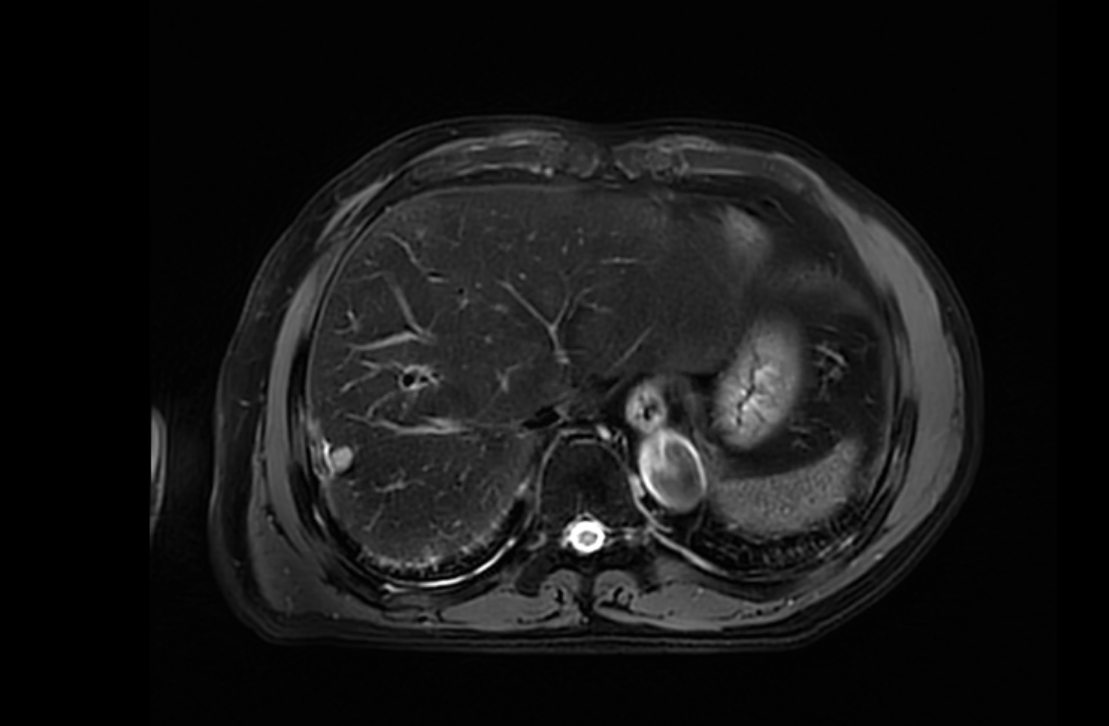

Supplement: Supplementary file 15 — Source data Fig. 7 [file 44321_2024_68_MOESM15_ESM.zip › Figure 7/Fig7D/P05_images/P05_Irradiated lesion_pre.tiff]

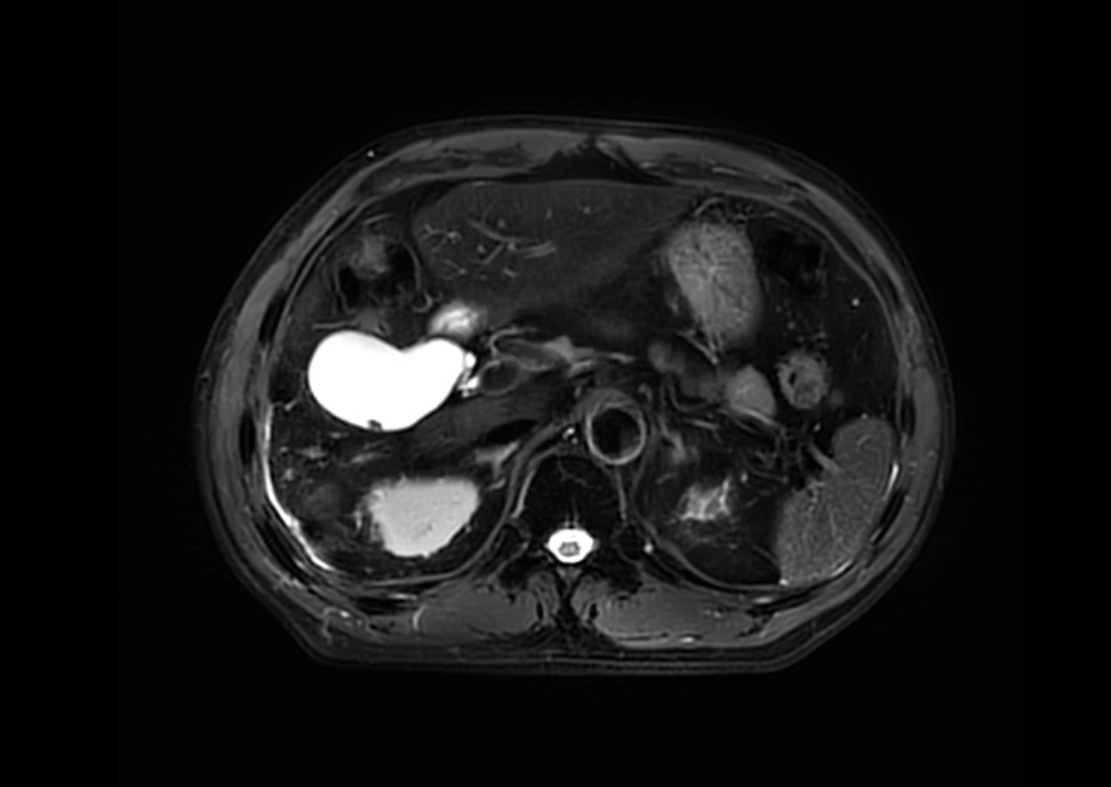

Supplement: Supplementary file 15 — Source data Fig. 7 [file 44321_2024_68_MOESM15_ESM.zip › Figure 7/Fig7D/P05_images/P05_Distant lesion_Post.tiff]
