# Supplementary material for: TLR3 activation enhances abscopal effect of radiotherapy in HCC by promoting tumor ferroptosis
Source: EMBO Mol Med. 2024 Apr 26;16(5):8. doi: 10.1038/s44321-024-00068-4 (PMC11098818; doi:10.1038/s44321-024-00068-4)
Supplement: Supplementary file 17 — Expanded View Figures [file 44321_2024_68_MOESM17_ESM.pdf]

Expanded View Figures

Figure EV1. Effects of Fer-1 and NAC on tumor ferroptosis and tumor control induced by poly(I:C) plus RT in HCC mice model.

(**A**) Tumor growth curves of directly irradiated tumor (Tumor1) and distant tumor (Tumor2) in different treated groups ($n = 5$ mice for each group) as indicated. Results are shown as mean ± SEM (error bar), and ANOVA was performed to analyze the differences among groups. (**B**) Schematic diagram of ROS inhibitor NAC treatment in HCC tumor mouse model receiving poly(I:C) plus RT. (**C**) Relative ROS level of Tumor1 and Tumor2 (obtained from mice sacrificed on day7 of the treatment procedure) in different treated groups ($n = 5$ independent samples for each group) as indicated (illustrated by scatterplot). The data are shown as mean ± SEM (error bar), and ANOVA was performed to analyze the differences among groups, and ANOVA was performed to analyze the differences among groups. (**D**) Tumor growth curves of directly irradiated tumor (Tumor1) and distant tumor (Tumor2) in different treated groups ($n = 5$ mice for each group) as indicated. Results are shown as mean ± SEM (error bar), and ANOVA was performed to analyze the differences among groups. Determination of MDA content (**E**) and GSH content (**F**) in Tumor1 and Tumor2 collected from different treated groups ($n = 5$ independent samples for each group) as indicated (illustrated by scatterplot). The data represent the mean ± SEM (error bar), and ANOVA was performed to analyze the differences among groups.

© The Author(s)

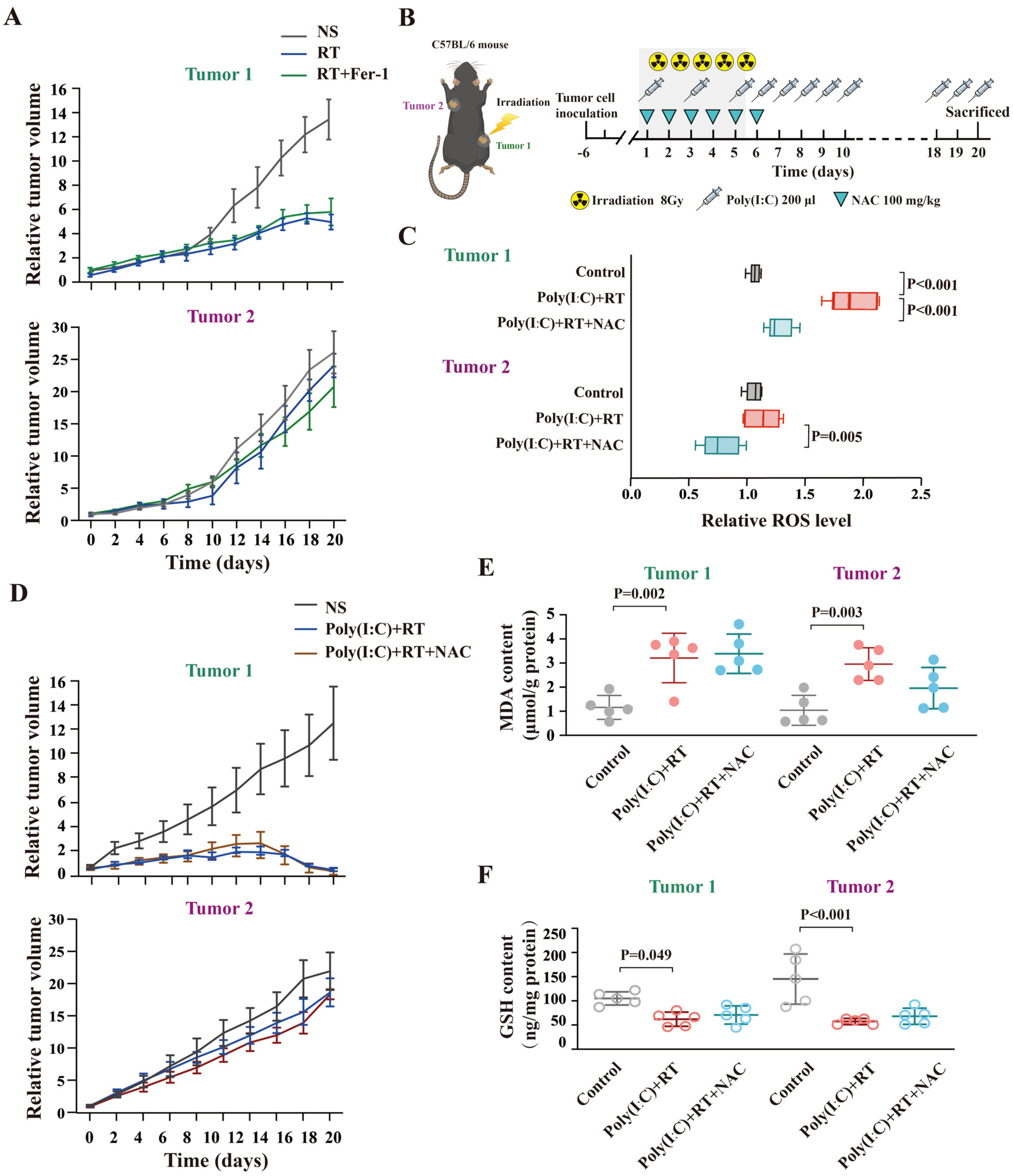
A
NS
RT
RT+Fer-1
Tumor 1
Relative tumor volume
Tumor 2
Relative tumor volume
Time (days)
B
C57BL/6 mouse
Tumor 2
Irradiation
Tumor 1
Tumor cell inoculation
Sacrificed
Time (days)
Irradiation 8Gy
Poly(I:C) 200 μl
NAC 100 mg/kg
C
Tumor 1
Control
Poly(I:C)+RT
Poly(I:C)+RT+NAC
P<0.001
P<0.001
Tumor 2
Control
Poly(I:C)+RT
Poly(I:C)+RT+NAC
P=0.005
Relative ROS level
D
NS
Poly(I:C)+RT
Poly(I:C)+RT+NAC
Tumor 1
Relative tumor volume
Tumor 2
Relative tumor volume
Time (days)
E
Tumor 1
Tumor 2
P=0.002
P=0.003
MDA content (μmol/g protein)
Control
Poly(I:C)+RT
Poly(I:C)+RT+NAC
F
Tumor 1
Tumor 2
P=0.049
P<0.001
GSH content (ng/mg protein)
Control
Poly(I:C)+RT
Poly(I:C)+RT+NAC

 © The Author(s)

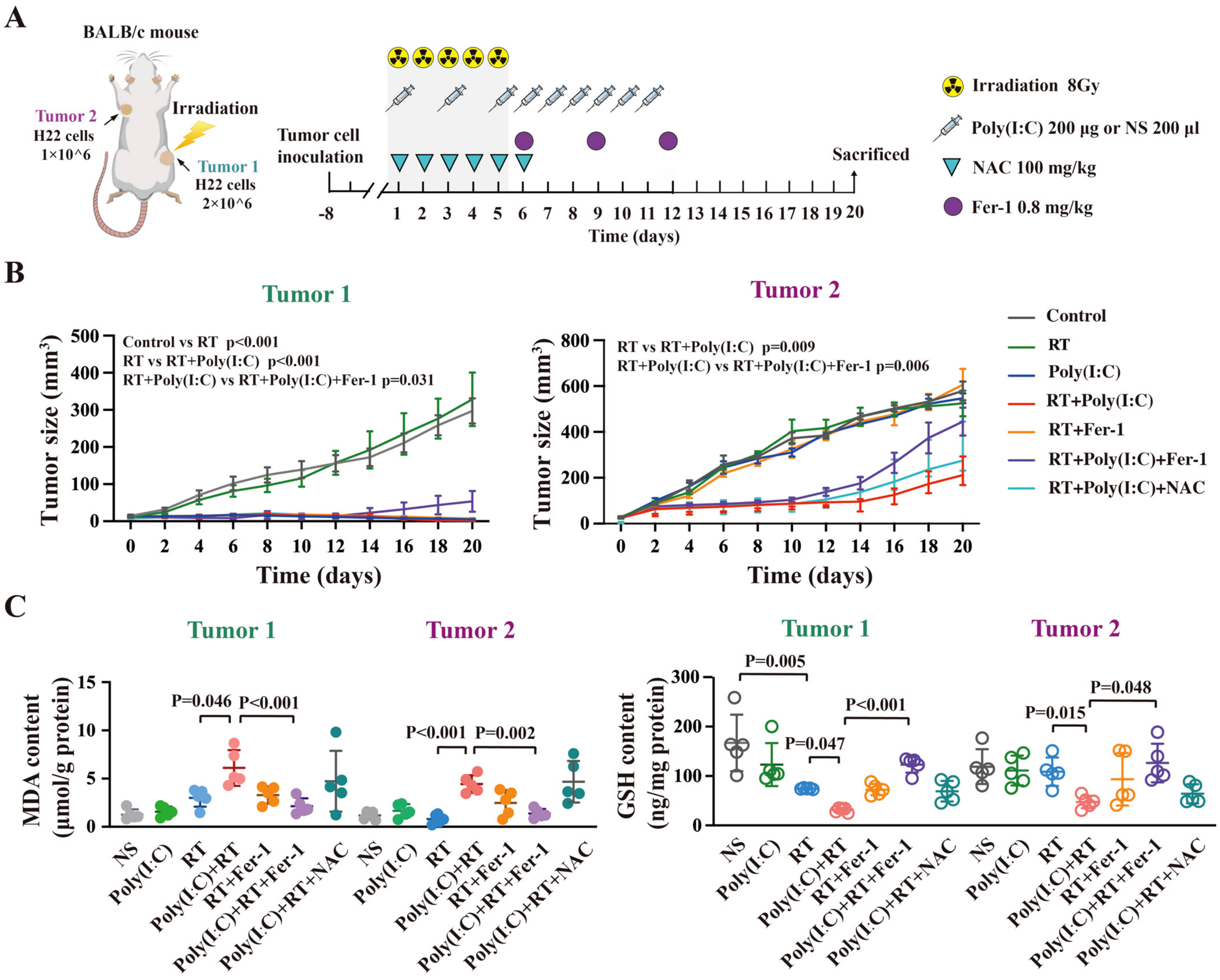

Figure EV2. Abscopal effect of radiotherapy enhanced by poly(I:C) stimulated tumor ferroptosis.

(**A**) Schematic diagram of modeling and therapy of bilateral HCC subcutaneous tumor model in BALB/c mouse. (**B**) Tumor growth curves of directly irradiated tumor (Tumor1) and distant tumor (Tumor2) in different treated groups ($n = 5$ mice for each group) as indicated. Results are shown as mean ± SEM (error bar), and ANOVA was performed to analyze the differences among groups. (**C**) Determination of MDA content and GSH content in Tumor1 and Tumor2 collected from different treated groups ($n = 5$ independent samples for each group) as indicated (illustrated by scatterplot). The data represent the means ± SEM (error bar), and ANOVA was performed to analyze the differences among groups.

© The Author(s)

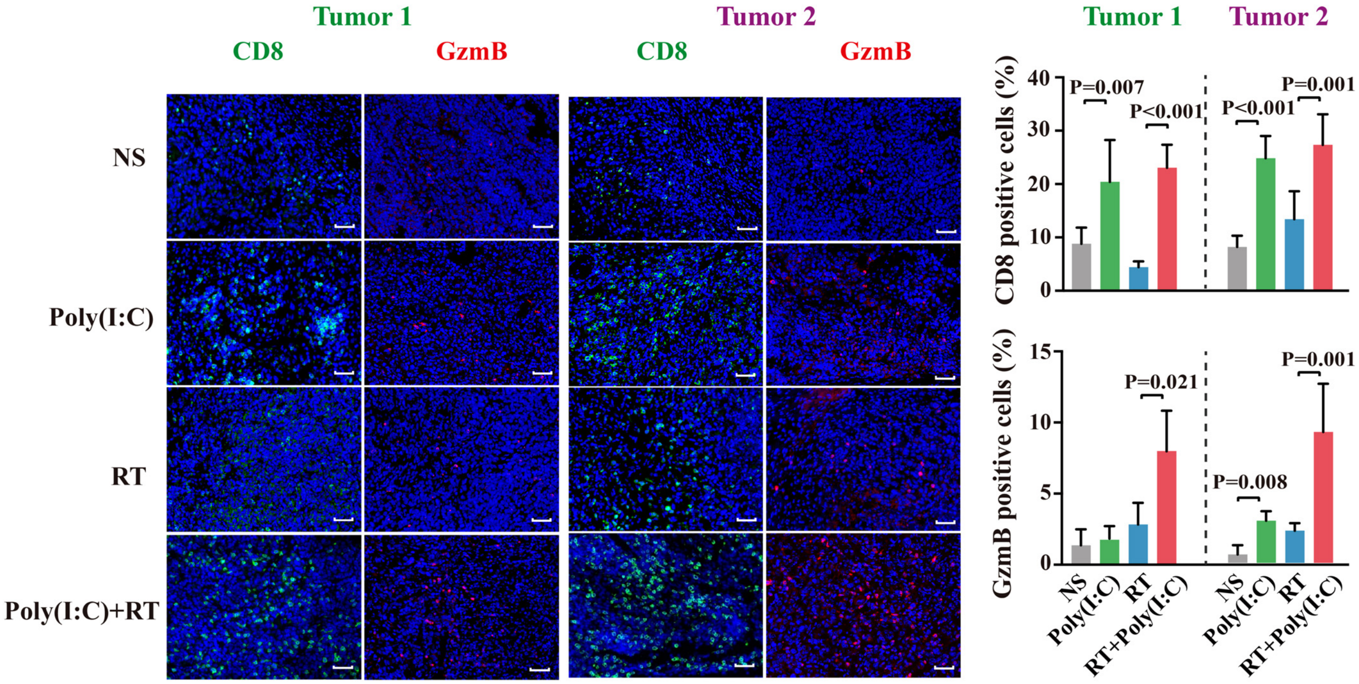

Figure EV3. Effects of poly(I:C) combined with RT on immune cell infiltration and vascular endothelial chemotaxis in tumors from HCC mice model.

The representative immunofluorescence image of $CD8^+$ T cells and GzmB in tumor tissues. Scale bars, 100 μm (100×). The proportion of CD8 positive cells and GzmB positive cells are represented by mean ± SEM(error bar) of 5 fields in the tumor tissue samples, and ANOVA was performed to analyze the differences among groups.

 © The Author(s)

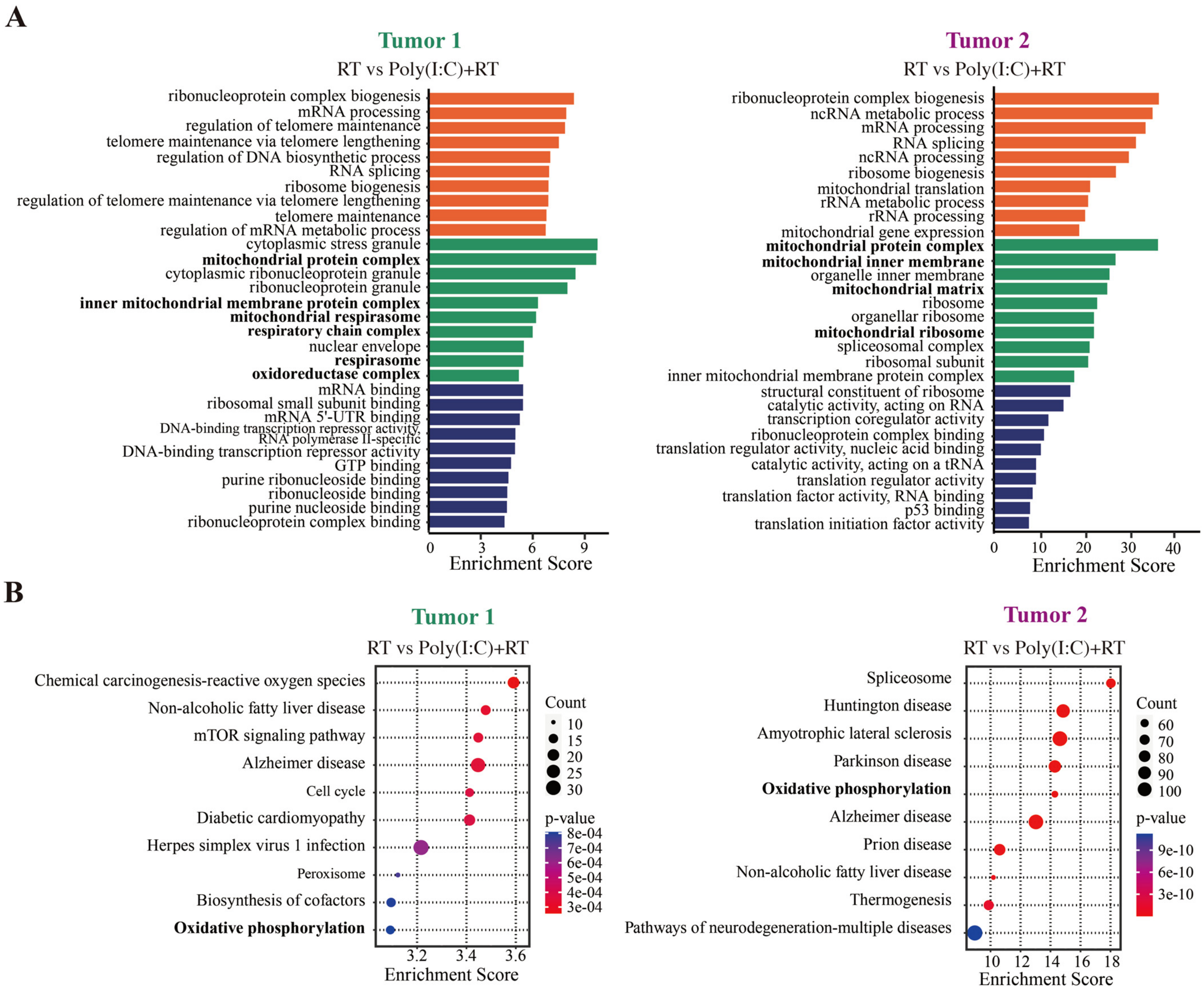

Figure EV4. Go annotation and pathway analysis of the differentially expressed genes in tumors derived from subcutaneous HCC mouse model receiving different treatments.

(**A**) GO analysis histogram. The horizontal axis represents the Enrichment score of the input genes in the Go annotation, while the vertical axis represents the name of Go annotation. Go annotation of differentially expressed genes with top ten Enrichment score covering domains of biological processes (Orange Brown), cellular components (Onion green), and molecular functions (navy blue). (**B**) Bubble map of the KEGG pathway enrichment analysis. The horizontal axis represents the Enrichment score of the input genes in the pathway, while the vertical axis represents the pathway name.

© The Author(s)

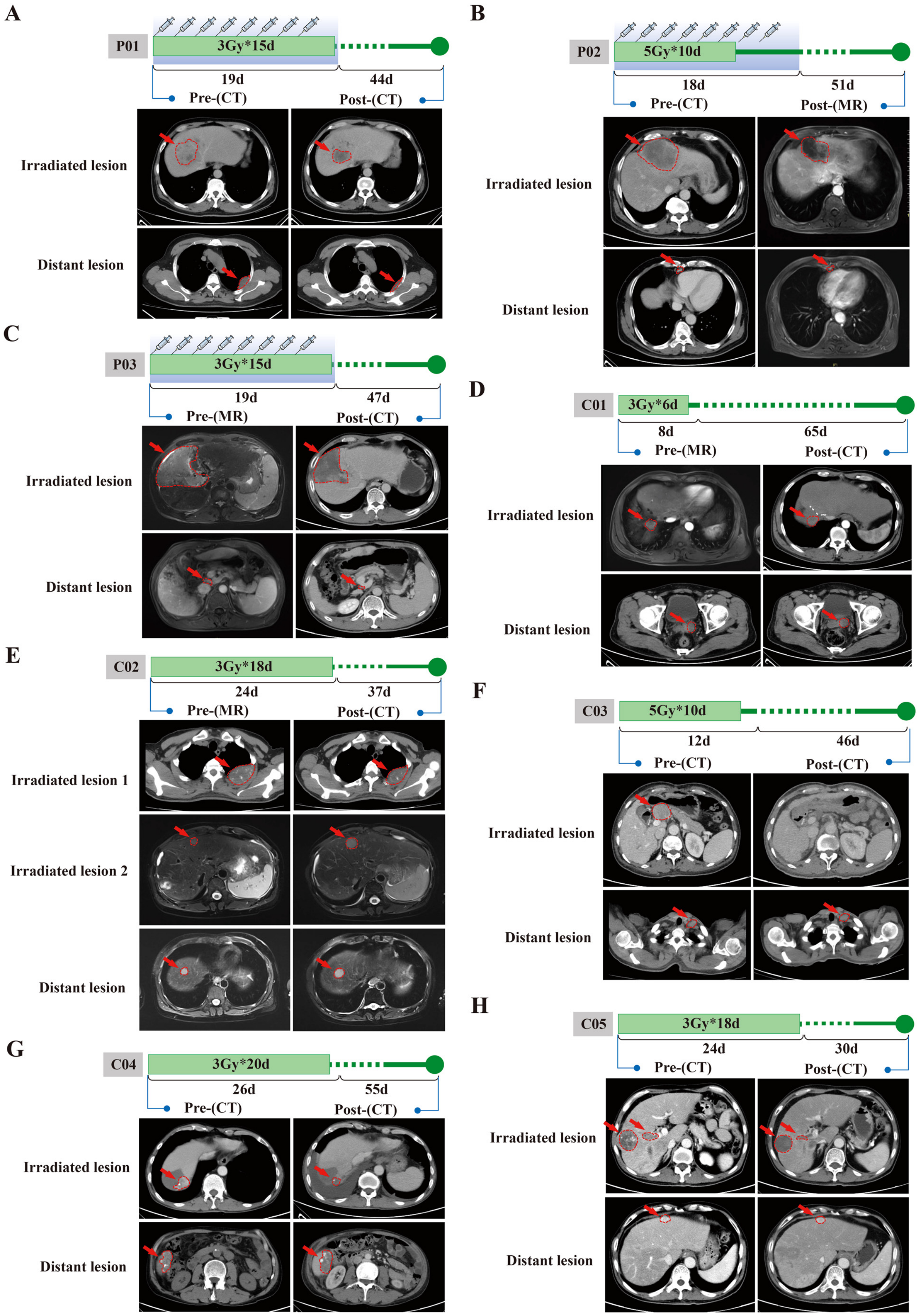
A
P01
3Gy*15d
19d
44d
Pre-(CT)
Post-(CT)
Irradiated lesion
Distant lesion
B
P02
5Gy*10d
18d
51d
Pre-(CT)
Post-(MR)
Irradiated lesion
Distant lesion
C
P03
3Gy*15d
19d
47d
Pre-(MR)
Post-(CT)
Irradiated lesion
Distant lesion
D
C01
3Gy*6d
8d
65d
Pre-(MR)
Post-(CT)
Irradiated lesion
Distant lesion
E
C02
3Gy*18d
24d
37d
Pre-(MR)
Post-(CT)
Irradiated lesion 1
Irradiated lesion 2
Distant lesion
F
C03
5Gy*10d
12d
46d
Pre-(CT)
Post-(CT)
Irradiated lesion
Distant lesion
G
C04
3Gy*20d
26d
55d
Pre-(CT)
Post-(CT)
Irradiated lesion
Distant lesion
H
C05
3Gy*18d
24d
30d
Pre-(CT)
Post-(CT)
Irradiated lesion
Distant lesion

 © The Author(s)

Figure EV5. Representative MR/CT scans of enrolled patients and control cases.

Tumor lesions were outlined by red dash circle and marked with red arrows on representative MR/CT scans of enrolled patients P01, P02, P03, and control cases C01, C02, C03, C04, C05. (**A**) Representative imaging scans of patient P01: the irradiated intrahepatic tumor lesion was reduced by 35.1%, while the non-irradiated lymph node metastasis was reduced by 49.0%. (**B**) Representative imaging scans of patient P02: the irradiated tumor located on the left lobe of liver was reduced by 25.7%, while the non-irradiated cardiac phrenic horn lymph node metastasis was reduced by 49.9%. (**C**) Representative imaging scans of patient P03: the total volume of irradiated tumor lesions (located on the right lobe of liver and portal vein) was reduced by 31.2%, while the non-irradiated retroperitoneal lymph node metastasis was reduced by 63.5%. (**D**) Representative imaging scans of patient C01: the irradiated tumor located on right lobe of liver was reduced by 7.3%, while the non-irradiated pelvic lymph node metastasis was reduced by 9.6%. (**E**) Representative imaging scans of patient C02: the irradiated lymph node metastasis located on the 2nd paracostal was reduced by 26.7%, while the non-irradiated intrahepatic tumor was increased by 33.2%. (**F**) Representative imaging scans of patient C03: the irradiated portal lymph node metastasis was eliminated, while the non-irradiated intraperitoneal lymph node metastasis was increased by 2.9%. (**G**) Representative imaging scans of patient C04: the irradiated tumor located on right lobe of liver (near to diaphragmatic surface) was reduced by 54.7%, while the non-irradiated intrahepatic tumor was increased by 8.5%. (**H**) Representative imaging scans of patient C05: the irradiated tumor located on right lobe of liver was reduced by 31.8%, while the non-irradiated tumor located on left lobe of liver was increased by 2.7%.

© The Author(s)
